# Society for Cardiovascular Magnetic Resonance/European Society of Cardiovascular Imaging/American Society of Echocardiography/Society for Pediatric Radiology/North American Society for Cardiovascular Imaging Guidelines for the Use of Cardiac Magnetic Resonance in Pediatric Congenital and Acquired Heart Disease: Endorsed by The American Heart Association

**DOI:** 10.1161/CIRCIMAGING.122.014415

**Published:** 2022-06-21

**Authors:** Mark A. Fogel, Shaftkat Anwar, Craig Broberg, Lorna Browne, Taylor Chung, Tiffanie Johnson, Vivek Muthurangu, Michael Taylor, Emanuela Valsangiacomo-Buechel, Carolyn Wilhelm

**Affiliations:** 1 Departments of Pediatrics (Cardiology) and Radiology, The Perelman School of Medicine, University of Pennsylvania, Philadelphia, PA, USA, (M.A.F.).; 2 Division of Cardiology, Department of Pediatrics, The Children’s Hospital of Philadelphia, Philadelphia, PA, USA, (M.A.F.).; 3 Department of Pediatrics (Cardiology) and Radiology, The University of California-San Francisco School of Medicine, San Francisco, USA, (S.A.).; 4 Division of Cardiovascular Medicine, Oregon Health and Sciences University, Portland, USA, (C.B.).; 5 Department of Radiology, University of Colorado, Denver, USA, (L.B.).; 6 Department of Radiology and Biomedical Imaging, The University of California-San Francisco School of Medicine, San Francisco, USA, (T.C.).; 7 Department of Pediatrics (Cardiology), Indiana University School of Medicine, Indianapolis, USA, (T.J.).; 8 Department of Pediatrics (Cardiology), University College London, London, UK, (V.M.).; 9 Department of Pediatrics (Cardiology), University of Cincinnati School of Medicine, Cincinnati, USA, (M.T.).; 10 Department of Diagnostic Imaging, University of Zurich, Zurich, Switzerland, (E.V.-B.).; 11 Department of Pediatrics (Cardiology), University Hospitals-Cleveland, Cleaveland, USA (C.W.).

**Keywords:** cardiac magnetic resonance, congenital heart disease, pediatrics, children, acquired pediatric heart disease

## Abstract

Cardiovascular magnetic resonance has been utilized in the management and care of pediatric patients for nearly 40 years. It has evolved to become an invaluable tool in the assessment of the littlest of hearts for diagnosis, pre-interventional management and follow-up care. Although mentioned in a number of consensus and guidelines documents, an up-to-date, large, stand-alone guidance work for the use of cardiovascular magnetic resonance in pediatric congenital 36 and acquired 35 heart disease endorsed by numerous Societies involved in the care of these children is lacking. This guidelines document outlines the use of cardiovascular magnetic resonance in this patient population for a significant number of heart lesions in this age group and although admittedly, is not an exhaustive treatment, it does deal with an expansive list of many common clinical issues encountered in daily practice.

## Introduction

### Background

The role of imaging and the modalities utilized in pediatric and congenital heart disease (CHD) is continually evolving. Cardiovascular magnetic resonance (CMR) is now a standard modality in imaging CHD and is considered a “one-stop-shop” with the capability of visualizing anatomy and assessing ventricular function, blood flow and tissue characterization. It is utilized in conjunction with other imaging modalities in almost all instances including echocardiography, invasive angiography, cardiac computed tomography (CT) and nuclear medicine. The spectacular improvements in diagnosis, treatment and follow-up in this patient population is in part due to the use of this multimodality imaging approach.

There is significant literature supporting the use of CMR in pediatric CHD and acquired heart disease, however, there is wide practice variation among centers for which patients undergo CMR. Availability, diagnostic accuracy, economics and patient burden all play a role in which imaging modality is utilized for various diagnostic categories in the different centers. Echocardiography has been and remains the front line imaging modality for most CHD patients, however, the objectives and frequency of use of echocardiography have changed with the increased utilization, established and evolving capabilities of CMR and cardiac CT.

Although there are current guidelines in adult CHD which involve CMR,^1^ pediatric CHD and acquired pediatric heart disease are unique and distinct entities which has different requirements and needs such as smaller structures, higher heart rates, complex unknown anatomy and the more pressing concern of avoiding ionizing radiation. Currently, there is only a consensus document that is available on CMR which is dedicated to pediatric CHD and pediatric acquired heart disease,^2^ an old CMR consensus documents with only small sections on pediatrics and CHD^3,4^ and an old appropriate use criteria (AUC) document with again, only small sections on pediatrics and CHD.^5^ A document describing technical protocols has been published but does not set forth guidelines or indications.^6^ Finally, CMR is included in the most recent AUC for multimodality imaging in the follow-up care of patients with CHD with given scenarios which is different than guidelines document for the use of CMR at all stages of care.^7^

### Purpose of this Guidelines Manuscript

The primary objective of this document is to present guidelines based on the existing literature supporting CMR for commonly encountered pediatric CHD and acquired pediatric heart disease. It is beyond the scope of this paper to delineate CMR physics, technical details and protocols focused on imaging children, as there are excellent guidelines for this published elsewhere.^6,8^ Where literature is sparse or non-existent, consensus opinion of the writing group is presented. The document includes both disease specific (e.g., single ventricle) and technique specific (e.g, ventricular function) sections focused on pediatric CHD and acquired heart disease. Each section includes a brief introduction followed by a review of the literature supporting use of CMR with formal recommendations for indications at the end of the section.

These guidelines are intended to assist providers in the decision to utilize CMR. They represent an extensive review of the available current scientific evidence. Many clinical scenarios are complex and some may not be covered exactly by the document; final judgement as to whether CMR is appropriate for a particular patient requires individualized decision making. In those situations, clinical decision making should consider the quality and availability of data in the area where care is provided. When these CMR guidelines are used as a basis for either regulatory or payer decisions, the goal should be improvement in quality of care. The writing group acknowledges that there may be some institutions that do not have access to or have expertise in CMR performance and as such, other imaging modalities such as cardiac CT may be considered.

This document is not a multimodality or cross-sectional imaging guideline for all the diseases mentioned or an in depth analysis of a comparison between imaging modalities. It is primarily a work on CMR indications. Where appropriate, a few comments are made regarding other imaging modalities. As a general rule, in emergency situations (e.g., pulmonary thromboembolism, shunt occlusion, and other unstable conditions) and in relative or absolute contraindications of CMR (e.g., presence of a pacemaker, a defibrillator, metals causing severe artifact or in claustrophobic patients who do not wish to be sedated) cardiac CT or cardiac catheterization may be considered.

### Selection of Writing Committee Members

A panel of acknowledged CMR experts was selected and rigorously reviewed by the Society for Cardiovascular Magnetic Resonance (SCMR) to develop these guidelines, to grade the level of clinical evidence and to write recommendations based on current knowledge of CMR and other imaging modalities. The writing group was composed of pediatric cardiologists and radiologists from both North America and Europe, representing different geographical regions, gender, ethnicities, races, perspectives and scopes of clinical practice. Representatives from the American Heart Association (AHA), the American Academy of Pediatrics and the Society for Pediatric Radiology were included in the writing group. Representation by an outside organization does not necessarily imply endorsement.

### Document Development Process

#### Relationships with Industry

Each member of the writing committee reported all relationships with industry and other entities relevant to pediatric CMR. Every effort was made by members to avoid actual, potential or perceived conflicts of interest.

#### Committee Meetings, Evidence and Literature Review

After numerous planning meetings, various sections of this document were written and developed by multiple committee members. Each section was distributed to the entire committee, reviewed, extensively discussed and edited at monthly meetings. All committee members had the opportunity to question and respond which allowed for rigorous debate. Final guideline recommendations were made by consensus agreement of the writing committee; the vast majority of recommendations were unanimous. When all sections were drafted, they were merged and sent out for review to the committee for final approval. Following peer review, the writing committee chair engaged authors to address reviewer comments and finalize the document for approval by participating organizations.

The recommendations listed in this document are evidence-based whenever possible. An extensive evidence review was conducted through March 2021. The literature searches were limited to studies conducted in human subjects and published in English. The references selected for this document are representative and not all-inclusive.

### Document Approval

The final version of the document was submitted to the SCMR publications committee and the SCMR Board of Trustees for review and approval. After their comments were incorporated and the document approved, the document was circulated to those organizations who contributed representatives to the writing committee (AHA, the American Academy of Pediatrics and the Society for Pediatric Radiology) along with the North American Society for Cardiac Imaging, the European Association of Cardiovascular Imaging and the American Society of Echocardiography to review this document and to give their approval.

### Class of Recommendation and Level of Evidence

These guidelines are classified using a standard evidence-based methodology developed by the AHA/ American College of Cardiology (ACC) Task Force.^9^ The class of recommendation (COR) is indicative of the strength of the recommendation which takes into account the estimated magnitude and certainty of benefit, in this case, medically relevant diagnostic information relative to the risk of CMR. The level of evidence (LOE) rates the quality of scientific evidence that supports the COR based on type, quantity and consistency of data from imaging studies. COR and LOE are determined independently. See Table 1.

**Table 1.**
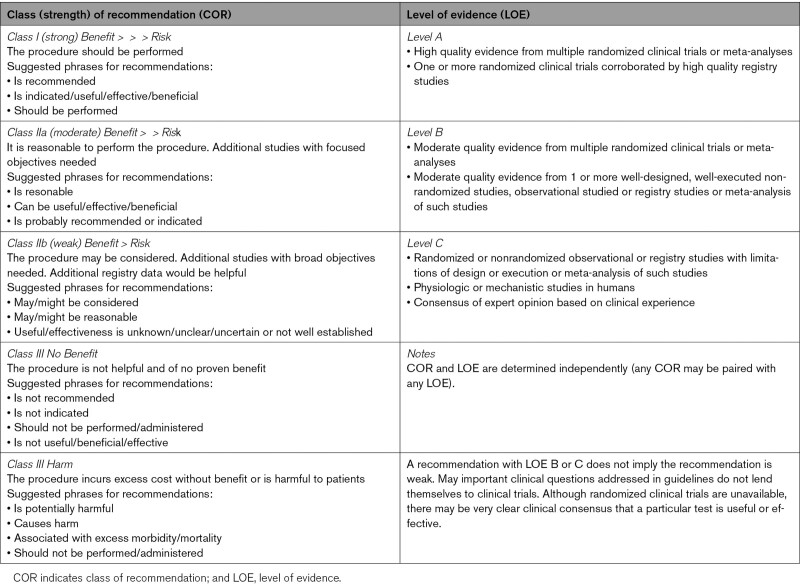
Class of Recommendation and Level of Evidence

The driving force for the development of these guidelines is based on an appreciation of the increasing use of CMR and a realization that the indications for pediatric CMR lack global consensus. Given the historical predominance of catheter based angiograms which chronologically was followed by echocardiography and the emergence of cardiac CT and CMR, it is clear there is a need for guidelines to optimize use of CMR. Although the guideline committee was aware of the lack of high levels of evidence with supporting randomized trials regarding pediatric CMR for many indications which is common for imaging modalities, a guideline document based on expert consensus with supporting literature nonetheless was deemed to be clinically useful.

## Diseases

### Single Ventricle

#### Background

The patient born with single ventricle (SV), where only one pumping chamber effectively exists, is one of the most complex of all CHD. Nearly all patients require reconstructive surgery or heart transplantation. During reconstructive surgery, which ultimately leads to the Fontan procedure,^10^ varying loads and physiology are imposed on the ventricle. To further complicate matters, SVs are not one lesion but rather a collection of many different types which fall under the same diagnostic category.

As an umbrella category for a vast array of lesions and with different terminology, it is difficult to state the exact incidence precisely. In one of the most comprehensive collections of studies on incidence, per million live births, a mean of 266 for hypoplastic left heart complexes, 222 for hypoplastic right heart complexes, 132 for pulmonary atresia, 79 for tricuspid atresia and 120 for “single ventricle” whose details were not delineated in studies was found.^11^ Hypoplastic left heart syndrome (HLHS) has been noted to occur in 0.016–0.36% of all live births and in pathologic series, represents 1.4–3.8% of CHD^12–14^ Tricuspid atresia prevalence ranges from 0.3 to 0.7% of all patients with CHD and occurs in ~ 1 in 15,000 live births.^15^

One of the major problems with a unified imaging strategy of SVs as a group is the variable anatomy; for example: (A) D-loop vs L-loop, (B) right (RV) vs left ventricle (LV), or (C) anatomic true SV versus a “functional” SV. As can clearly be seen, there can be a seemingly hopeless number of complex combinations, however, the underlying theme is that only one usable ventricle is present or both ventricles are connected in such a way that separating them into 2 pumping chambers is impossible.

**Figure 1.**
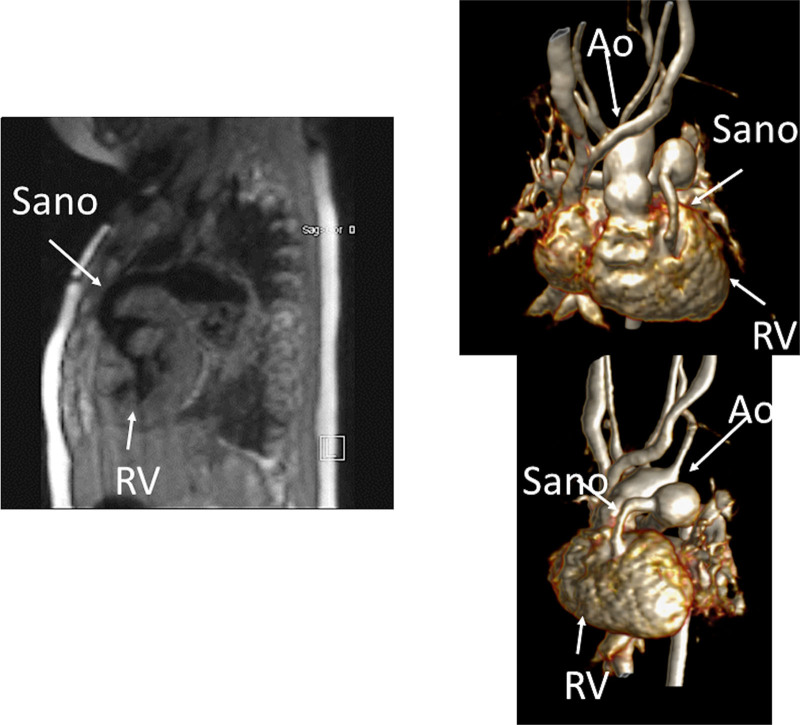
Hypoplastic left heart syndrome after Sano (right ventricle [RV] to pulmonary artery conduit). Left panel is a dark blood sagittal view and the right panels are 3D reconstructions demonstrating the entire length of the Sano shunt. Ao indicates aorta.

Another issue with a unified imaging strategy of SVs as a group is that during the various stages of surgical reconstruction, as noted above, the physiology of the cardiovascular system changes dramatically. The ultimate goal of surgery is to completely separate the systemic and pulmonary circulations and place them in a “series circuit.” In the native state, some patients, such as those with HLHS will always require surgical intervention—the Norwood Stage I procedure,^16^ which includes a systemic to pulmonary artery or ventricular to pulmonary artery (Sano)^17–21^ shunt (Figure 1), an atrial septectomy, and an aortic to pulmonary anastomosis. The SV pumps to both the systemic and pulmonary circulation in parallel, imposing a volume overload. Once pulmonary vascular resistance has dropped adequately (~ 3–6 months of age), a bidirectional superior cavopulmonary connection is performed. Since blood needs to go to the head/arms first before entering the pulmonary circulation, the ventricle does not pump directly to the pulmonary circulation and is therefore not technically volume loaded; it has been demonstrated, however, that systemic to pulmonary collaterals are present^22^ which can be quantified by CMR and puts a volume load on the ventricle.^23^ At approximately 2–5 years of age, directing inferior vena cava (IVC) blood into the lungs is performed to complete the Fontan operation.

Finally, a third issue with a unified imaging strategy is that surgical reconstruction can vary greatly. To perform an aortic to pulmonary anastomosis, a Norwood or Damus-Kaye-Stansel procedure can be used. For a bidirectional superior cavopulmonary connection, a hemiFontan or bidirectional Glenn (BDG) can be performed. For a Fontan, a myriad of ways have been employed as modifications such as a lateral wall tunnel, an extracardiac conduit, or an atrio-pulmonary connection (not performed anymore), all with or without a fenestration.

#### Indication of CMR in SV

**Figure 2.**
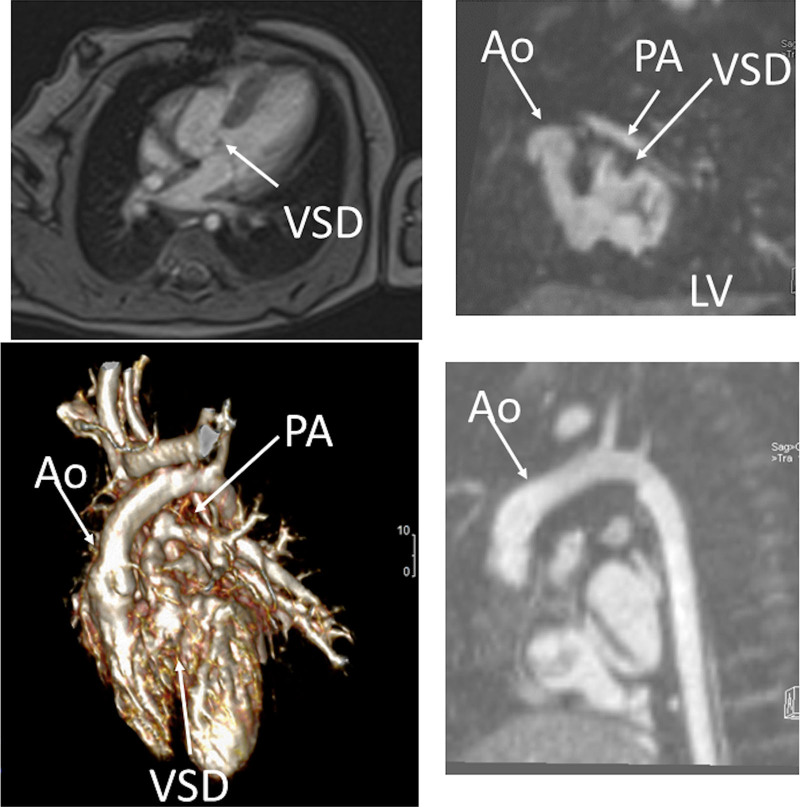
Three month old with double outlet right ventricle (DORV) being considered for a 1 versus 2 ventricular repair.Upper panels are 2 orthogonal views of the left ventricle (LV) to aortic (Ao) pathway through the ventricular septal defect (VSD). Lower left panel is a 3D model demonstrating a “4-chamber” view and DORV while lower right panel shows the anterior Ao. PA indicates pulmonary artery.

Prior to any surgery, CMR is not used frequently in the native state; generally, echocardiography is sufficient to allow for anatomic and hemodynamic characterization. Occasionally, if certain aspects of the anatomy are not delineated by echocardiography, such as pulmonary artery (PA) or pulmonary venous anatomy, CMR will be employed at this juncture (cardiac CT may be considered as an alternative if ventricle function, flow or tissue characterization information is not needed [ie anatomy alone], keeping in mind the radiation risk). In addition, if a “borderline” ventricle is present, CMR may be used to aid in the decision of a 1- versus 2-ventricle repair (Figure 2).

At all surgical stages, echocardiography is universally employed and at younger ages, this may be adequate. However, in older individuals, echocardiography many not be sufficient because of poor acoustic windows. In addition, cardiac catheterization may be used at all stages for diagnosis, however, it is invasive, incurs radiation and is not feasible to be utilized for routine follow-up.

##### Anatomy

CMR has been used for many years to evaluate the anatomy of the SV patient and has been validated against catheterization and surgical observation.^24–28^ This is performed in both 2D, 3D and now 4D formats with or without contrast media (Figure 1). For all stages of surgical reconstruction, CMR should be utilized to assess patients whose echocardiogram has not definitively demonstrated the anatomy listed in Table 2 for surgical planning. CMR should be utilized in place of invasive angiography for this anatomy unless an intervention is planned. CMR has been utilized for many years, dating back to the late 1980’s and early 1990’s, to delineate native viscero-atrial situs, intracardiac anatomy^29^ and ventriculoarterial connections and is now considered standard of care. Generally, when performing each stage of surgery, however, echocardiography for anatomy is almost always supplemented by another imaging modality such as CMR^27^ or in some institutions, catheterization or cardiac CT (Figures 1 and 2).

**Table 2.**
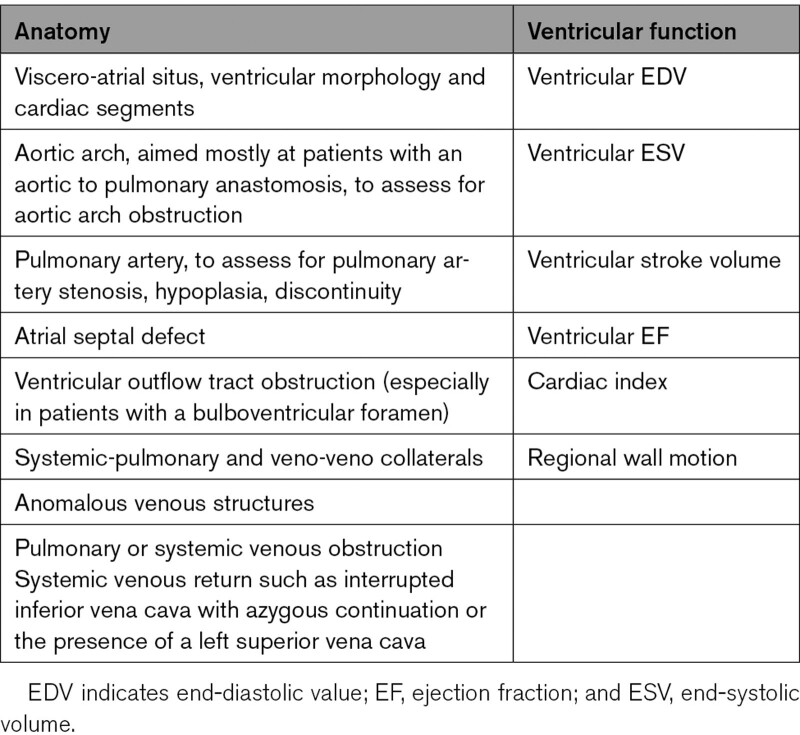
Anatomy and Ventricular Function Assessment in Single Ventricles

**Figure 3.**
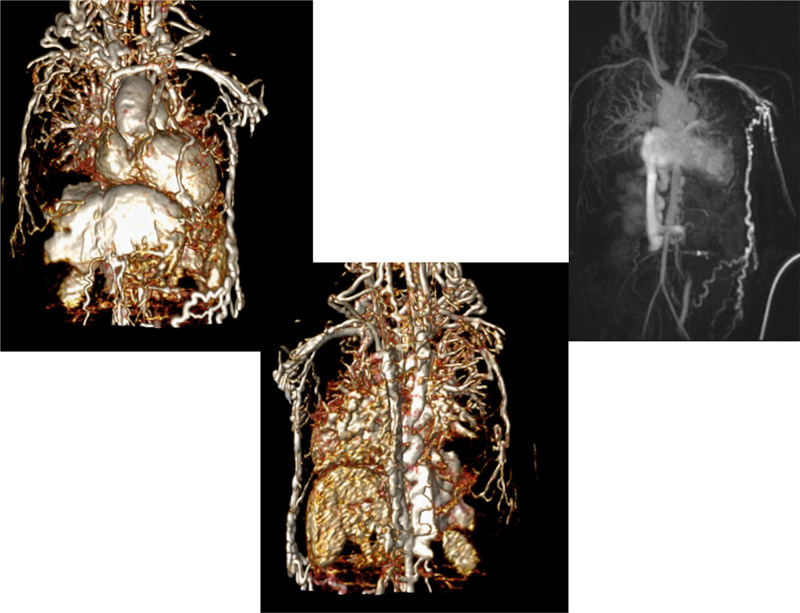
Massive systemic-to-pulmonary and venovenous collaterals in a 4 year old with pulmonary atresia and intact ventricular septum. Upper panels are maximum projection (right) and 3D reconstruction (left) of these collaterals viewed anteriorly while the lower panel is a 3D reconstruction of the collaterals as viewed from posterior.

At each stage of surgical reconstruction, in addition, certain aspects are focused on. Prior to the BDG/hemiFontan stage, CMR is directed towards evaluation of the aortic arch to assess for coarctation and the aortic to pulmonary anastomosis (if present). Further, pulmonary blood flow is delineated by visualization of the systemic to pulmonary or Sano shunt (if present), pulmonary stenosis, the pulmonary arteries and aortic to pulmonary collaterals. At the BDG/hemiFontan stage, besides reassessment of the aortic arch, the superior cava connections (e.g., right or left superior venae cavae (SVC) or Kawashima connections to the PAs) are visualized along with the pulmonary arteries, aortic to pulmonary and veno-venous collaterals (Figure 3). Finally, after the Fontan, the entire systemic venous pathway, especially the IVC to PA connection is focused on, including the branch pulmonary arteries.

#### Ventricular and Valve Function

**Figure 4.**
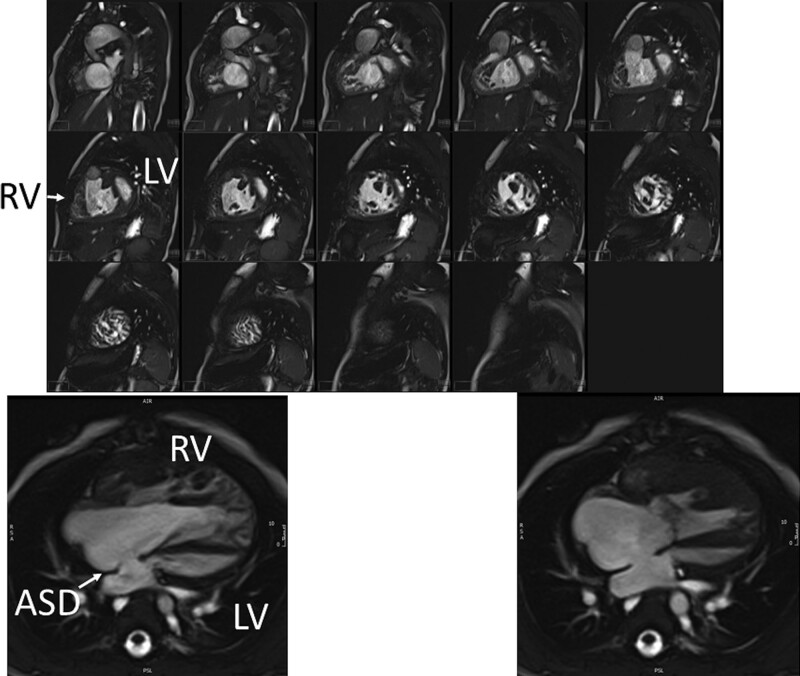
Ventricular function of an 18 month old with hypoplastic left heart syndrome. Upper panel is a short axis stack in diastole. Lower panels is a “3-chamber” view at end-diastole (left) and end-systole (right). ASD indicates atrial septal defect; LV, left ventricle; and RV, right ventricle.

CMR should be utilized to quantify 3D function which can be followed on a routine basis throughout all stages of surgical reconstruction and beyond. This includes regional wall motion abnormalities, ventricular volumes and mass, ejection fraction and cardiac index as delineated in Table 2. CMR has been the gold standard for biventricular volumes and function for many years and has been applied many times to the SV patient throughout staged surgical reconstruction (Figure 4).^30–35^ Ventricular performance parameters have been demonstrated to correlate with exercise performance^34^ and has been shown to correlate with transplant free survival after Fontan.^36^

Valve function, including atrioventricular and semilunar valve regurgitant volume and fractions, using phase contrast CMR (PC-CMR) or a combination of PC-CMR with ventricular volumes, should be assessed. PC-CMR has been used in the past to quantify valve function in CHD.^37–39^ Valve function is a significant issue in SV patients. For example, Mahle et al. has demonstrated that 6% of patients have moderate to severe atrioventricular valve regurgitation.^40^ Cohen et al. has shown that neoaortic regurgitation was present in 61% of patients up to 21 years of followup with progression in 49%.^41^

#### Physiology and Hemodynamics

PC-CMR has been used extensively in SV patients^42,43^ to assess physiology and hemodynamics. Important indices in the care of the SV patient are cardiac index as this is generally decreased, pulmonic flow (Qp)/systemic flow (Qs) which generally is close to one, flows to both lungs and systemic to pulmonary collateral flow^23,44–48^ which has been linked to short term outcomes such as hospital stay and presence of pleural effusions (see Qp/Qs and collateral flow section).^49^ In the BDG stage, cardiac catheterization cannot assess Qp because of systemic to pulmonary collaterals.^43^ Flows to both lungs are important parameters to determining the need for branch PA dilation, especially in SV patients where a patulous aortic reconstruction can compress the central PA. As mentioned in the forgoing paragraphs, PC-CMR is also used in the measurement of valve function.

#### Tissue Characterization For Myocardial Scarring

**Figure 5.**
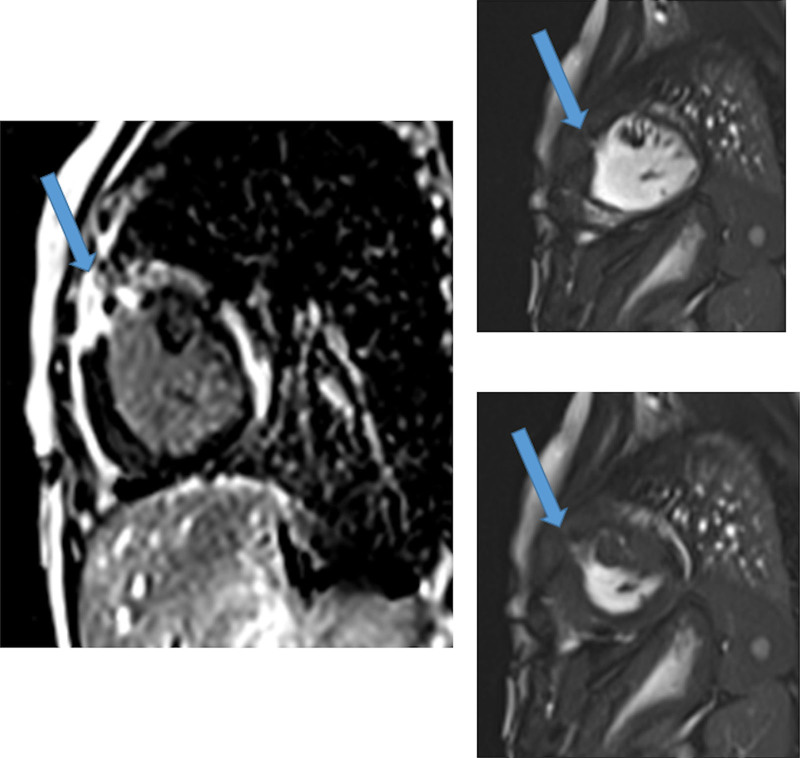
Myocardial scarring and regional wall motion abnormality in a 5 month old after a Sano shunt. Left panel is a phase sensitive viability image demonstrating the scar which is signal intense in the myocardium which should be signal poor (blue arrows). Right upper (diastole) and lower panels (systole) is the corresponding short axis view demonstrating the regional wall akinesia in the region of scar.

CMR has been utilized to evaluate both discrete myocardial scarring^50^ as well as diffuse fibrosis.^51^ Myocardial scarring may be an etiology for regional wall dysfunction as well as a nidus for arrhythmia. For example, diffuse fibrosis has negatively correlated with strain^51^ while discrete fibrosis has been linked to adverse ventricular mechanics and ventricular tachycardia.^50^ Myocardial scarring is commonly found around the os of the Sano shunt with accompanying regional wall motion abnormalities (Figure 5).

In part, because of the comprehensive assessment of anatomy, ventricular function, hemodynamics and tissue characterization that can be performed by CMR, a recent scientific statement from the AHA has recommended CMR be performed every 2–3 years after reaching the Fontan stage for evaluation.^52^

### CMR Prior to BDG and Fontan Reconstructions

In the past, a pre-operative echocardiography and cardiac catheterization prior to BDG and Fontan was the standard of care. In the past 15 years, however, it has been demonstrated that a select groups of patients can undergo CMR and echocardiography alone to safely undergo surgery.

In a retrospective study prior to BDG,^53^ Brown et al. studied the utility of cardiac catheterization in 114 SV patients, 51 of which were without suspected issues requiring catheterization after non-invasive imaging but nevertheless underwent the procedure. Only two had unsuspected findings, both of which were branch PA stenosis that could’ve been diagnosed by CMR. Twenty-five percent had complications from catheterizations, most of which were transient with 24% requiring transfusions and 14% needing an intensive care unit stay.

In a follow-up prospective trial, Brown et al.^54^ randomized 81 routine SV patients prior to BDG to CMR or cardiac catheterization and assessed the outcome after surgery. The cardiac catheterization group had more minor adverse events (75% vs 5%, P < 0.001), higher cost ($34,447 vs $14,921) and longer preoperative stay (2 vs 1 day) relative to the CMR group. There was one major adverse event in the CMR group in a patient with a Blalock-Taussig shunt who developed shunt thrombosis and required cardiopulmonary resuscitation and extracorpeal membrane oxygenation (ECMO); 4 days later the patient underwent routine BDG and was in good clinical status at 3-month follow-up. The operative course, the number of successful BDG and the frequency of postoperative complications were similar. At 3-month follow-up, there was no differences in clinical status, oxygen saturation or frequency of reintervention.

Prior to Fontan, Ro et al.^55^ studied 99 SV patients retrospectively and listed a set of criteria to determine who might benefit from cardiac catheterization and who may be able to safely proceed to surgery without it. These criteria were clinical as well as echocardiographic based and 46 fell into the category of those who could forgo catheterization. The criteria identified all patients who died or did not proceed to Fontan as well as 9 of 11 who required intervention; it had a negative predictive value of 93% (those who can forgo catheterization) with a sensitivity of 81%. However, the positive predictive value was only 25% and the specificity only 52% and the authors thought that this may be partly due to the inability of echocardiography to adequately assess the branch PAs. They suggested the addition of CMR would substantially increase pre-operative predictive values.

Another study assessed 3 groups prior to Fontan^27^ (119 patients in total); all patients underwent echocardiography, however, 41 patients underwent CMR only, 41 patients underwent catheterization only and 37 patients underwent both catheterization and CMR. No clinically significant differences were noted in patient characteristics, hemodynamics or clinical status prior to or after surgery between the CMR only and the catheterization only groups with CMR adding information in 82% of patients. Parameters such as cardiopulmonary bypass time, circulatory arrest time, days in the intensive care unit, other surgical procedures, surgical complications, interventions after Fontan, the incidence of pleural effusions, length of stay in the hospital and oxygen saturation at discharge were similar in all 3 groups. Diagnostic success at surgery relative to all imaging modalities was ≥ 95%. In the group that had both CMR and catheterization, measurements of blood vessels were similar and there were no discrepant findings. Echocardiography could not delineate completely the pulmonary arterial anatomy in 46–53% of patients.

### Summary of Recommendations

- Preoperatively or prior to commitment to either a univentricular or biventricular circulation, CMR is reasonable to determine anatomy, physiology and ventricular function not elucidated by echocardiography or to aid in determining one vs. two ventricle repair (Class IIa, Level of evidence B).
- Prior to BDG, if there is no primary indication for an intervention or there is no indication of increased pressures or pulmonary vascular resistance by echocardiography, CMR is indicated to determine anatomy, physiology, hemodynamics and ventricular function for use in surgical planning in routine cases (Class I, Level of evidence B). See Table 2
- Prior to Fontan, if there is no primary indication for an intervention or there is no indication of increased pressures (e.g., end-diastolic or Fontan pressures) or pulmonary vascular resistance by echocardiography, CMR is indicated for use in surgical planning in routine cases (Class I, Level of evidence B) (See Table 2).
- After Fontan, CMR is beneficial to follow asymptomatic patients routinely (Class I, Level of evidence B) every 2–3 years, especially when they reach the teenage years and is indicated in the symptomatic patient if there is no primary indication for an intervention or there is no indication of increased pressures (eg end-diastolic or Fontan pressures) or pulmonary vascular resistance by echocardiography
- Prior to surgery or at any stage of surgical reconstruction, CMR can be useful to evaluate anatomy and ventricular function including volumes and mass and valve function (Class I, Level of evidence B). Tissue characterization such as late gadolinium enhancement (LGE) may be useful in prognostication (Class I, Level of Evidence B)
- Prior to surgery or at any stage of surgical reconstruction, CMR can be useful to evaluate hemodynamics such as flows, cardiac index, Qp/Qs, flows to both lungs, fenestration flow (if Fontan) and systemic to pulmonary collateral flow (Class I, Level of evidence B).

## Tetralogy of Fallot

### Background

Tetralogy of Fallot (TOF)^56^ is the most common cyanotic CHD and has a prevalence of ~ 6% of all CHDs^57^ and an average incidence of 32.6 per 100,000 live births^11^ (~ 1660 babies born each year with TOF in the United States^58^). The main pathologic basis is antero-cephalad deviation of the developing conal septum which causes a malalignement type ventricular septal defect (VSD), resulting in an “overriding aorta’’ and right ventricular (RV) outflow tract (RVOT) obstruction, ultimately leading to RV hypertrophy. Repair typically consists of VSD closure and relief of RVOT obstruction, typically by placement of a transannular patch, which in most instances results in severe pulmonary regurgitation (PR) from disruption of pulmonary valve integrity; RV volume overload typically ensues.^59^ Another commonly used approach is placement of an RV to pulmonary artery conduit instead of a transannular patch which may also result in PR and RV volume overload. Definitive repair is generally performed in infancy with survival rates of > 98% in multiple series.^60–65^ Because of the high success rate in childhood, the number of repaired TOF patients has been increasing over the years with adult survivors of TOF repair now outnumbering children in a number of regions.^66^ The 30 year survival rate is > 90%^67,68^

Despite these successes, complications related to residual anatomic and hemodynamic abnormalities are nearly universal. In the vast majority of patients, as mentioned, relief of the RVOT obstruction leads to PR and RV volume overload with resultant reduced RV and LV performance and are at risk for poor clinical outcomes. Multiple studies that have investigated resting RV and LV function after TOF repair^69–74^ consistently found diminished RV and LV performance with decreased RV ejection fraction (RVEF) and LV ejection fraction (LVEF), mostly in patients with PR. Patients with RV volume overload are at risk for sudden death, ventricular arrhythmias, increased New York Heart Association (NYHA) class and decreased exercise performance.

Exercise capacity is significantly decreased in TOF survivors and deserves special attention.^75–77^ This exercise incompetence may result from either primary LV dysfunction or by “ventricular-ventricular” interaction, where the dilated RV impinges on LV geometry causing poor performance.^78–88^ When TOF patients were studied at rest and during exercise testing, the incremental exercise response of LVEF in TOF patients was depressed relative to controls and LVEF during exercise correlated with both RV end diastolic volume index (RVEDVI) and the severity of PR.^77^ When comparing exercise performance in TOF patients and controls, significant differences exist in peak workload, maximal heart rate and systolic blood pressure.^76^ A review of 22 exercise studies^89^ found that 14 showed a significant relationship between PR with abnormal RV function and decreased exercise capacity. Further implicating RV volume overload are studies that demonstrate once the RV volume overload is abolished by pulmonary valve replacement (PVR), exercise tolerance improved.^87,88^

Numerous other residua can be present. Residual or recurrent RVOT obstruction or pulmonary stenosis may be present at any age and commonly occur in the first several years after the initial repair; RV to PA conduits commonly need to be upsized as the patient grows and later on may become calcified and stenotic. Scar tissue from surgical relief of the infundibulotomy as well as the use of a patch to enlarge the RVOT results in non-contractile myocardium which may progress to aneurysm formation. Residual atrial septal defects (ASD) or VSD, branch PA stenosis, tricuspid regurgitation as well as aortic dilation and aortic valve regurgitation may all occur. Arrhythmia and conduction disturbances are commonly encountered.^90^ A recent study suggests that TOF survivors have a higher degree of RV and LV diffuse fibrosis compared to normal, raising the possibility of an etiology for conduction disturbances or decreased exercise performance^91,92^; the degree and time course of this fibrosis has yet to be defined. Table 3 lists complications commonly seen in TOF.

**Table 3.**
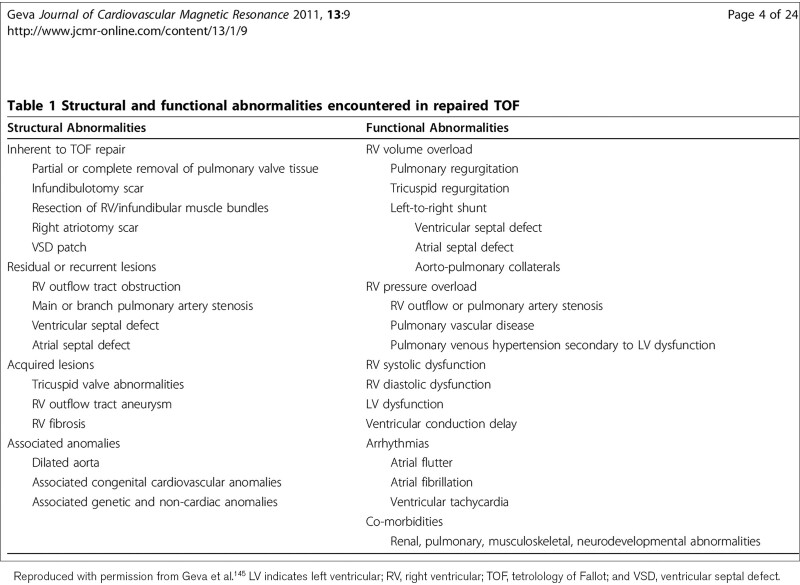
Complications of Repaired Tetralogy of Fallot

### Indication and the Role of CMR in TOF

**Figure 6.**
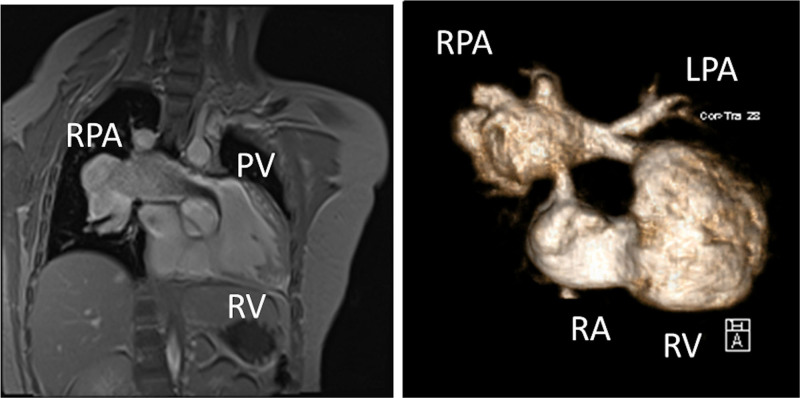
Severe right pulmonary artery (RPA) aneurysm in a 14 year old patient with tetralogy of Fallot and pulmonic stenosis. Left panel is an unbalanced gradient echo cine image and the right panel is a 3D reconstruction; note the turbulence in the main pulmonary artery from the stenotic pulmonary valve (PV). LPA indicates left pulmonary artery; RA, right atrium; and RV, right ventricle.

**Figure 7.**
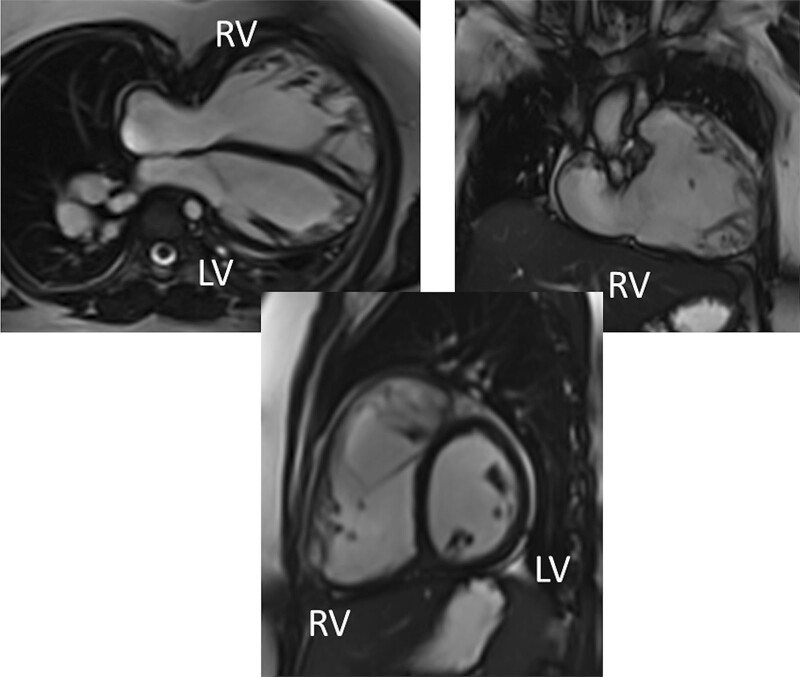
Ventricular function and volumes in tetralogy of Fallot. The 4-chamber (upper left), right ventricle (RV) two chamber (upper right) and short axis (lower panel) views of the patient in Figure 6 with volume overload of the RV. LV indicates left ventricle.

**Figure 8.**
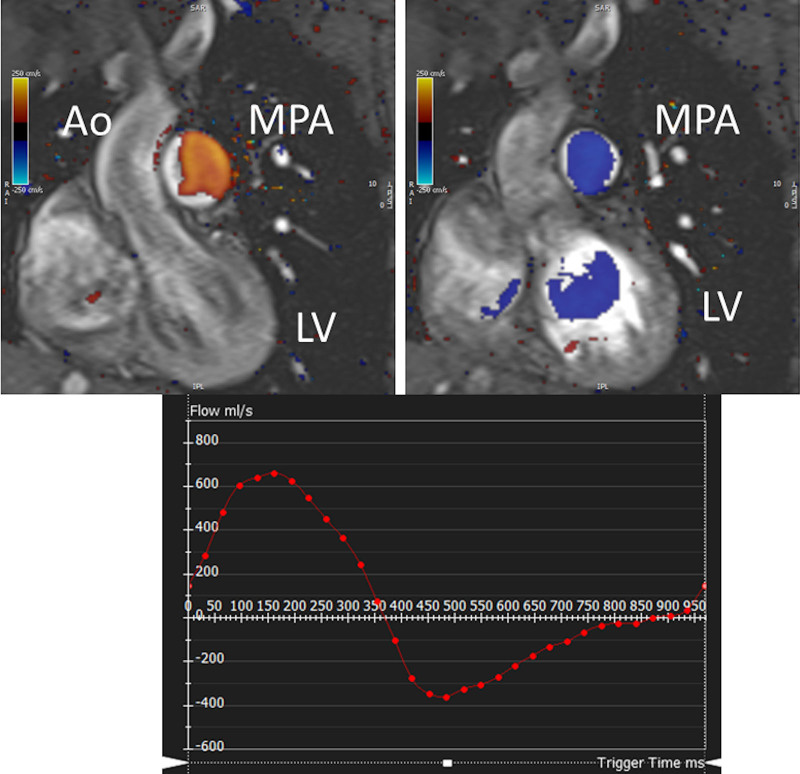
Color coded through plane PC-CMR of the main pulmonary artery (MPA) in systole (upper left, orange) and diastole (upper right, blue) demonstrating antegrade (orange) and retrograde flow (blue) signifying severe pulmonary regurgitation (PR).Lower panel is a flow (Y-axis) time (X-axis) curve demonstrating PR and antegrade end diastolic flow (after 900 mseconds). Ao indicates aorta; LV, left ventricle; ml/s, milliliters/second; and ms, milliseconds.

**Figure 9.**
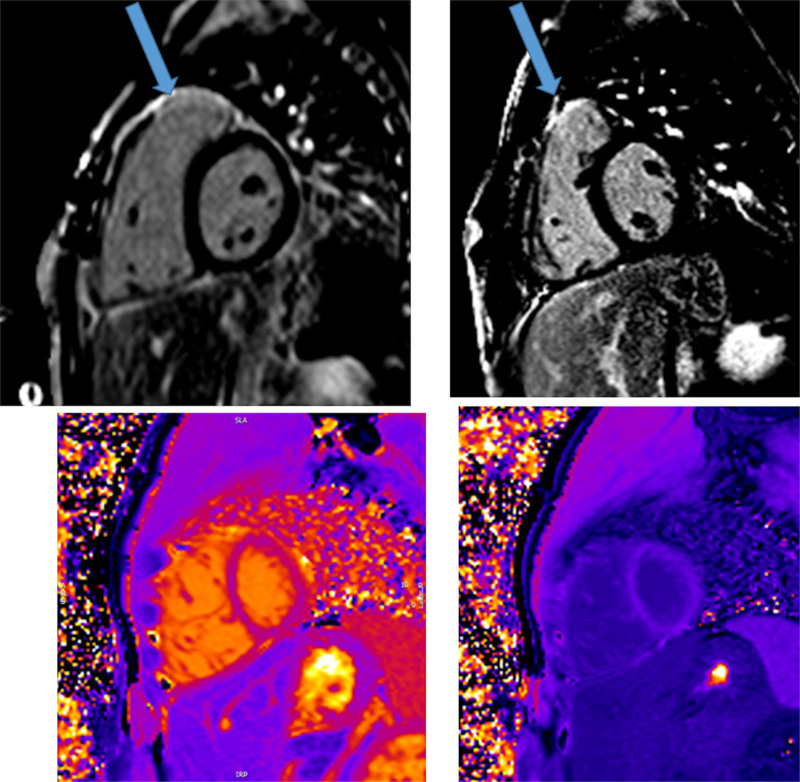
Discrete (upper panels, arrows) and diffuse fibrosis (lower panels) in a patient with tetralogy of Fallot. Two separate patients are demonstrated in the upper panels showing the areas of the transannular patch. Utilizing T1 mapping before (lower left) and after (lower right) gadolinium administration, extracellular volume can be quantified.

CMR has been utilized for years to assess anatomy (Figure 6), ventricular function including ventricular volumes (Figure 7), blood flow (Figure 8) and myocardial tissue characterization (Figure 9) in TOF survivors^91,92,95,96,98^ Multiple CMR techniques have been utilized for anatomical assessment of the RVOT, branch pulmonary arteries (PAs) (Figure 6) and aorta including electrocardiographically (ECG) gated balanced steady state free precession (bSSFP), unbalanced gradient echo imaging, dark blood imaging (which is much less susceptible to metal artifact) and contrast enhanced imaging to create 3D image sets. CMR is the gold standard for reliably and accurately measuring 3D ventricular volumes and performance generally utilizing bSSFP cine imaging and is the imaging modality of choice (Figure 7). PC-CMR^93^ is employed to measure flow and velocity, focused on PR (Figure 8), flow to both lungs, cardiac index, Qp/Qs, tricuspid regurgitation (alone or in combination with cine imaging) and aortic to pulmonary collateral flow. Parametric native T1 mapping^94^ can determine diffuse fibrosis and recent studies in children with repaired TOF have demonstrated extracellular volume (ECV) expansion^91,92^; in adult, TOF survivors showed a higher rate of adverse clinical events in TOF patients with ECV ≥ 30% than those with < 30% (Figure 9).^95^ Finally, myocardial strain by CMR using feature and tissue tracking allows for strain measurements with standard cine^96^ and has recently demonstrated to be prognostic in adult TOF survivors.^96^ Normal values for pediatric strain has recently been published.^97^

Prior to surgery in young children, echocardiography is primarily utilized for the management and care of the patient with TOF and CMR is generally not routinely indicated. There are a few exceptions such as:

- Lack of visualization of various structures such as the branch PAs by echocardiography
- aortic arch anomalies
- discontinuous branch PAs
- aorto-pulmonary collaterals (Figure 3)
- complex TOF or situs anomalies
- inconsistent clinical data that may indicate the need for an intervention other than routine repair.

After surgical repair, numerous sequelae can be present and CMR is indicated to assess nearly all of them:

*PR (Figure 8*) PR is a major issue and CMR is the only technique that allows for accurate quantification of not only of regurgitant volumes but regurgitant fraction as well (using PC-CMR)^98,99^ with echocardiography only having a modest correlation with CMR.^37^ It has been utilized since the early to mid 90 s for this evaluation^98,100^ and has been demonstrated to positively correlate with RV end-diastolic volume (RVEDV).^101,102^ In the absence of residual intracardiac shunts and other valve insufficiency, the difference in ventricular stroke volumes would equal the PR volume by PC-CMR.

*RV (Figure 7*) Cine CMR is the gold standard in determining quantitative biventricular size and mass and has been so for many decades.^32,103–106^ PR results in RV dilation with decreased function, risking morbidity and mortality,^107^ and the effects of RV dilation on LV function^108^ are important to follow by CMR. In a large cohort of patients spanning the gamut of ages, RV hypertrophy relative to RV volume was predictive of death and ventricular tachycardia.^109^ RVEF has been associated with impaired exercise performance.^110^ Typical values for RV dilation and hypertrophy in TOF have been published by many groups.^102,111–113^

It has been known for a number of years that intrinsic regional RV wall function is decreased in TOF survivors using CMR.^114^ Relatively recently, both RV and LV strain from routine cine CMR has been performed using either CMR feature tracking or tissue tracking of the myocardium. Both RV global longitudinal strain (GLS) and LV global circumferential strain (GCS) by CMR have emerged as predictors of poor outcome across a wide gamut of age ranges including pediatric and adolescents and may be useful in prognostication.^96^

Fibrosis has been noted by CMR in TOF survivors and has clinical implications. LGE or discrete fibrosis, has been utilized to assess viability of the myocardium for many years^115^ and in the TOF population, has been found to be present in both the RV and the LV. This increased signal intensity also occurs at the site of patch material such as the VSD and the transannular patch (Figure 9).^116^ Patients with poor ventricular performance, exercise intolerance and arrhythmias have demonstrated increased amounts of LGE throughout all age ranges^117,118^ and LGE in children positively correlates with increasing RVEDV and PR.^119^ RV diffuse fibrosis using T1 mapping has also been shown to be increased in TOF survivors in children,^91^ however, the significance is unknown at this time.

A published recommendation from the American Society of Echocardiography, developed in collaboration with SCMR and the Society for Pediatric Radiology recommends yearly CMRs based on RV performance parameters (eg RVEDVI ≥ 150 cc/m^2^, RVEF ≤ 48%) and every 3 years if the RV does not fall into these ranges for anyone 10 years of age or older; for those younger, it is ordered to address specific questions not addressed by echocardiography.^120^

*Left Ventricle* As mentioned above, numerous studies have documented LV dysfunction in repaired TOF patients for a few reasons and therefore, CMR evaluation of the LV takes on a key position in evaluation. CMR has demonstrated that this dysfunction is directly related to adverse outcomes such as ventricular tachycardia and death across all age ranges.^121^ LVEF has been associated with impaired exercise performance^110^ and as mentioned above, LV GCS has correlated with poor outcome.^96^ In addition, a small study has shown that LV diffuse fibrosis in children is associated not only with biventricular enlargement but is also associated with poor exercise performance^122^ and impaired LV mechanics^123^; long term clinical outcomes have yet to be elucidated.

*Anatomy* Important elements to image by CMR are residual lesions of the RVOT (e.g., RVOT aneurysm, the presence of an RV muscle bundle and RVOT and annular obstruction (Figure 7)), the branch PAs and surgical reconstructions such as RV to PA conduits.^124^ CMR in many instances is able to visualize these structures with higher fidelity than echocardiography, especially in the older child and adolescent. Although echocardiography is generally utilized to estimate the RV systolic pressure by measuring the peak tricuspid regurgitation (TR) velocity and the pressure drop across the RVOT and annulus by assessing the peak velocity by Doppler, in-plane PC-CMR may be utilized for this, although uncommon.

Since the mid to late 90’s CMR has been known to be a highly sensitive technique to assess the branch PAs in TOF.^125^ It has been validated against X-ray angiography^126^ and is superior to echocardiography.^127^ Branch PA stenosis or dilation (such as in TOF with absent pulmonary valve leaflets) should be noted. Physiologically, using PC-CMR, differential PA blood flow is obtained by CMR and has shown to be accurate^128–130^ even in the presence of stents,^131^ and may be used as a component in the decision making process to determine the need for intervention on the branch PAs.

*Left Sided Structures* Aortic root and ascending aortic dilation are known phenomenon seen in TOF and not only can significantly dilate in a high proportion of patients in the late teens and adulthood^132^ but also may cause considerable pathology.^133^ In addition, right aortic arches occur in ~25% of TOF along with branching abnormalities and the occasional vascular ring. These structures are routinely and easily imaged by CMR with and without contrast. Aortic regurgitation (AR), associated with aortic root and ascending aortic dilation, occurs in TOF^134^ and should be quantified by CMR^38^ using PC-CMR.

*Residual Shunting* Residual ASD and VSD flow can be present after surgical repair and can be diagnosed by echocardiography. CMR has utility not only visualizing these structures when inadequate echocardiography windows are present, but the strength of the modality is to quantify net shunting via PC-CMR with internal checks (see Qp/Qs section). In addition, in TOF patients with pulmonary atresia and multiple aortic to pulmonary collaterals, CMR again can visualize and quantify the shunt which has been performed since the 1990s.^135,136^

*Other Considerations* TR occurs not uncommonly in TOF and is also generally seen by echocardiography. CMR can quantify atrioventricular valve regurgitation in 2 separate ways for internal consistency and accuracy. Spatial relationships of the cardiovascular system and the airways can be important such as in TOF with absent pulmonary valve leaflets along with the relationship of the sternum in case of reoperation and CMR is useful in defining this anatomy. Coronary artery anatomy, for years a staple of CMR, can be defined as well in case of stenting the RVOT and main PA (see Coronary Artery section).

It should be noted that in certain circumstances, where the necessary airway or coronary anatomy cannot be obtained by CMR, or if visualization within a stent is needed for delineation of size, cardiac CT may be considered as an alternative.

*Pulmonary Valve Replacement* PVR deserves special attention in that it eliminates PR, decreases RV volume overload and improves symptoms including TR and exercise intolerance^87,88,137,138^ but the threshold ventricular volumes above which a PVR should be performed is unknown.^139–144^ Indexed end-diastolic volumes have ranged in various studies from 140 to 180 cc/m^2^. Other parameters to consider for PVR include large RVOT aneurysms, RVOT obstruction, sustained tachyarrhythmias related to RV volume overload, left to right shunt with a Qp/Qs > 1.5, severe AR or dilation.^145^ CMR has played a major role in attempting to determine the optimal timing of PVR and is indicated for baseline and follow-up evaluation of the TOF patient for PVR.

### Summary of Recommendations

- Prior to definitive TOF surgery, CMR can be useful to delineate various anatomic structures when there is a lack of visualization by echocardiography. In addition, it can be beneficial to delineate, aortic arch anomalies, discontinuous branch PAs, aorto-pulmonary collaterals and complex TOF anatomy or situs anomalies as an adjunct to echocardiography (Class IIA, level of evidence C).
- After definitive TOF repair, CMR is reasonable to delineate anatomy, physiology, blood flow, ventricular function and tissue characterization. In specific, assessing biventricular performance (ventricular volumes, ejection fraction, cardiac index), valve function (PR, TR, AR) and flows to both lungs are crucial to quantify (Class I, level of evidence B). RVOT, branch PA and aortic root/aortic anatomy are important to evaluate and measure (Class I, level of evidence B). Discrete myocardial scarring is important to identify (Class I, level of evidence B).
- CMR is indicated to evaluate RV volumes as a baseline, every 2–3 years if not dilated and ≥ 10 years of age or yearly if dilated and in the range to be considered for PVR (Class I, level of evidence B).
- Annual CMR is useful when surgery is being considered to evaluate RVOT aneurysms or obstruction, sustained tachyarrhythmias related to RV volume overload, left to right shunt with a Qp/Qs > 1.5, severe AR or dilation if being considered for PVR (Class IIA, level of evidence B).
- If the child requires sedation or anesthesia for CMR, this modality is reasonable to delineate anatomy, physiology, blood flow, ventricular function and tissue characterization when echocardiography suggests pathology or cannot visualize structures (Class IIA, level of evidence B). This can be performed as a baseline in childhood and prior to reaching the teenage years (Class IIB, level of evidence C).
- Myocardial strain (Class IIA, level of evidence B) and diffuse fibrosis (Class IIB, level of evidence C) by CMR might be considered for prognostication.

## Transposition of the Great Arteries

### Background

Transposition of the great arteries (TGA) is anatomically defined as a ventriculo-arterial discordance and is the second most frequent cyanotic CHD with a prevalence of 0.2–0.3 / 1000 livebirths with a male predominance of 1.5–3:1,^13^ accounting for 5–7% of all CHD.^146^ This section will focus on TGA with D-looped ventricles with repair using the arterial switch operation (ASO);L-looped TGA and repair with an atrial inversion operation is in the section on systemic RVs. The ASO is nowadays the surgical technique of choice for repair of TGA^147^ consisting of (1) transecting the aorta and the main PA at the level of the sinotubular junction, (2) removing the coronary ostia from the original aortic root and transferring them with a piece of surrounding tissue (button) to the neo aortic (pulmonary) root, (3) relocating the PA anteriorly and connecting it to the previous aortic root, and (4) relocating the aorta posteriorly and anastomosing it to the neoaortic root (native pulmonary root). With this technique, the branch PAs most commonly straddle the ascending aorta (LeCompte maneuver). Any additional intracardiac communication is closed during the surgery. This procedure allows both anatomical and functional repair restoring ventriculo-arterial concordance.

ASO can be performed successfully with low mortality rate.^148^ Nevertheless, potential postoperative complications include supravalvar and branch PA stenosis, coronary ostial occlusion/narrowing with subsequent myocardial ischemia and LV dysfunction, AR and neo-aortic root dilatation.^149^ Coronary artery complications after ASO have been reported in up to 10% of cases.^150,151^ Early detection of coronary artery lesions is essential for preventing ischemia and potentially life-threatening events. Notably, hearts after the ASO operation are denervated, and chest pain is not a reliable symptom of ischemia in these patients.^152^

Advanced imaging in patients with TGA after ASO is targeted to detect all potential residual findings requiring medical or surgical treatment. These include ventricular dysfunction, supravalvar pulmonary or aortic stenosis, branch PA stenosis, coronary artery stenosis/occlusion, neo-aortic or pulmonary valve regurgitation and, neoaortic root dilation.^153^ Even though there is one meta-analysis that concludes that coronary surveillance is not needed,^154^ multiple studies have concluded otherwise.^150–152,155^

### Indications for CMR

#### Prior to ASO

Echocardiography is the first line imaging modality prior to ASO and in most cases, CMR is not indicated. Occasionally there may be anatomic or physiologic abnormalities not delineated by echocardiography (e.g., branch PAs) and for those few cases, CMR is useful to delineate this missing information prior to surgery. When it is necessary to delineate the coronary anatomy or if echocardiography fails to do so, CMR has become more utilized; however, at the current time, it is not widespread and standard of care remains cardiac catheterization with cardiac CT as a backup.

#### After ASO

CMR is indicated and can depict almost all common potential residual findings after ASO (Table 4).

**Table 4.**
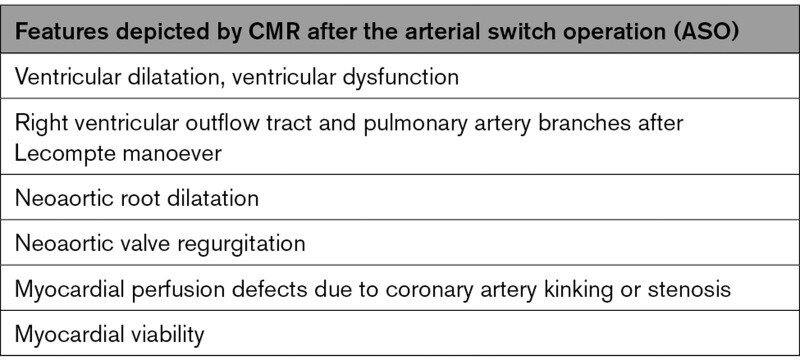
Features Depicted by CMR After the Arterial Switch Operation

### Ventricular Function

CMR is considered the modality of choice for quantification of biventricular volumes and function, especially the RV (Table 5).^6,8,156,157^ CMR has a high accuracy and reproducibility and is therefore the ideal modality for repeated measurements during follow up.^158,159^ In ASO patients, CMR can recognize diminished ventricular function in ASO patients at times when echocardiography fails to do so (Table 5).^160^ Moreover, advanced imaging with CMR can provide potential causes of ventricular dysfunction in the same examination (eg myocardial scarring, myocardial perfusion imaging if an adenosine stress CMR is being performed).

**Table 5.**
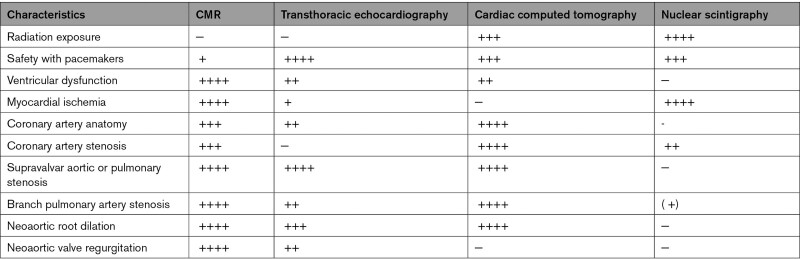
Comparison of CMR with Other Imaging Modalities as it Relates to Transposition of the Great Arteries

Mild biventricular dilation is not uncommon after the ASO operation. LV dysfunction has been observed in up to 20% of the cases and is correlated to clinical symptoms.^161^ In presence of ventricular dysfunction, concomitant evaluation of myocardial perfusion and scar imaging are essential for assessing coronary artery obstruction. RV dysfunction is rarer but can occur in combination with RVOT obstruction or stenosis of the branch PAs. Therefore, in presence of RV dysfunction, imaging the RVOT and the PAs is mandatory. Due to the position of the RVOT located immediately posterior to the sternum and of the branch PAs straddling the ascending aorta, visualization by transthoracic echocardiography (TTE) is rarely sufficient as patients grow and postoperative scar tissue often limit clear visualization of these structures.

### Coronary Arteries, Myocardial Perfusion and Viability

**Figure 10.**
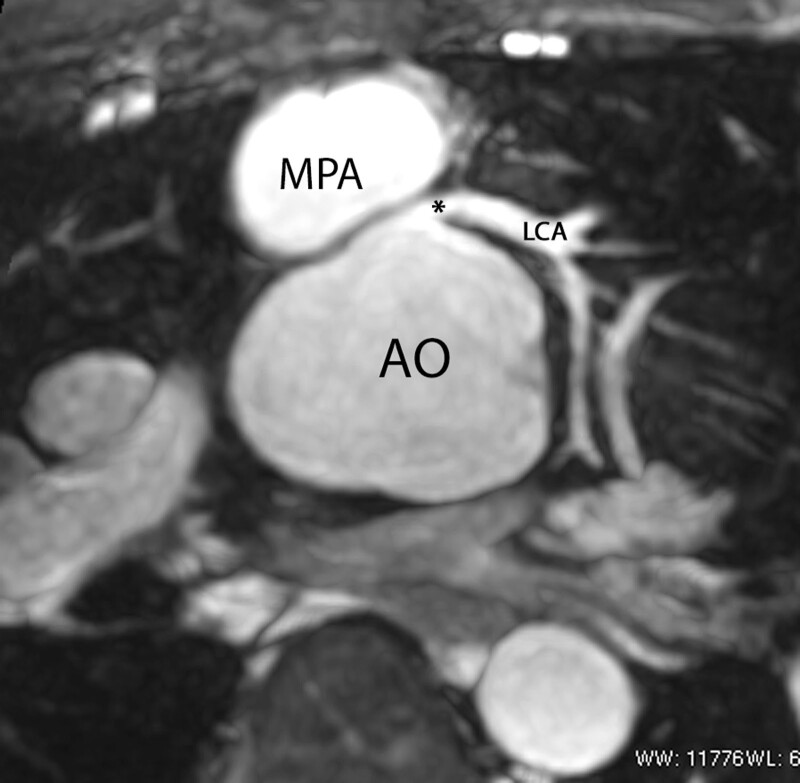
The coronary arteries after the arterial switch operation. 3D balanced steady state free precession (bSSFP) reconstructed image of the origin of the left coronary artery (LCA). The origin of the LCA (*) is occasionally wedged between the main pulmonary artery (MPA) and the aortic root (AO).

After coronary artery transfer by the ASO, the origin of both coronary arteries is usually in a different position than normal, facing the anteriorly positioned neopulmonary artery (Figure 10). Depending on its individual position, the proximal left coronary artery may show a tangential course which must be distinguished from true coronary artery obstruction. Moreover, it is still unclear whether this steep angle of origin may promote stenosis long term.^162,163^ Whole-heart CMR (3D balanced bSSFP, contrast enhanced inversion recovery gradient echo imaging using gadolinium or ferumoxytol) enables accurate detection of the abnormal origin and course of the coronary arteries even in very young patients with CHD^164,165^ and patients with TGA after ASO are no exception.^166^ Thus, evaluation of the coronary origins and courses routinely added to the CMR protocol^167^ (see section on CMR for coronary arteries).

**Figure 11.**
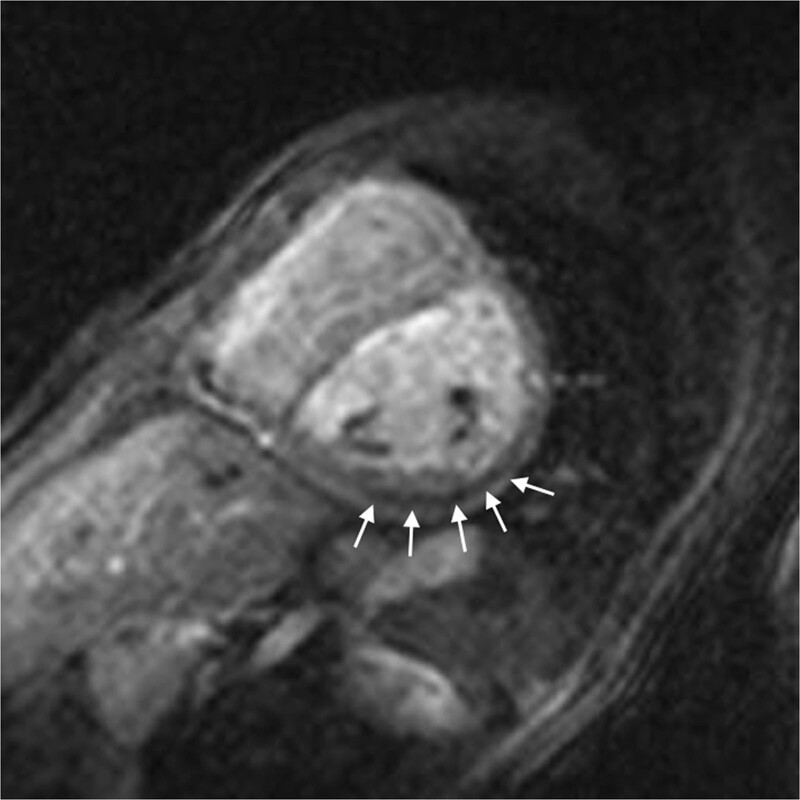
First-pass perfusion imaging. First-pass perfusion image showing a decrease intake of contrast-medium in the perfusion segments of the circumflex coronary artery in a 9-year-old boy after the arterial switch operation. The finding were confirmed at invasive coronary angiography.

In the cases with symptoms, LV dysfunction or coronary narrowing, evaluation of first-pass perfusion and viability can be performed by CMR.^168^ Myocardial perfusion, typically with the vasodilator adenosine,^169,170^ can be safely and accurately performed in children.^171–177^ In 56 myocardial first-pass perfusion scans performed in children, a sensitivity of 87% and a specificity of 95% have been described when compared with coronary angiography^171^ (Figure 11). Another group reported on 64 first-pass perfusion exams in 48 children and found a positive predictive value of 80% and a negative predictive value of 88% for detecting coronary lesions.^173^ There are some studies of TGA after ASO which did not find any scar or perfusion defects,^178^ however, there are others, using regadenoson as a vasodilator stress agent, which detected myocardial perfusion defects in up to 30% with very good agreement with coronary angiography.^179^

LGE can be found in up to 20% of the patients with TGA after ASO, some of which occur in a non-coronary pattern with small focal enhancement in the septal-free wall junction (possibly residuals from thromboembolic events during Rashkind maneuver and/or cardiopulmonary by-pass). Elevated diffuse myocardial fibrosis has been observed in a cohort of pediatric ASO patients^178,180^; the prognostic significance of this finding remains unclear.

### Pulmonary Arteries

**Figure 12.**
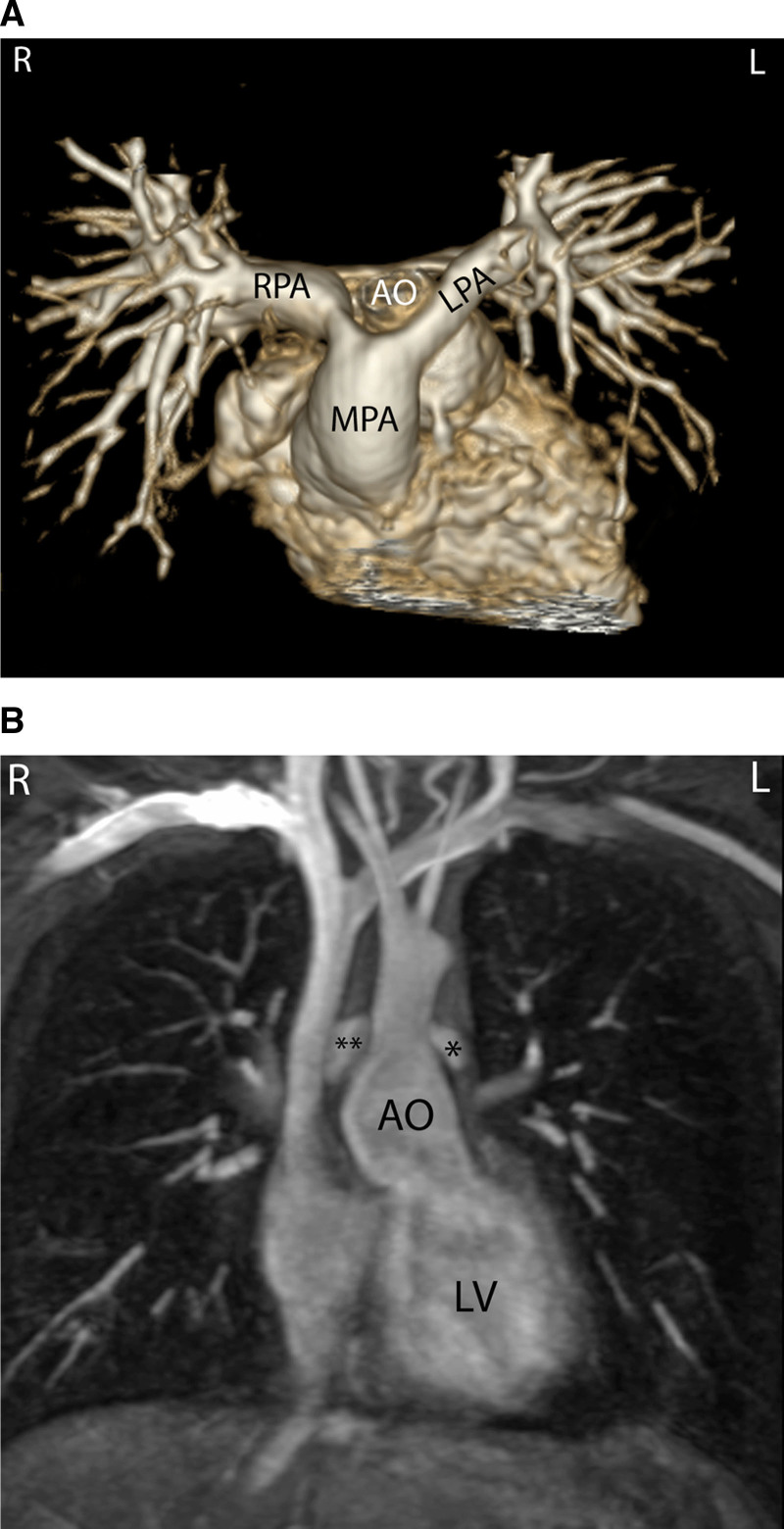
Geometry of the pulmonary bifurcation after Lecompte maneuver. **A**, Volume rendered 3D reconstruction showing that the pulmonary side branches runs around the aortic root. An external impression on the proximal course of the pulmonary arteries can occur. **B**, Coronal view of the geometric relation between ascending aorta and pulmonary arteries (RPA **, LPA *). Note the oval shape of the pulmonary arteries, with the broader diameter in the cranio-caudal direction and slimmer diameter in the lateral direction. Ao indicates ascending aorta; LPA, left pulmonary artery; and RPA, right pulmonary artery.

CMR is effective and superior to echocardiography for detecting complications of the PAs after the Lecompte maneuver.^181–183^ As the cross-section of the PAs is ellipsoid, the antero-posterior dimension is usually smaller than the supero-inferior one^184^ (Figure 12). PC-CMR measurements provide accurate quantitative differential lung perfusion and add crucial hemodynamic information to the anatomical images.^185–187^ An unbalanced lung perfusion > 70:30 is usually taken as cut off for the need of an intervention in the PA branches.^188^ By combining CMR anatomic findings with flow measurement, CMR has demonstrated that orientation of the neo-pulmonary root and diameter of the neo-aortic root are major determinants of the degree of branch PA stenosis.^189^ With 4D flow, the hemodynamics in the main PA and in branch PAs can be even better understood.^190,191^

### Neoaortic Root Dilation

**Figure 13.**
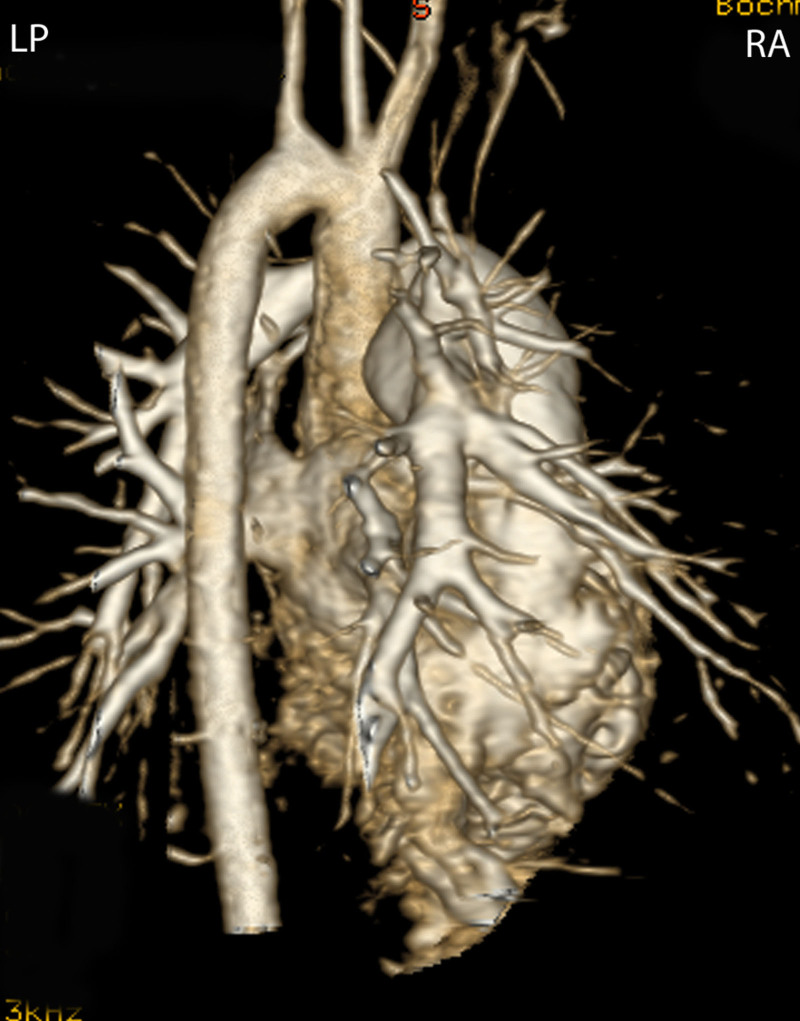
Geometry of the aortic arch after the arterial switch operation. Right-posterior view of 3D volume rendered angiography images showing the typical form of the aortic arch, consisting of a higher convexity, after the arterial switch operation.

Neoaortic root dilatation is a common finding during long term follow up and has been described by CMR in up to 76% of the patients.^161^ Neoaortic root dilatation progresses over time and is strongly associated with significant semilunar valve regurgitation. CMR is superior to other modalities for quantification of neoaortic valve regurgitation.^192^ Older age at time of ASO, presence of VSD, and previous PA banding are described risk factors for neoaortic valve regurgitation.^193–195^ CMR data has also demonstrated that aortic arch geometry (Figure 13) has a significant influence on the severity of neoaortic root dilation, with more acute aortic angles associated with larger neoaortic root and higher incidence of regurgitation.^196^

**Figure 14.**
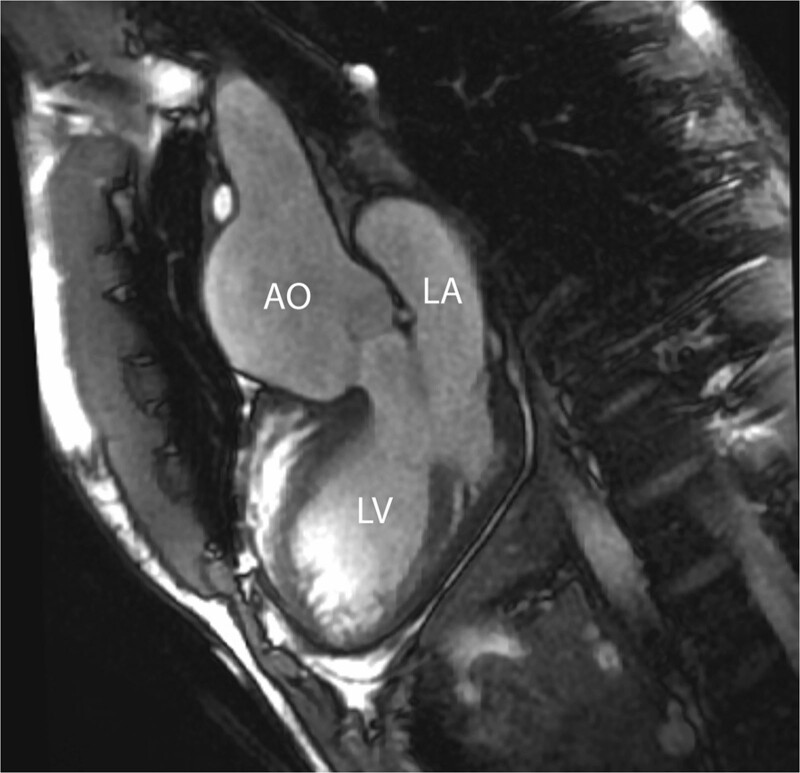
Aortic root dilation. bSSFP cine image in a vertical long-axis view through the inlet and outlet of the LV demonstrates a significant dilation of the aortic root. Ao indicates aorta; LA, left atrium; and LV, left ventricle.

Even though reoperation on the neoaortic valve in the currently studied adult patients was rarely necessary, the potential progression of both neoaortic root dilatation and valve regurgitation should have accurate imaging follow up.^197^ CMR is the ideal modality as it provides both diameters of the neoaortic root measured in different planes and reproducible quantification of the neoaortic valve regurgitation (Figure 14).

### Summary of Recommendations

- Prior to surgery, CMR is useful in evaluating anatomy and physiology required for medical or surgical management in patients with TGA which is not delineated by echocardiography (Class I, Level of evidence C).
- A comprehensive CMR examination should be performed during routine follow-up of patients who received an ASO and is complimentary to echocardiography (Class I, Level of evidence B)
- CMR is beneficial for quantification of biventricular volumes and function in TGA after the ASO (Class I, Level of evidence B).
- CMR is beneficial for visualization of the coronary arteries in TGA after the ASO (Class I, Level of evidence B).
- CMR is recommended for evaluation of the main PA and branch PA stenosis with assessment of differential pulmonary flow (Class I, Level of evidence B)
- CMR is recommended for measure of neoaortic root enlargement and quantification of neoaortic valve regurgitation (Class I, Level of evidence B)
- Vasodilator stress perfusion CMR imaging is useful in symptomatic patients to test for ischemia (Class I, Level of Evidence B) and may be considered as an initial, non-invasive screening test for myocardial perfusion defects and therefore detection of potential coronary artery obstruction. (Class IIB, Level of evidence B)
- In the case of suspected myocardial perfusion defects, CMR may be considered for visualization of coronary ostial stenosis (Class IIB, Level of evidence B)
- CMR is useful in screening for myocardial scarring with LGE (viability imaging) or in confirming the diagnosis in cases of symptomatic individuals, given manipulation of the coronary arteries in this lesion (Class I, level of evidence C).

## Pulmonary Venous Anomalies

### Background

**Figure 15.**
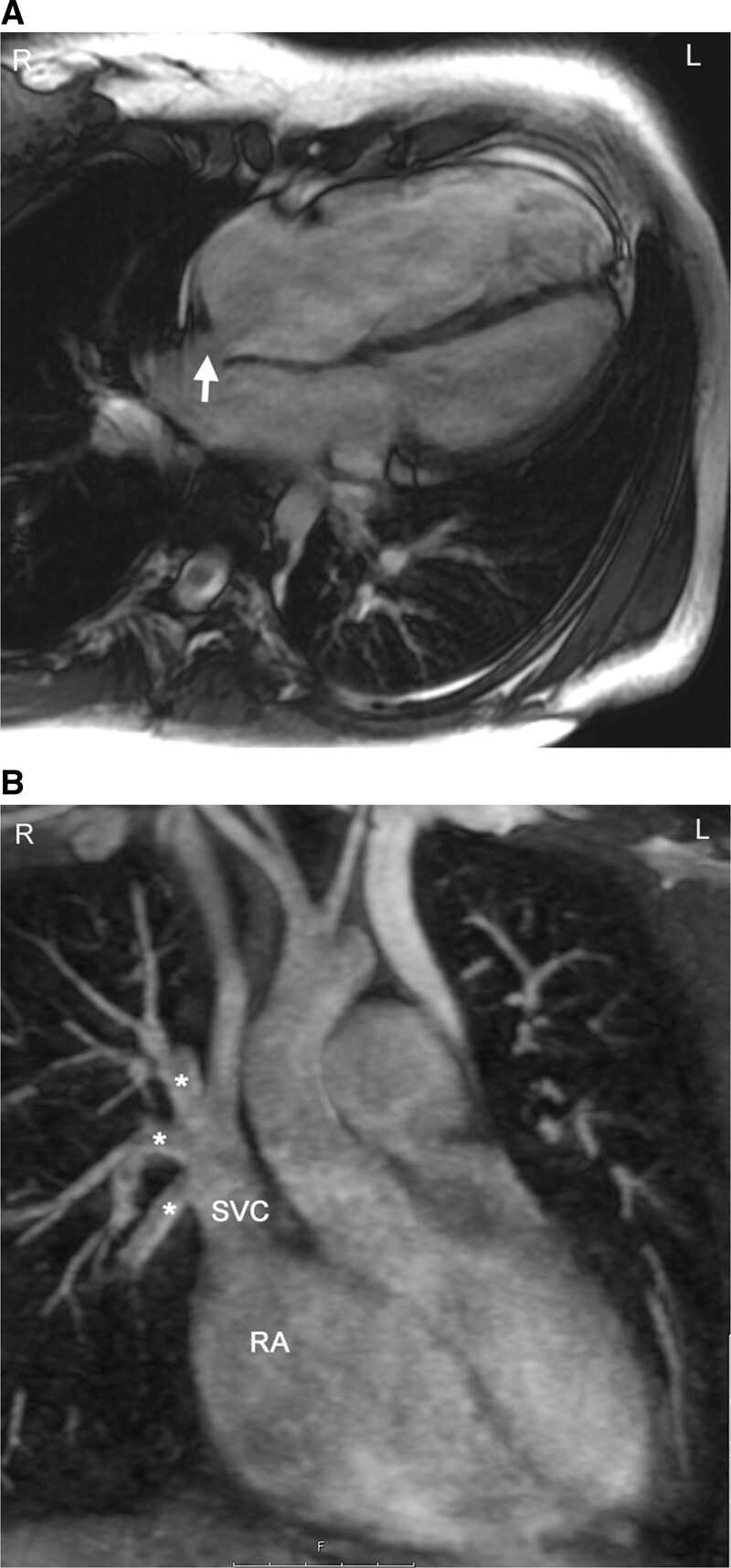
Sinus venosus atrial septal defect with partial anomalous venous connection. **A**, bSSFP cine image in a horizontal long axis view showing a dilation of the right atrium and right ventricle. The arrow indicates the septal defect of sinus venosus type. **B**, The right upper and at least one branch of the right middle pulmonary vein (stars) are connected to the superior vena cava (SVC). This is a reconstructed maximum intensity projection image from contrast-enhanced cardiac magnetic resonance angiography. RA indicates right atrium.

**Figure 16.**
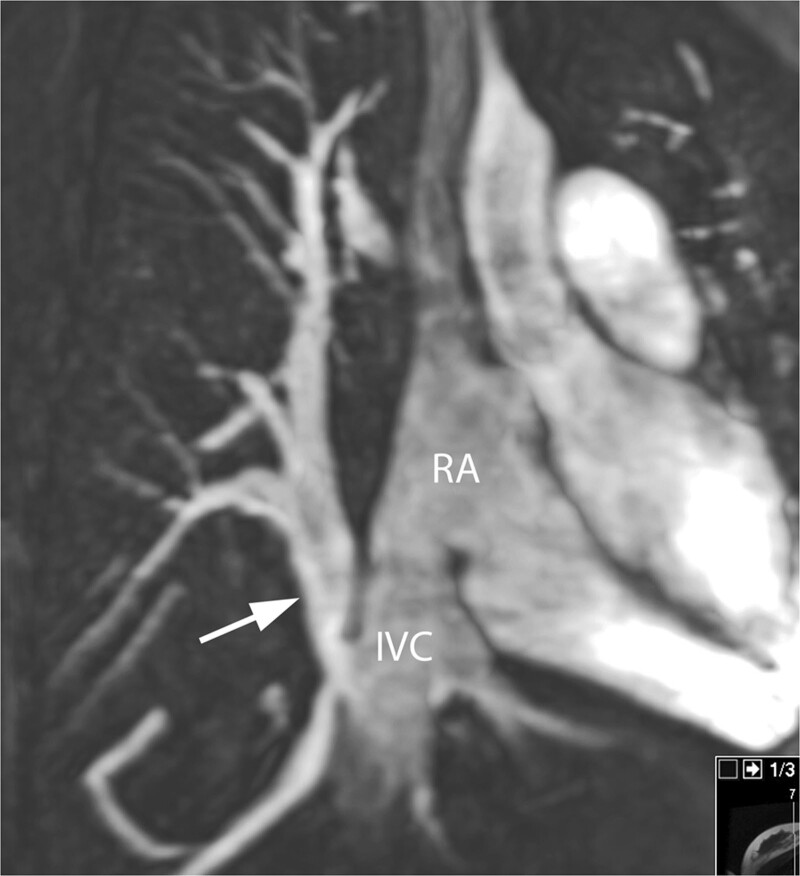
Scimitar syndrome. All venous drainage from the right lung is connected (arrow) to the inferior vena cava (IVC) at the entrance in the right atrium (RA). Reconstructed maximum intensity projection image from contrast-enhanced CMR angiography.

Anomalies of the pulmonary veins (PVs) can be congenital or acquired after an intervention or during the progression of a disease. Congenital PV lesions are rare and occur with a prevalence of 0.6–1.2 / 10 000 livebirths.^11,198^ Partial anomalous PV connection (PAPVC) is the most frequent observed lesion and can occur in isolation but more frequently in association with an ASD (specifically a sinus venosus ASD) (Figure 15). In presence of a sinus venous ASD of the SVC type, a right upper PV connecting to the SVC is common whereas in a sinus venosus ASD of the IVC type, the right lower PV will connect to the inferior margin of the right atrium. Another condition associated with PAPVC is Turner Syndrome in which typically the left upper PV connects to the innominate vein.^199^ In Scimitar syndrome, usually all right PVs connect anomalously to the IVC^200,201^ which may occur below the diaphragm (Figure 16).

**Figure 17.**
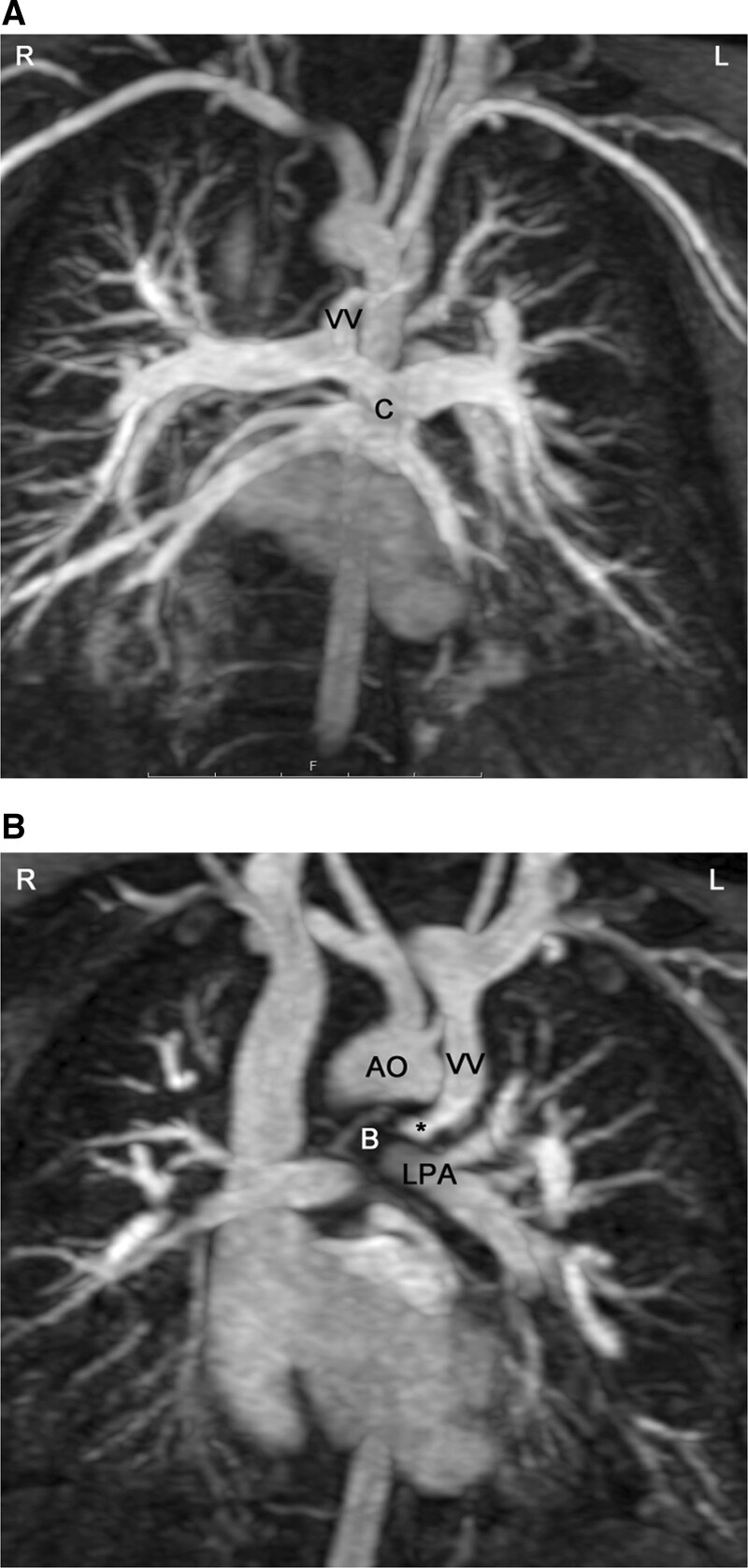
Total anomalous pulmonary venous connection of supracardiac type. **A**, All 4 pulmonary veins are connected to a collector (c), which is draining to a vertical vein (vv). **B**, Along its course to the innominate vein, the vertical vein (vv) passes between the left main bronchus (B) and the left pulmonary artery (LPA). This represents an anatomic vice (*) which may cause obstruction of flow. Ao indicates aorta.

**Figure 18.**
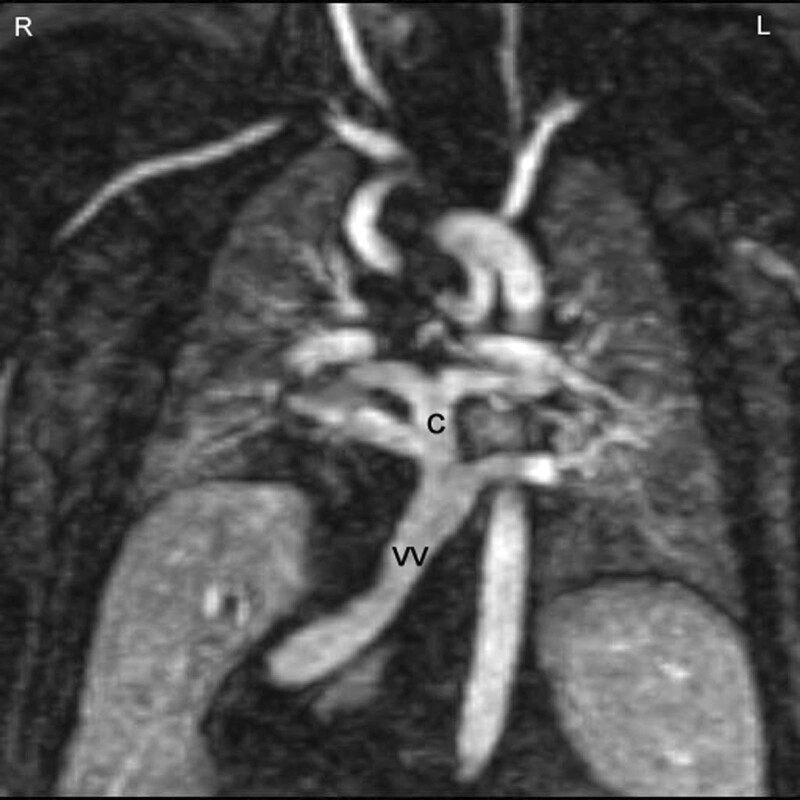
Total anomalous pulmonary venous connection (TAPVC) of infracardiac type. In infracardiac type the collector (c) is draining to a vertical vein connected to the liver portal system. This contrast-enhanced CMR angiography was acquired in a patient with heterotaxy syndrome, typically associated with TAPVC.

Total anomalous pulmonary venous connection (TAPVC) occurs in four different formations defined by the location of the connection of the PVs to the right-sided circulation. In order of prevalence, the sites of connection are: supracardiac (Figure 17), infradiaphragmatic (Figure 18), cardiac, and mixed type. TAPVC can occur in isolation or in association with complex CHD such as heterotaxy syndrome.^202–204^ As variations of the course of the PVs are not infrequent, accurate imaging of each PV is mandatory prior to surgery. Moreover, obstruction in the PV pathway is not rare and can be caused by (a) stenosis of each PV, (b) stenosis at the site of connection, (c) extrinsic compression of the connecting channel, (d) compression of the vertical vein in between the left bronchus and the left PA (supracardiac type) (Figure 17), (e) in the infradiaphragmatic type, compression in the small esophageal hiatus, in the ductus venosus or in the capillary system of the liver (Figure 18) or other solid parenchymal organ.^205^ TAPVC with obstruction of the PV drainage causes severe symptoms of pulmonary congestion in the first days of life and requires immediate surgical or catheter based relief. Unobstructed TAPVC usually leads to significant left to right shunt and heart failure in the first weeks of life.

**Figure 19.**
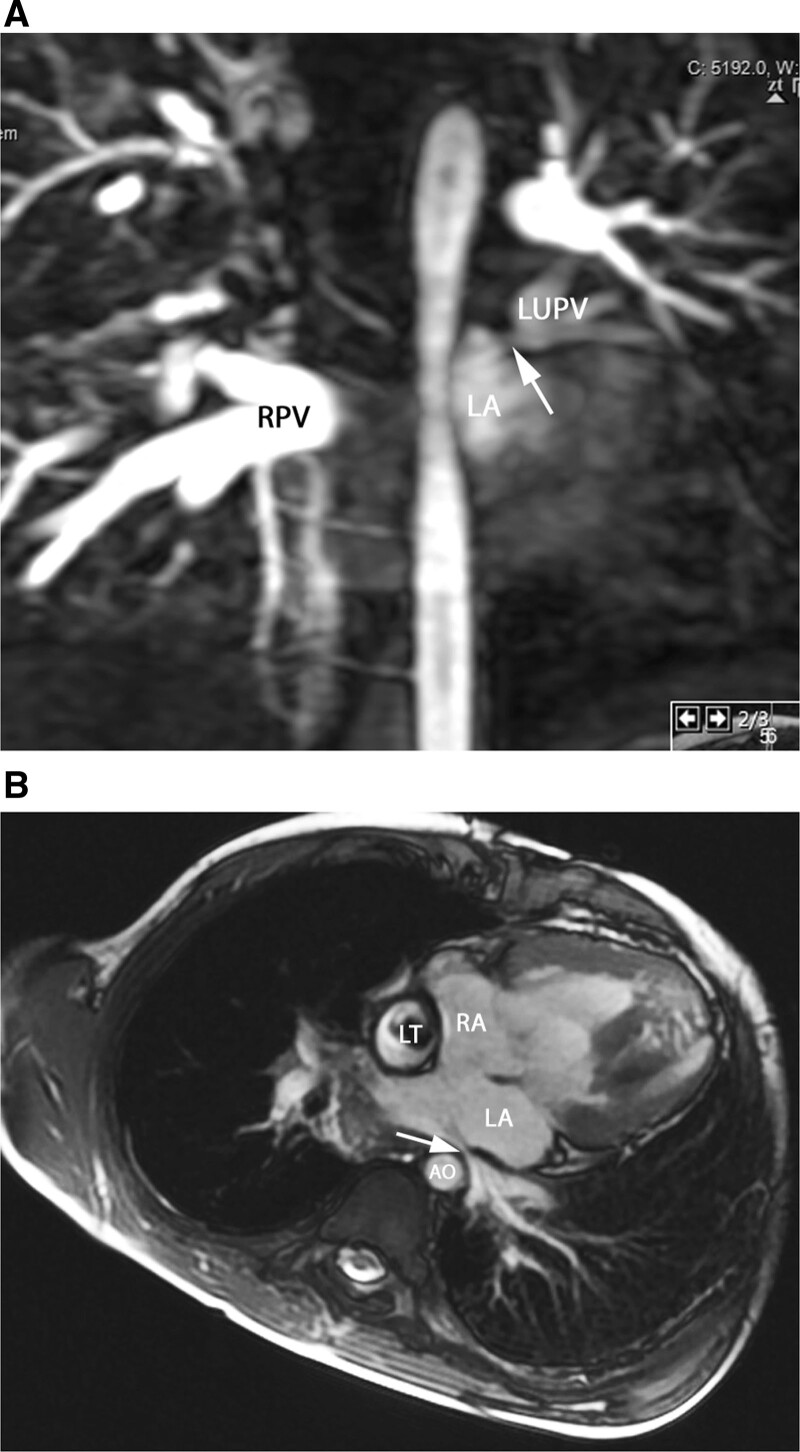
Pulmonary vein stenosis. **A**, Contrast-enhanced CMR angiography depict an intrinsic stenosis (arrow) of the left upper pulmonary vein (LUPV). Note the difference of contrast between the left and the right pulmonary veins (RPV), as well as the large diameter of the right pulmonary veins, probably caused by flow redistribution between the two lungs. **B**, Pulmonary vein (PV) stenosis in the Fontan patient is demonstrated in this bSSFP cine image in an axial view; external compression of the left lower pulmonary vein visualized which is impinged (arrow) between the left atrium and the descending aorta (AO) and spine. LA indicates left atrium; LT, lateral wall tunnel; and RA, right atrium.

Finally, congenital stenosis of one or more PVs can occur, a progressive disease which leads to a dismal outcome (Figure 19).^206^ This can occur with or without associated CHD, and unilateral PV stenosis can lead to flow asymmetry to the lungs (i.e., decreased flow to the lung associated with the PV stenosis). It is not uncommon to have recurrent and/or progressive PV obstruction or even death after surgical repair.^207^

### CMR Indication in PV Anomalies

CMR is nearly always performed after an echocardiography for all PV anomalies (Figures 15, 16, 17, 18, 19). In presence of an ASD, particularly of the sinus venosus type (Figure 15), CMR is indicated for ruling out a PAPVC whenever TTE is not conclusive.^208,209^ Greater than 4 PVs may be present and therefore, echocardiography delineation of only 4 PVs without searching for others in lesions associated with PAPVC is not sufficient and CMR is mandatory. In patients with limited acoustic windows, CMR is indicated to delineate other PVs even if 4 are visualized on echocardiography. Besides providing clear information on the exact location of PV connection and drainage required for proper planning of all surgical corrections such as the Warden operation,^210,211^ CMR is useful to assess size and function of the dilated right heart, assessing the contribution of the anomalous vein(s) to the left to right shunt and to measure Qp/Qs (Figure 15). In case of isolated PAPVC, quantification of Qp/Qs and right heart dilation is a major determinant of indication for surgical repair.

CMR is the modality of choice for evaluation of Scimitar syndrome,^212,213^ demonstrating not only the anomalous drainage of all right PVs into the IVC (Figure 16) with the typical shape of an Arabic sword (scimitar) but also the associated anomalies including dextrocardia, right lung hypoplasia, horseshoe lung, aberrant systemic arterial blood supply to the right lower lung. In addition, in TAPVC, CMR is an important tool for visualization of each single pulmonary vein, their exact site of insertion (especially in mixed TAPVC), drainage, and if present, site of obstruction. (Figure 17). Moreover, shunt fraction and blood flow distribution to each lung can be quantified.

In congenital stenosis of one or more PVs, CMR angiography provides exact depiction of the location of stenosis, number of veins affected and can be repeated during follow up (Figure 19). PV stenosis is also a possible complication after surgical TAPVC repair^214,215^ and if all PV are not affected, pulmonary flow redistributes among the different lung segments. Recognition of the severity of obstruction can be difficult by echocardiography as severely diminished flow may not induce turbulence seen by color flow Doppler at the site of stenosis; on the other hand, turbulence may be visualized at a PV vein which is normal size but has increased flow across it. For congenital PV stenosis and after surgical repair for TAPVC, CMR can clearly depict PV stenosis^216,217^ and delineating not only peak velocities in them (with PC-CMR) but also flows in each PV and PA.

Flows in the PVs can be assessed quantitatively^218^ and qualitatively.^219^ The normal PV flow curve consists of 2 forward waves during systole and early diastole as well as a short wave of reverse flow during late diastole at atrial contraction. This normal flow profile can be altered in several conditions or if atrial compliance is disturbed. In presence of unilateral PV stenosis, a redistribution of flow occurs within the lung and can be assessed by measuring the flow in the pulmonary arteries by CMR; this is correlated with particular changes in flow profile.^220,221^ Flow profiles are also affected directly within the PVs; proximal to a focal stenosis, flow loses its triphasic profile similarly as observed by using Doppler echocardiography.^219^ On the other hand, peak PV flow velocities > 100 cm/s indicate significant obstruction.^217^

In complex CHD, particularly heterotaxy syndrome, PV anomalies are frequent and occur in a wide anatomic variety.^204^ Due to anatomic complexity, echocardiography is often insufficient to describe all diagnostic features required for surgical and medical management. In these lesions, CMR has an important role for surgical planning or staged palliation^222,223^ especially in SV patients. The PV may become impinged between the dilated heart and the spine or the descending aorta (Figure 19). PV occlusion may increase the overall resistance to pulmonary flow which has a negative impact on the Fontan circulation and ultimately clinical outcome.

In general, in comparison to other modalities, cross sectional imaging is superior to echocardiography or conventional angiography due to 3D data acquisition which enables a targeted multiplane reformatting and therefore, visualization of each single PV without superimposition of other vascular structures.^224,225^ CT has the same ability to delineate anatomy. CMR has been validated against lung perfusion scintigraphy for measurement of differential lung perfusion and has been shown to be a similarly accurate and robust modality.^187,226^ In patients with CHD, CMR flow has been shown to be even more accurate (especially in the presence of systemic to pulmonary collaterals) and to overcome some pitfalls associated with scintigraphy.^188^

### Summary of Recommendations

- In patients with PV stenosis or suspected anomalous PV connection, whether PAPVC or TAPVC, CMR should be performed for anatomic evaluation whenever echocardiography is insufficient (Class I, Level of evidence B).
- CMR is useful to understand the hemodynamics of PV anomalies such as calculating any shunt (Qp/Qs) caused by anomalous PV connection and associated intracardiac lesions as an indication for surgical repair (Class I, Level of evidence B) as well as quantifying differential lung perfusion with flow redistribution (Class I, Level of evidence B).
- CMR examination should be performed for assessing PV anatomy in cases with complex CHD when there is a clinical or imaging suspicion of anomalies of PV connection or drainage, particularly heterotaxy syndrome (Class I, Level of evidence B)
- CMR angiography should be performed for surgical planning of repair of PV anomalies (Class I, Level of evidence B)
- It is reasonable to perform at least one CMR examination during follow up after surgical repair for PV anomalies (Class IIA, Level of evidence B).

## Coronary Artery Disease

### Background

Categorically, pediatric coronary artery pathologies can be either congenital or acquired. Acquired lesions can also be sub-categorized as either “disease” based or “surgically” based. Congenital lesions would include those related to anomalous aortic origin of a coronary artery (AAOCA) from an inappropriate sinus (e.g., anomalous origin of the left coronary from the right sinus of Valsalva), anomalous origin from a different vessel such as anomalous origin of the left coronary from the PA, and/or anomalous course of a coronary artery (eg intraseptal) or exit (eg coronary cameral fistulae as seen in pulmonary atresia with intact ventricular septum). “Acquired disease” based lesions would include Kawasaki disease whereas “surgically” based lesions would include alterations of the locations of the coronary ostia or their proximal courses related to corrective surgeries such as the arterial switch operation (Jatene procedure)^227^ or Ross procedure.^228^ Clearly, many of these diseases lend themselves to potentially decrease myocardial perfusion, possibly resulting in ischemia and infarction.

Echocardiography, CT, and CMR are the most commonly used non-invasive imaging modalities for the evaluation of pediatric patients with coronary artery pathologies. Echocardiography is the most easily available with its inherent mobility and high temporal resolution and remains the front-line imaging modality. In many instances, echocardiography is utilized as a screening tool for progression of disease (e.g., Kawasaki’s disease) or may suggest a pathology as an incidental finding (e.g., an echocardiography for evaluation of physical examination findings of a murmur that suggests AAOCA).

Cardiac CT, with state-of-the-art dual-source or volume CT scanners, compared to CMR performed on 1.5 T or 3 T CMR scanners, has slightly higher isotropic spatial resolution (0.5–0.6 mm), faster total examination and scanning time and can, at times, accommodate high heart rates (> 120 bpm) despite a modest temporal resolution for nearly all scanners of 75 ms, although the fastest scanner can obtain a resolution of 66 ms. The drawbacks are that CT requires ionizing radiation and rapid bolus intravenous injection of iodinated contrast agent along with possibly administering medication to slow the heart rate (e.g. propranolol). Cardiac CT typically is limited to morphologic imaging due to radiation exposure concern.

**Figure 20.**
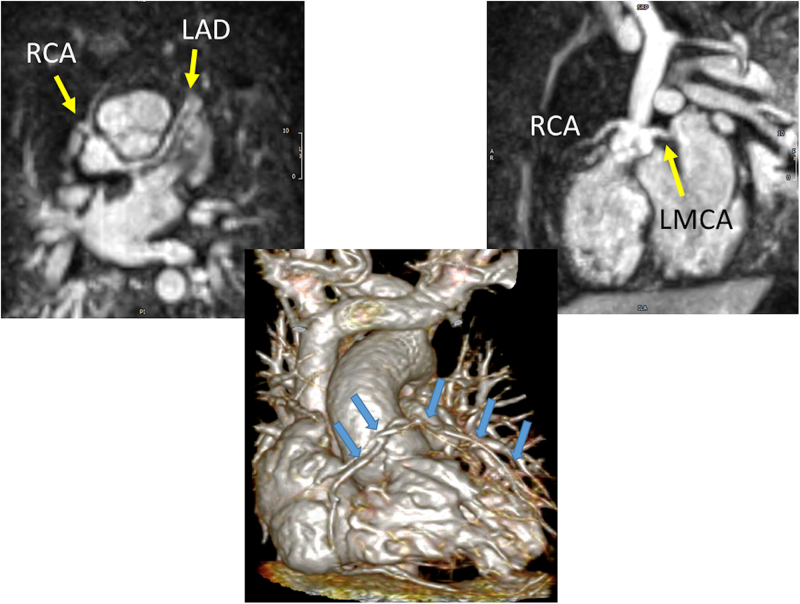
Coronary artery imaging in infants utilizing ferumoxytol. The top two panels demonstrated normal origins and courses from a 2 day old with Taussig Bing anomaly and hypoplastic aortic root and ascending aorta in the off-axis axial (left) and sagittal views (right). The lower image is a 3D reconstruction from a 5 day old with tetralogy of Fallot and pulmonary atresia demonstrating a single right coronary artery (RCA); arrows outline the RCA and left main (LMCA) and anterior descending coronary arteries (LAD).

CMR is generally utilized to confirm the diagnosis by echocardiography as well as to allow for visualization of longer segments with whole-heart coverage^229^ in addition to assessing myocardial function (both regional and global), perfusion^177^ and infarction (ie viability imaging).^50,172^ Further, CMR adds anatomic information as well in the same patient such as those with TGA after ASO.^172^ CMR can obtain in-plane resolution of 0.5–0.6 mm^230^ at 3 T in children and can usually obtain 1–1.2 mm isotropic resolution at 1.5 T. CMR can also obtain coronary images without the need for contrast in the pediatric age range^165,231,232^ in multiple different formats (eg bright blood or dark blood)^233^ although contrast can enhance the imaging.^234^ Newer techniques,^235,236^ currently utilized in several pediatric centers, take advantage of the significant increase signal afforded by the iron-particle blood pool contrast agent, ferumoxytol, and have shown very promising results with sub-mm isotropic whole-heart coverage even in infants with high heart rates (Figure 20) (0.6-0.8 mm in-plane resolution at 1.5 T with slightly lower resolution in the z axis).

### Indications for CMR to Assess Coronary Arteries

#### Congenital

**Figure 21.**
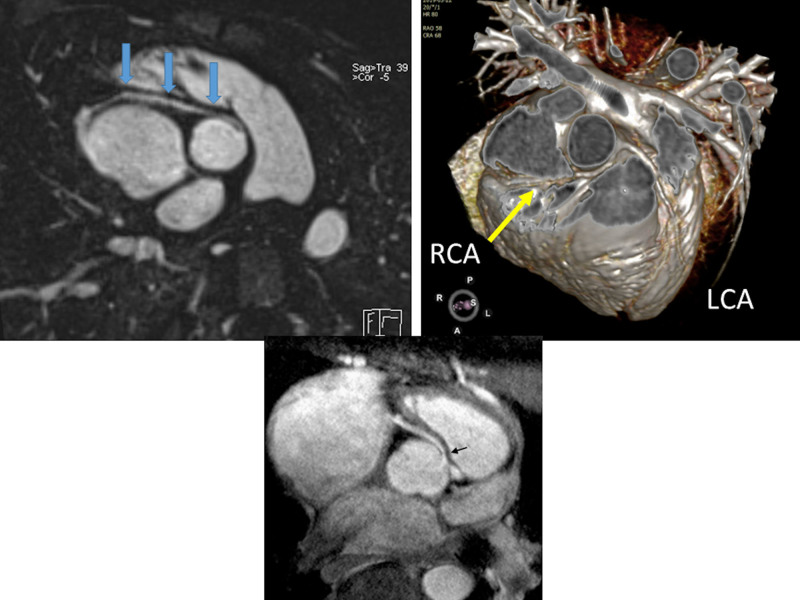
Anomalous origin of the right coronary artery (RCA) from the opposite sinus in an off axis axial view (top left with arrows demonstrating the RCA course) and with a 3D reconstruction (top right). Lower panel is high resolution from a 3 T CMR scanner demonstrating the same with arrow showing origin of an intramural course.

**Figure 22.**
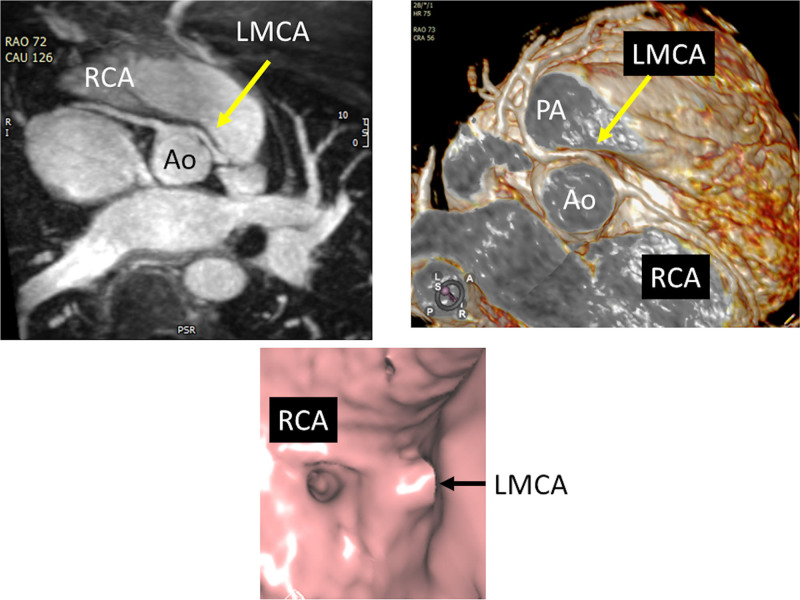
Anomalous origin of the left main coronary artery (LMCA) from the right sinus in an off axis axial view (top left) and with a 3D reconstruction (top right). Lower panel is an endoscopic view of the same patient demonstrating the orifice origins and shapes; note the round right coronary artery (RCA) os and the oval LMCA os.

Following screening echocardiography for suspicion or diagnosis of AAOCA, CMR should be used to confirm the presence of AAOCA and further characterize the location and shape of the ostium and proximal and mid-segment course of the anomalous coronary artery (Figures 21, 22). It should also be used to assess biventricular function both globally as well as regionally to assess regional wall motion abnormalities which may be due to AAOCA. Finally, viability can be performed on a routine basis to determine if there is any discrete LGE due to myocardial infarction. Vasodilator stress perfusion CMR should be reserved for special cases.

In a prospective study of 50 patients (age range, 18 days to 18 years), Hussain et al.^229^showed that whole-heart coronary artery CMR has a success rate of 94% for the detection of coronary origins. Brothers et al. demonstrated prospectively in a small group of patients the multifaceted utility of CMR by characterizing stenosis, perfusion and fibrosis both prior to and after surgery in children with AAOCA.^167^ The latest techniques using ferumoxytol overcomes previous spatial limitations (Figure 20) even in small infants. Detailed depiction of the anomalous coronary artery and its proximal course can also be achieved with high in-plane sub-mm spatial resolution coronary CMR imaging with a targeted approach and 3D endovascular view of the morphology of the ostium can be performed (Figure 22).^237^ Most recently, in one institution, a large cohort of 5,169 asymptomatic volunteers (11—18 years of age) were screened with CMR for cardiomyopathy and anomalous coronary artery origin from the opposite sinus with an intramural segment (ACAOS-IM). There were 23 such cases (6 left-ACAOS-IM and 17 right-ACAOS-IM)^238,239^ establishing a prevalence of 0.4%.

**Figure 23.**
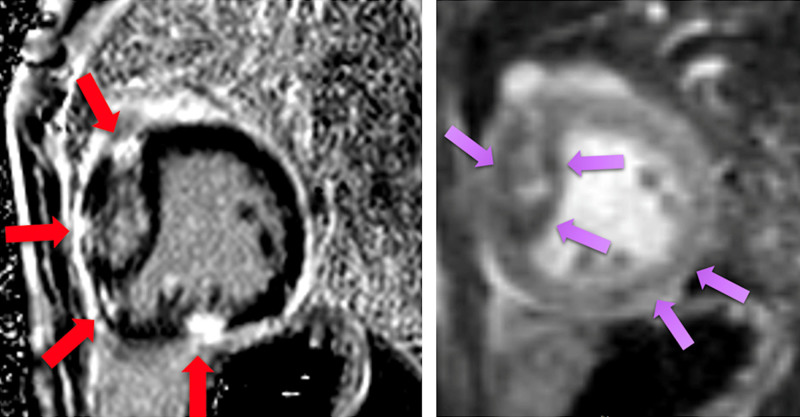
A 3 month old with pulmonary atresia with intact ventricular septum with coronary cameral fistulae and a RV dependent coronary circulation. The right panel is an adenosine stress perfusion CMR demonstrating perfusion defects (arrows) while the left panel demonstrates myocardial scarring in that same patient (arrows).

Other important anatomic findings of the coronary arteries can be detected by CMR such as a conal branch coursing anteriorly across the RVOT and/or position of left main coronary and proximal left anterior descending (LAD) artery with respect to RVOT, significant in patients with TOF.^240^ In a prospective study of whole-heart coronary CMR in 100 patients (age 2 months–11 years; median 3 years), of the 58 patients who underwent surgery, all CMR coronary artery findings were confirmed including 4 cases of coronary anomalies.^165^ In addition, coronary anomalies of “course” such as an intraseptal or retroaortic course, are important to delineate and have been demonstrated by CMR.^237^ Finally, anomalies of exit such as those with pulmonary atresia with intact ventricular septum with coronary cameral fistulae or a RV dependent coronary circulation can be delineated by CMR, especially important because of the sequelae of myocardial infarction (Figure 23).

#### Acquired

**Figure 24.**
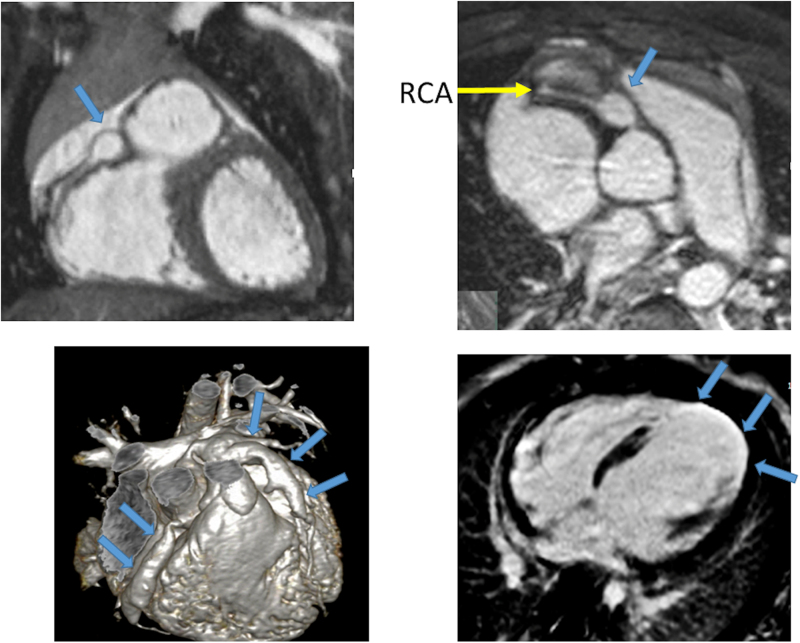
Kawasaki’s disease. Top panels demonstrate a discrete right coronary artery (RCA) aneurysm (arrow) in a 3 year old. The bottom left panel is from a 2 year with diffuse aneurysms of both the right and left coronary systems (arrows); clots were found in the left coronary systems the resultant infarct and rounding of the left ventricular apex (arrows) is seen from the 4-chamber viability imaging (right).

CMR can be used to assess for the morphology including size, shape, and location of coronary aneurysms in diseases such as Kawasaki’s disease (Figure 24). Similar to other CHD, it should also be used to assess global and regional ventricular performance, viability and perfusion (perfusion in select cases).

Mavrogeni et al.^241^ has shown in a prospective study of 16 patients (age range 3–8 years) that coronary CMR correlated completely with invasive cath for size and location of aneurysms. There were no cases of stenoses for comparison. Suzuki et al.^242^ and in related work by Takemura et al.^242^ showed in a retrospective study with a larger cohort of 106 Kawasaki disease patients (median age 13 years, range: 4 months–33 years) and a smaller cohort of 35 consecutive pediatric KD patients (under 6 years of age) that imaging of coronary arteries in pediatric patients with Kawasaki disease can routinely be performed with a 96% success rate. High sub-millimeter spatial resolution imaging that is used for AAOCA has been successful in young patients with Kawasaki disease.^243,244^ In addition, CMR should also assess for global and regional ventricular dysfunction, coronary perfusion, and myocardial scarring/LGE during the convalescence and follow up of the Kawasaki disease patients.^245,246^ Advanced imaging of characterization of the coronary vessel wall has also been shown to be possible.^246^

CMR can be used to characterize the anatomy of the coronary arteries following surgery in which the position and/or origin of the native coronary arteries have been altered such as after the Ross procedure or TGA after ASO (Figure 10). Taylor et al. reported in a prospective study of 50 asymptomatic pediatric TGA patients after ASO (age range 6–16 years) a comprehensive CMR examination including coronary artery CMR, cine imaging for ventricular function, and myocardial characterization for scarring.^172^ In 100 patients after ASO, Rodriguez et al. studied 100 whole heart 3D CMRs and found coronary artery stenosis in nearly 11%.^166^

Imaging coronary artery walls for vasculopathy in transplant patients^247^ including those in the pediatric age range^248^ and in other diseases such as Takayasu’s arteritis^249^ is an emerging application of CMR coronary imaging but should be considered experimental at this time.

### Summary of Recommendations

- For patients with suspected AAOCA or other congenital anomalies of origin, course or exit, CMR is recommended to depict the origin and detailed anatomy of the vessels for both diagnosis and pre-operative planning (Class I, Level of evidence B).
- For patients with Kawasaki disease or other acquired “diseased” based coronary pathology, CMR is recommended to accurately depict the size, shape, and location of coronary aneurysms (Class I, Level of evidence B).
- For patients with acquired “surgically” based coronary pathology such as TGA after ASO or Ross procedure, CMR is recommended to evaluate the post-operative coronary anatomy as part of a clinically indicated comprehensive CMR examination. (Class I, Level of evidence B)
- CMR should be utilized to assess the secondary effects of congenital coronary anomalies or acquired pathology such as effects on myocardial function (e.g., regional wall motion abnormalities, end-diastolic volume, ejection fraction), perfusion and infarction (Class I, Level of evidence B) both prior to and after repair (if surgery) or in followup.

## Coarctation of the Aorta and Bicuspid Aortic Valve

### Background

**Figure 25.**
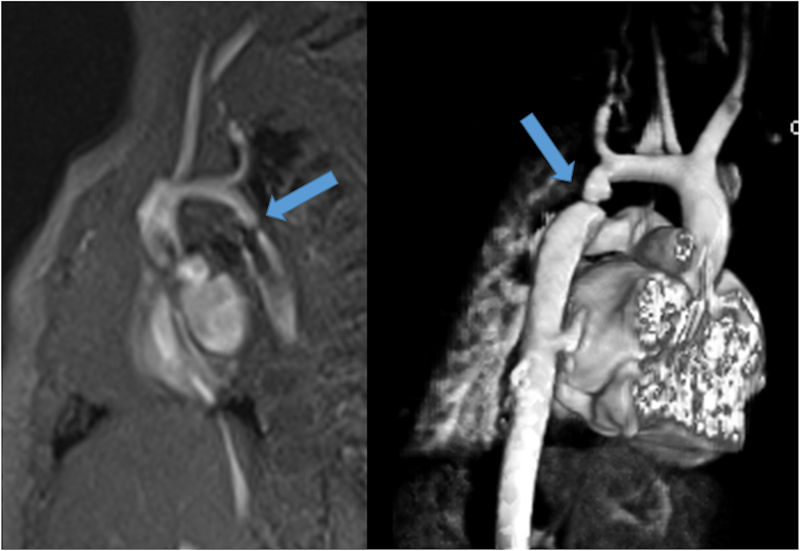
Coarctation of the aorta in the juxtaductal region in a 4 month old utilizing unbalanced gradient echo cine imaging (left) and 3D reconstruction (right). Note the turbulent jet on cine. Arrows point to the coarctation.

**Figure 26.**
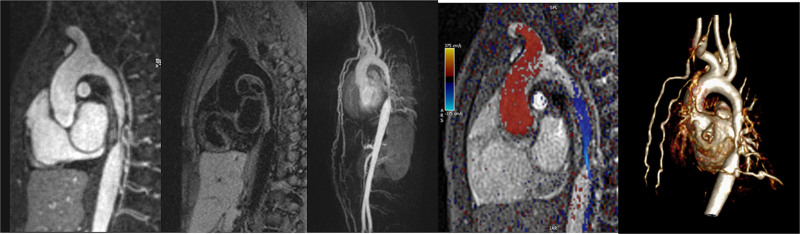
Coarctation of the aorta in the mid-thorax. This figure demonstrates the multiple ways a coarctation can be imaged by CMR. From left to right, there is 2D bright blood gadolinium enhanced imaging, a multiplanar reformat of a 3D dark blood sequence, a maximum intensity projection 3D image, inplane velocity mapping with color coding of the flow through the coarctation and a 3D volume rendered display. Note the collaterals on the maximum intensity projection and 3D volume rendered display. Color coding of the inplane velocity map is red cephalad and blue caudad.

**Figure 27.**
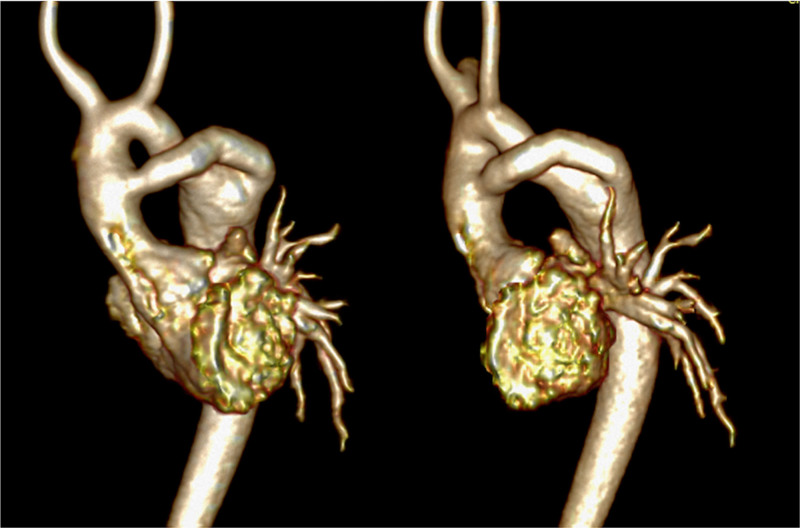
Volume rendered image of an ascending to descending aortic conduit from anterior (left) and lateral (right) views to repair a coarctation utilizing gadolinium enhanced 3D imaging by CMR.

Coarctation of the aorta is the most common left sided obstructive heart lesion with a mean incidence of 409 per million live births^11^ and a prevalence of ~ 7% of patients with CHD.^198^ Coarctation is a discrete or relatively discrete narrowing of the proximal descending thoracic aorta, most commonly located in the juxtaductal region, immediately distal to the left subclavian artery (Figure 25) although it may occur anywhere in the thoracic (Figure 26) and abdominal aorta. It can be simple or complex with arch hypoplasia and tortuosity and associated with other significant intracardiac CHD such as HLHS, TGA or truncus arteriosus. Bicuspid aortic valve (BAV) is frequently identified in patients with coarctation with studies reporting an incidence of > 50% and up to 85% (Figure 27).^250^ Coarctation is part of a spectrum of aortopathies; genetic aortopathies are addressed elsewhere in this manuscript.

If not detected prenatally or with pulse oximetry screening, neonates with coarctation of the aorta may present in clinical distress with significant metabolic acidosis and respiratory failure at the time of ductal closure.^251^ The lesion may be ductal dependent, requiring prostaglandin infusion and other supportive measures for resuscitation and survival of the patient until more definitive treatment can be undertaken. Accurate detailed diagnosis is essential prior to any intervention. Echocardiography is the first line imaging modality and may be all that is needed in infants with simple coarctation or associated intracardiac defects. If portions of the aorta proximal or distal to the coarctation or branching vessels are not well visualized, CMR can provide full anatomical details and may be superior to echocardiography.^252^

Coarctation may also present later in childhood or early adulthood, usually in the setting of referral for hypertension or heart murmur and diminished lower extremity pulses. Depending on the age of the patient, chest X-ray may demonstrate rib notching such as when significant large collateral vessels may be present. When a native coarctation of the aorta is first diagnosed in an older child or adult by echocardiography, CMR is then used to define the anatomical and hemodynamic severity in preparation for treatment by surgery or in the cardiac cath lab. Echocardiography may demonstrate the unusual arch anatomy and abnormal Doppler flow patterns through the obstruction, but due to limited acoustic windows, may not allow for full visualization of the coarctation or its proximal and distal segments, aortic arch branching abnormalities, or the presence and magnitude of associated collateral vessels which are all important pieces of clinical information to aid in determining the appropriate method of intervention.

**Figure 28.**
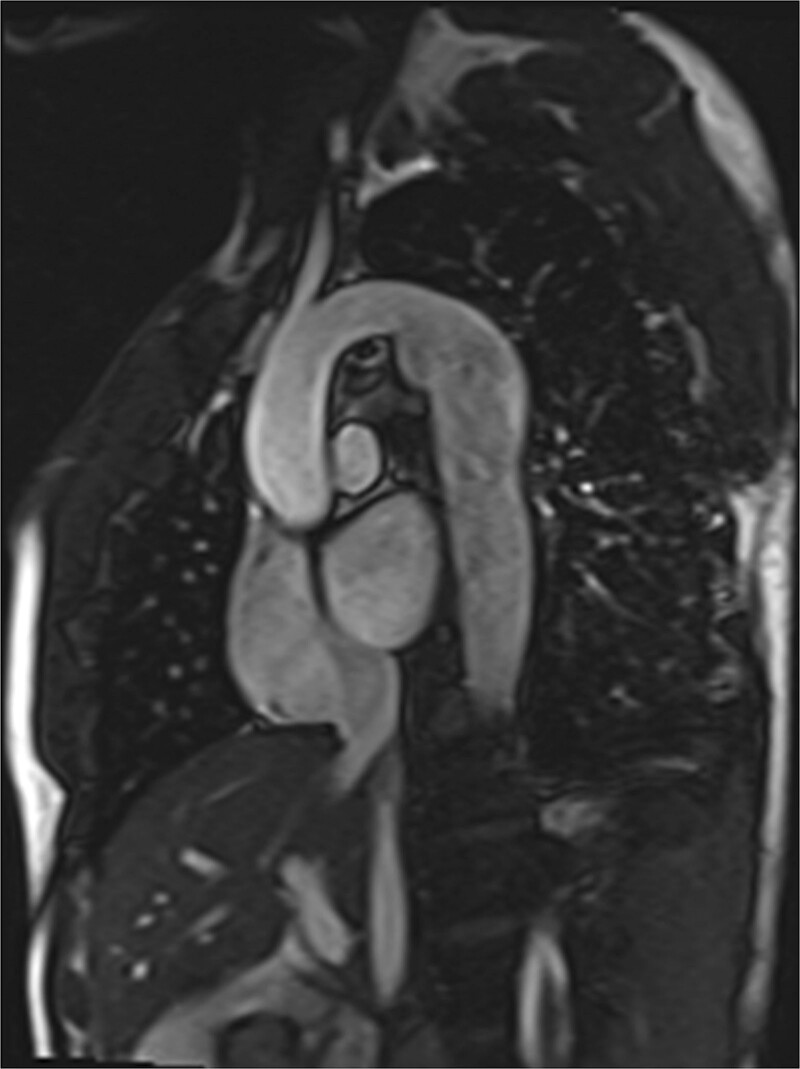
Candy cane view of a 12 year old after subclavian flap angioplasty repair of coarctation of the aorta with a moderate sized aneurysm formation.

In infancy and early childhood, coarctation of the aorta is most commonly repaired surgically with methods including subclavian flap repair, patch aortoplasty and simple or extended end-to-end anastomosis. In some situations, coarctation may be initially treated with balloon angioplasty with or without placement of a stent. In more unusual complex anatomical situations, bypassing the severe arch obstruction with a LV apical or ascending to descending aorta conduit may be rarely undertaken (Figure 27). Regardless of method of initial repair, long-term complications that require monitoring include residual coarctation, recoarctation, associated arch hypoplasia or tortuosity, aneurysm formation near the site of repair (Figure 28), dissection, hypertension and endocarditis. Abnormalities of the aorta will often impact LV function. Long-standing hypertension leads to LV hypertrophy and ventricular dysfunction, as well as increased incidence of coronary heart disease and multiple other sequelae eventually leading to premature morbidity and mortality.^253,254^

### Indication for CMR

**Figure 29.**
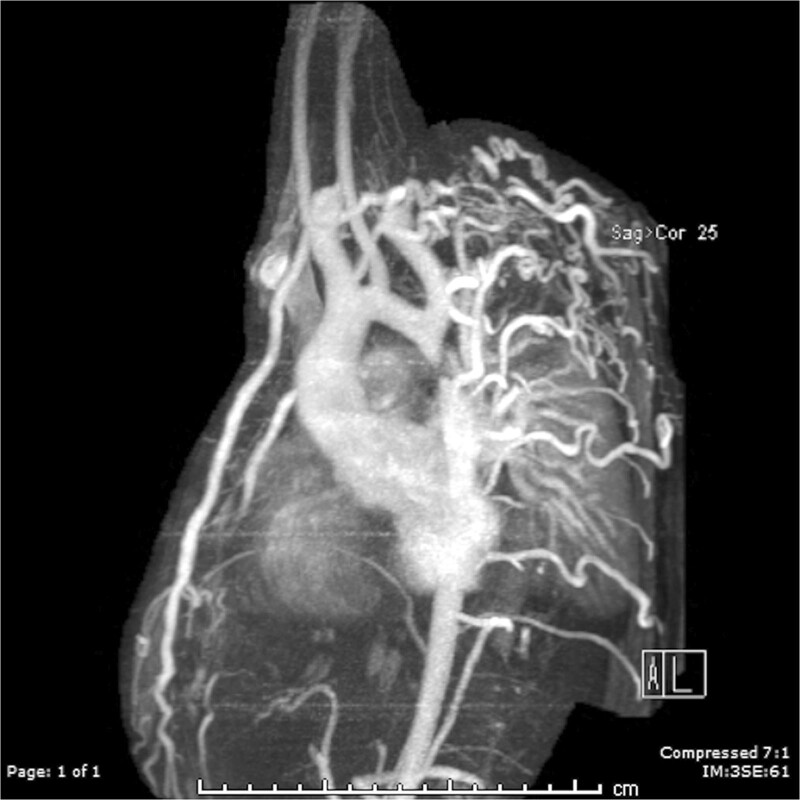
Maximum intensity projection of a patient with coarctation and multiple aortic collaterals. Flow in these collaterals can be quantified (see text).

Important anatomic and hemodynamic data is obtained with CMR having been utilized for many years in the assessment of coarctation of the aorta. Multiple techniques, including non-contrast and contrast CMR sequences are used to provide a complete anatomical 3D analysis (Figure 26). Collateral vessels can be visualized providing flow to the descending aorta distal to the obstruction and CMR can be utilized to quantify this flow both prior to and after intervention (See Colateral flow section of this document) (Figure 29).^255–260^ Anatomic measurements throughout the aorta can be applied and compared to published normative data.^261^ Cine bSSFP provides visualization of the flow disturbance in the narrowed and dilated regions of the aorta and PC-CMR is used to assess flow, estimate gradients (Figure 26)^262^ and quantify collateral flow^255,256,258–260^ (see Qp/Qs and collateral flow section).

CMR is used as preplanning for intervention, as well as continued surveillance of the entire aorta and function of the systemic ventricle.^263^ After surgical coarctation repair, CMR is the preferred imaging modality.^252,264,265^ Monitoring for restenosis is important and visualization of collaterals and measurement of flow volume increase from the coarctation repair site to the diaphragm aorta level is more reliable for assessment of recoarctation than arm-leg blood pressure drop.^259^ Aneurysms may occur at the repair site (Figure 28) and patients with poorly controlled hypertension may be at risk for dissection. The most cost-effective approach to care of patients with coarctation after balloon angioplasty or surgical repair is clinical assessment and CMR in every patient.^266^ In adults, guidelines recommend CMR imaging of coarctation repair for routine follow up at intervals as much as every 1–3 years depending on the patient’s physiologic state^1^ and similar reasoning may be thought of in older children and young adults.

After stent placement, CMR is safe and may be chosen to evaluate areas around the stent and other vascular or intracardiac concerns, including BAV, as well as the effects of residual abnormalities such as LV hypertrophy, cardiac index and collateral flow but is not absolutely dependable for visualizing the interior of the stent although dark blood imaging can visualize patency (but not exact dimensions). With the advent of ferumoxytol, in stent visualization has improved for bright blood imaging but exact dimensions remains still elusive. If visualization of the interior of the stent is needed, CT and invasive angiography may be performed.

Besides collateral flow measurements and cardiac index, 4-dimensional (4D) flow CMR imaging has been utilized to determine flow and visualize 4D flow patterns in coarctation of the aorta both prior to and after repair.^267–269^ There has also been recent work using 4D flow CMR to measure pressure drop in the aorta of patients with coarctation.^270,271^ Although promising, 4D flow is not yet routinely performed in clinical practice across all centers.

Physiologic assessment of the effects of coarctation on the LV can be assessed by CMR as it is the gold standard for this evaluation.^272^ LV mass, ventricular volumes, global and regional ventricular dysfunction should be routinely assessed. LV myocardial strain can be assessed using tissue or feature tracking and can be abnormal with LV hypertrophy and systemic hypertension.^273^ Diffuse fibrosis and increased ECV may be increased in hypertensive heart disease,^94,274^ although not routinely performed clinically. All of these methods for determining LV status will provide important data for making clinical decisions.

### Summary of Recommendations

- At the initial diagnosis of coarctation by echocardiography, CMR is recommended to provide conclusive anatomical and functional details needed prior to treatment or to decide if treatment is needed including anatomy of the coarctation, LV hypertrophy, cardiac index and the presence of a BAV etc. if these are not fully delineated by echocardiography (Class I, level of evidence B).
- CMR is reasonable to assess collateral flow in patients with coarctation of the aorta, especially if it is unclear from other criteria that treatment is needed (Class IIA, level of evidence B).
- After surgical repair of coarctation, CMR is indicated to monitor the status of the aorta and to visualize restenosis or aneurysm formation (Class I, level of evidence B).
- After stent placement, CMR can be useful to provide anatomic and hemodynamic assessment of the aorta surrounding the stent, but not to accurately visualize in-stent stenosis (Class IIA, level of evidence B).
- After coarctation repair, CMR is recommended to assess the aorta and LV function every 1–3 years in children and adolescents, similar to adults, if echocardiography is insufficient or pathology is suspected (Class I, level of evidence B).
- CMR holds the potential to assess LV myocardial strain and diffuse fibrosis/increased ECV in patients with coarctation to assess effects on the LV and may be considered for this purpose (Class IIB, level of evidence B).

## Bicuspid Aortic Valve

### Background

**Figure 30.**
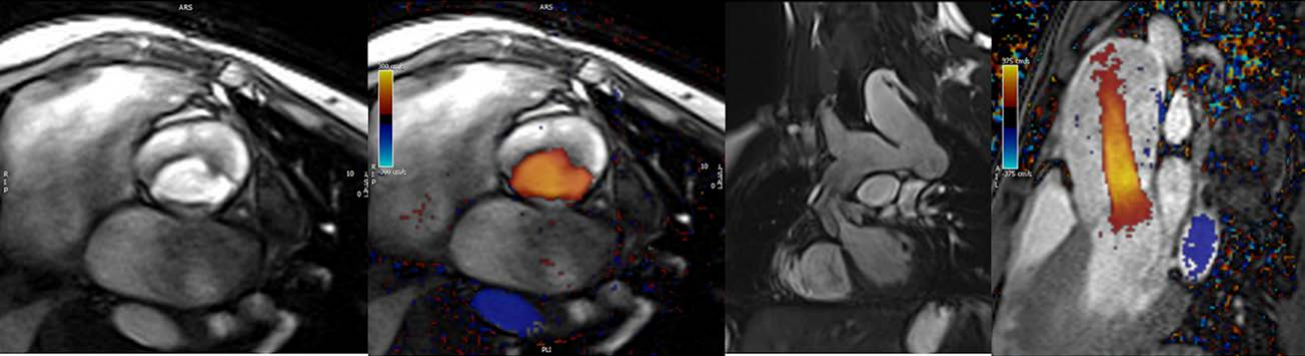
Bicuspid aortic valve with fusion of the right and non-coronary cusps using through plane phase contrast-cardiovascular magnetic resonance (PC-CMR) (left without color and 2nd from left with color). Second from the right panel is an bSSFP image in the LV outflow tract demonstrating the limited excursion of the valve leaflets while the right panel is an inplane phase contrast (PC-CMR demonstrating a peak velocity of 3.5 m/s.

**Figure 31.**
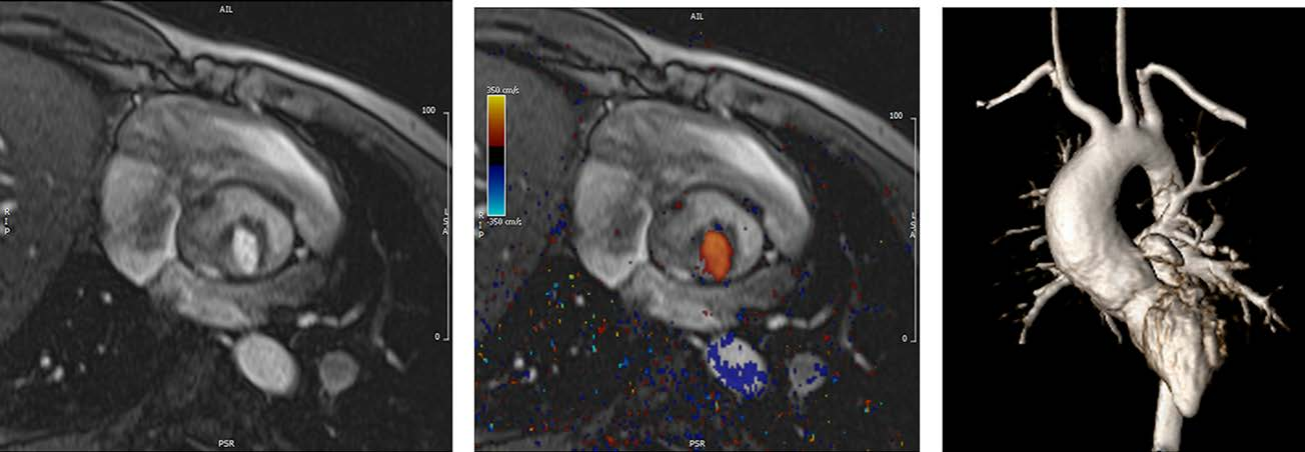
Unicuspid aortic valve with only the left coronary-non coronary commissure open without (left) and with color (middle). Ascending aortic dilation is seen on the right panel

BAV is the most common congenital cardiac abnormality with a prevalence estimated from 0.5 to 2% in the general population and has a 2:1 male to female ratio.^11,275,276^ Some families demonstrate an autosomal dominant inheritance pattern.^277^ Unless associated with significant stenosis or regurgitation or other LV outflow tract lesions, young individuals are often asymptomatic (Figure 30). Critical and moderate to severe aortic stenosis associated with abnormal valve morphology often requires intervention during infancy or early childhood. Later in life, patients may be at risk for associated complications such as ascending aortic dilation with aneurysms (Figure 31) and dissection, aortic stenosis, AR, and endocarditis, all of which could eventually require aortic valve replacement and other aortic surgery. Many cases of BAV diagnosed after childhood may not need intervention until after the fourth decade and beyond.

There are multiple types of valve morphology under the rubric of BAV in children and adolescents.^278^ The most common type is fusion of the right and left coronary commissures (70%) followed by fusion of the right and non-coronary commissures (28%); fusion of the left and non-coronary commissures is the least common. The vast majority of patients with coarctation have fusion of the right and left coronary commissures while fusion of the right and non-coronary commissures is most commonly associated with aortic regurgitation.^278^ Other types of morphology include partial or complete fusion of one or more of the commissures of the coronary cusps with associated cusp asymmetry and complete or incomplete raphe, as well as “true” symmetric BAV cusps without raphe. Some severely malformed valves are labeled “unicommissural” which may or may not be classified under this label (Figure 31).^279,280^

### Indication for CMR in BAV

Historically, TTE, when acoustic windows are adequate, has been reported as highly sensitive and specific for diagnosing BAV.^281^ If not well visualized by echocardiography, CMR can determine aortic valve anatomy (Figures 30, 31). In a more recent analysis comparing pathology specimens with TTE and CMR images, the ability to assess valve morphology was higher with CMR when compared to TTE (96% vs 73%).^282^ Valve morphology has not been definitely associated with severity of disease other than a small subgroup of patients with preferential dilation of the sinuses of Valsalva who have right-left coronary cusp fusion. Flow abnormalities associated with the different types of aortic valve have been characterized, but clinical implications are still being identified.^279^

For aortic valve morphology, CMR planimetry for aortic valve area has been demonstrated to have the best sensitivity and specificity of all non-invasive methods when compared to invasive catheterization and has high reliability and reproducibility.^283–285^ Valve area by CMR can be obtained using velocity time integrals and compares favorably to echocardiography.^286^ Other ways to measure aortic valve area by CMR include Hakki’s formula^287^ and the continuity equation.

CMR can assess hemodynamic effects of associated stenosis and AR from aortic valve disease. This includes LV hypertrophy and dilation as well as functional volumetric analysis, changes in strain or fibrosis and ECV expansion. In those with significant aortic valve dysfunction, CMR is advantageous for serial evaluation of LV volumes and mass. The standard for determining ventricular sizes and volumes is CMR.^272^ Though quantitative evaluation of AR volume can be calculated by the difference between RV and LV volumes (assuming no intracardiac shunting, atrioventricular valve insufficiency or PR), PC-CMR sequences allow for direct measurement of forward and reverse volume used to calculate a regurgitant fraction, as well as peak velocity of the jet in the prescribed plane^286^; checks on the data include subtracting the flow of caval return, main PA flow or the sum of branch PA flowfrom aortic forward flow making CMR more accurate. CMR also allows for assessment of myocardial blood flow with first-pass perfusion at rest and stress, as well as tissue characterization with myocardial LGE for fibrosis and viability. All this information by CMR provides assistance in determining timing for aortic valve intervention or replacement.^3^

CMR allows for evaluation of the ascending aorta (Figure 31) as it relates to BAV disease and should be utilized when the morphology of the aortic root or ascending aorta cannot be viewed well or measured accurately by echocardiography. Once the aorta is dilated, serial yearly evaluation should occur.^288^ Because of its strong association with coarctation of the aorta, patients diagnosed with a BAV should also undergo imaging for coarctation of the aorta.^250^ CMR has also been used to evaluate shear wall stress of the aorta in patients with BAV.^289^ See Genetic Aortopathy section and the 4D flow imaging subsection.

### Summary of Recommendations

- If echocardiography is unable to visualize morphology of a BAV or visualize the aortic root or ascending aorta adequately, CMR is indicated to provide the needed information. (Class I, level of evidence B)
- CMR should be used for serial monitoring in the setting of BAV valve with associated aortic stenosis or AR and to quantify these hemodynamics (Class I, level of evidence B).
- CMR is useful for serial monitoring of the size of the aortic root and ascending aorta in the setting of BAV. (Class I, Level of Evidence B)
- CMR is recommended for serial monitoring of the effects of aortic stenosis and AR on the LV (e.g. LV volumes, EF and mass) (Class I, Level of Evidence B) and to obtain direct measurement of AR volume, peak flow velocity and aortic valve area when determining need for aortic valve/aortic surgery (Class IIA, Level of Evidence B).
- CMR is beneficial to determine lesions associated with BAV such as coarctation of the aorta, interrupted aortic arch other left sided obstructed lesions (Class I, Level of Evidence B).

## Genetic Aortopathy

### Background

Genetic disorders associated with aortopathy include Marfan syndrome (MFS), autosomal dominant Vascular Ehlers-Danlos (EDS), Loeys-Dietz (LDS), aneurysms-osteoarthritis syndromes, Turner syndrome, Noonan Syndrome and non-syndromic familial thoracic aortic aneurysms and dissections (FTAAD). These group of disorders are associated with altered connective tissue composition leading to a combination of cardiovascular, skeletal and often ocular manifestations.

The cardiovascular features include valvular and vascular disease, in particular aortic dilatation with increased risk of dissection, aneurysm, rupture and death. The aortic dilation most often affects the aortic root and ascending aorta but can also affect the more distal aorta including the transverse arch and descending aorta. These disorders have variable effect on other cardiac structures. For example, mitral valve prolapse (MVP) is a feature of MFS and LDS, but not EDS, which in turn has a higher risk of coronary artery dissections than the other aortopathies. Turner syndrome is due to the partial absence of an X chromosome, and phenotypically has short stature and ovarian insufficiency, in addition to aortic dilatation, BAV, PAPVR and aortic coarctation. Noonan Syndrome is considered a renin-angiotensin system (RAS)opathy, a group of disorders which have mutations in the signaling proteins for RAS/mitogen activated protein kinase (MAPK) pathway and results in cardiovascular, facial and lymphatic anomalies. Inheritance and penetrance of all these conditions is variable, with many patients representing new sporadic mutations. The diagnosis of these conditions is most often in infancy, due to the combination of musculoskeletal, facial and cardiovascular anomalies. However, the progressive nature of the associated vasculopathy means that lifelong cardiovascular imaging surveillance is required. Table 6 contains a summary of the genetic aortopathies.

**Table 6.**
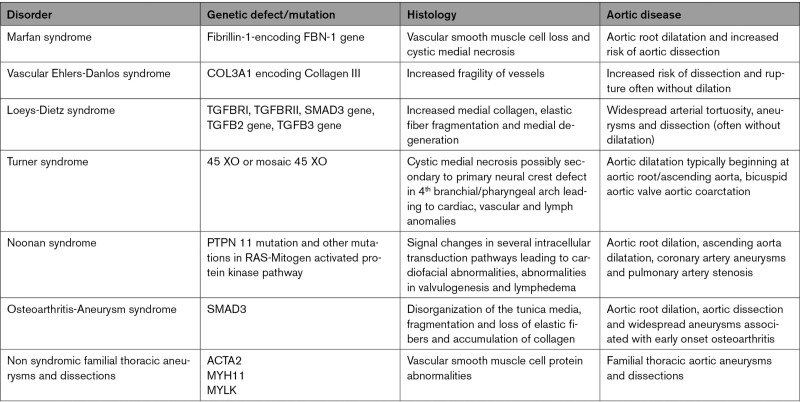
Summary of the Genetic Aortopathies

### Indications for CMR

#### Aortic Size Measurements

A small study comparing reproducibility of aortic measurements at multiple levels in both pediatric BAV and genetic aortopathy patients showed that echocardiography derived measurements were systematically smaller by 5–7% and were less reproducible than CMR measurements, particularly in dilated aortic roots and in the descending aorta.^290^ Most of the genetic aortopathy patients have the potential for complex abnormalities that may affect the entire aorta necessitating comprehensive evaluations. Furthermore, the presence of concomitant skeletal abnormalities renders echocardiography less reliable due to poor acoustic windows. The need for accurate and reproducible aortic size measurements is of utmost importance as aortic size is considered a surrogate for aortic dissection, surveillance recommendations and surgical interventions in genetic aortopathies.^291^ The 2010 published guidelines for diagnosis and management of thoracic aortic diseases including genetic aortopathies with surgical repair recommendations for genetic aortopathies are purely based upon aortic size.^291^

The aortopathy in Turner syndrome is complicated by the presence of short stature necessitating a Turner specific z-score (TSZ) developed for 2D echocardiography.^292^ In patients > 15 years of age, a unique index, the aortic size index (ASI), was proposed (*ASI = maximum aortic diameter/bodysurface area*) and has been shown to be a better predictor of vascular complications than traditional Turner z score analysis.^293^ The AHA recently published a scientific statement on the management of Turner syndrome and recommends CMR at diagnosis and CMR surveillance due to the high incidence of undiagnosed vascular anomalies such as coarctation, PAPVC, systemic venous anomalies and coronary anomalies. In low risk Turner syndrome patients, imaging surveillance can be performed every 5–10 years, but with increased frequency of surveillance in patients with high ASIs (> 2.3 cm/m^2^).^294^

The natural history of most genetic aortopathies is one of a thinned aortic wall which progressively dilates and loses distensibility thereby heightening the risks of aneurysm formation and dissection throughout its length, but particularly at the root.^295^ Cohort studies have shown that nearly 60% of MFS patients will have aortic root dilatation by age 35 years and that the aorta increases further with age.^296–298^

#### Valvular Disease

As the root dilates, aortic leaflets fail to fully coapt and AR increases. Echocardiography is the mainstay of AR surveillance but a systematic review of 11 articles comparing quantification of AR by CMR to semiquantitative evaluation with echocardiography confirmed that direct aortic measurements using PC-CMR at the sinotubular junction/aortic valve are highly reproducible and accurate and predicts those who progress to surgery with high overall sensitivity and specificity.^299^ Similarly, the 2014 AHA/ACC guidelines recommend CMR for quantification of AR in patients with moderate or severe AR with suboptimal echocardiography images and evaluation of LV systolic function and volumes.^300^ Atrioventricular valve disease is more common in MFS compared to other genetic aortopathies with some studies demonstrating > 65% prevalence of mitral valve disorders. Infants and children with MFS present early with tricuspid and mitral valve fibroxanthomatous thickening and prolapse.^301,302^ CMR has emerged as an important noninvasive modality to diagnose and characterize MVP and help quantify mitral regurgitation.^303–306^

#### Left Ventricular Function

Dilated cardiomyopathy is an under-recognized clinical feature of aortopathy syndromes. Myocardial dysfunction is often considered to be the consequence of valvular insufficiency and ventricular volume overload,^307^ however, in a subgroup of aortopathy patients without significant valvular dysfunction or aortic dilatation, subclinical cardiac dysfunction has been described,^308^ attributed to a combination of increased aortic wall stiffness and extracellular matrix abnormalities. CMR is the reference standard for evaluating biventricular function as mentioned previously^3,309^ and can be used to evaluate ventricular performance.

#### Systemic Vascular Screen

**Figure 32.**
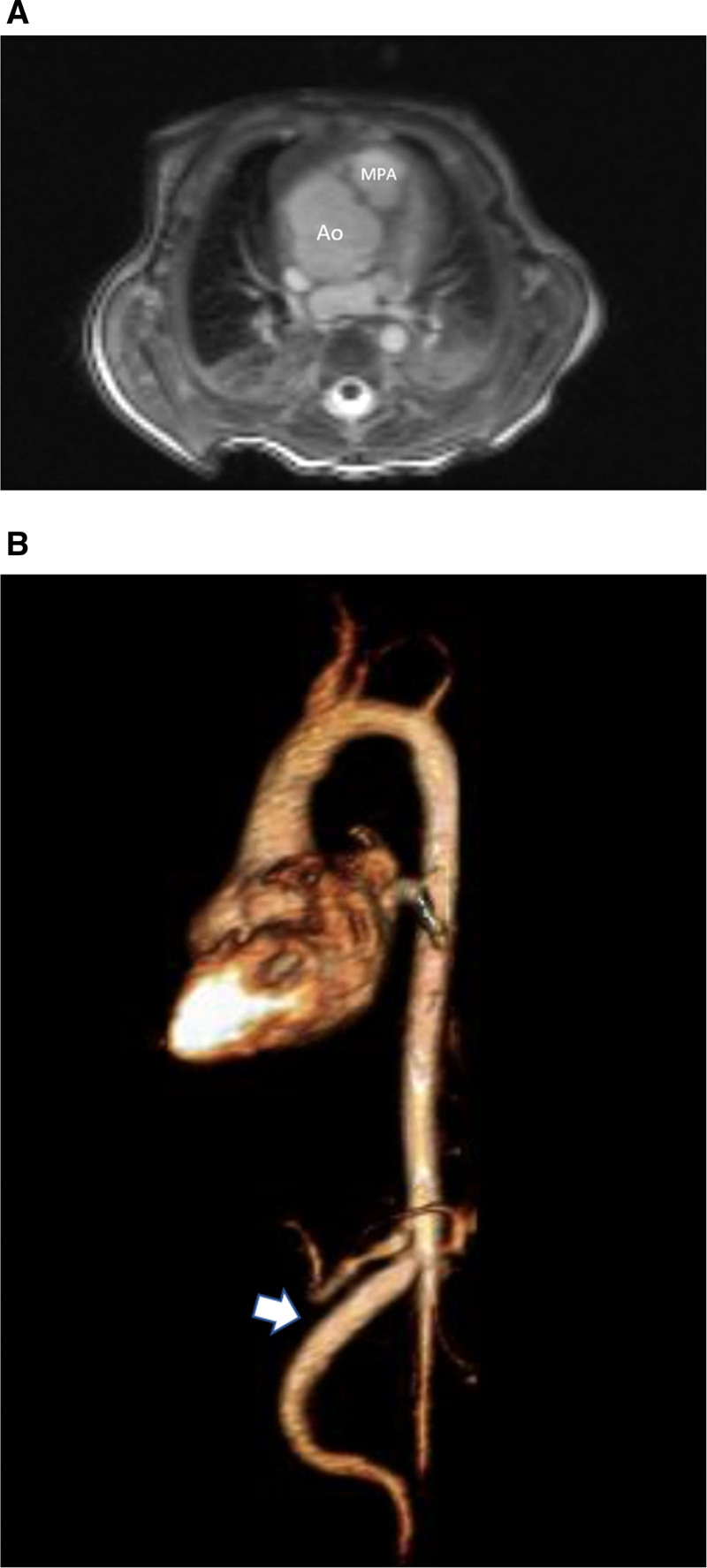
A 1 day old with Loeys-Dietz syndrome. **A**, Axial oblique bSSFP image in aortic root plane showing massively dilated aortic root with a diameter of 2.2 cm (z score of 8.7) (**B**) 3D gadolinium enhanced image showing diffuse aneurysmal dilatation of the superior mesenteric artery (arrow).

Although aortic aneurysms generally occur at the aortic root in many patients (Figure 32), approximately 50% of patients with LDS syndrome have aneurysms remote from the aortic root, including the head and neck vessels, intracranial berry aneurysms, abdominal branch arterial aneurysms and iliac artery aneurysms. Current recommendations are that LDS patients have annual CT/CMR examination from head to pelvis. CMR, which avoids ionizing radiation and ability to combine both non-contrast and contrast enhanced angiography to achieve whole body evaluations, has distinct advantages over a full body CT with runoff, especially for repeat examinations over time. Non-vascular abnormalities including Chiari I malformation, vertebral subluxations, diaphragmatic hernias, pectus abnormalities and scoliosis are frequently unsuspected clinical findings found on whole body evaluations.^310^ Patients with Turner syndrome have increased incidence of PAPVC, systemic venous anomalies such as left SVC/interrupted IVC, congenital portosystemic shunts and coronary artery anomalies, many of which are not appreciated on echocardiography, yet relatively easily appreciated on CMR.

#### Aortic Dissection

Aortic dissection is a catastrophic complication of aortic wall disease associated with high mortality and morbidity. In acute symptomatic dissection, rapid imaging diagnosis is essential. CMR can be used to diagnose dissection, distinguish aortic pseudoaneurysm from dissection and assess intimal flaps and aortic branch vessel involvement.^311–313^ Multiple CMR sequences, both contrast and non-contrast enhanced, all provide specific and unique information including exact positioning of the intimal flap and ability to distinguish slow flow from thrombus in the false lumen.

#### Vertebral Tortuosity Index (VTI)^314^

**Figure 33.**
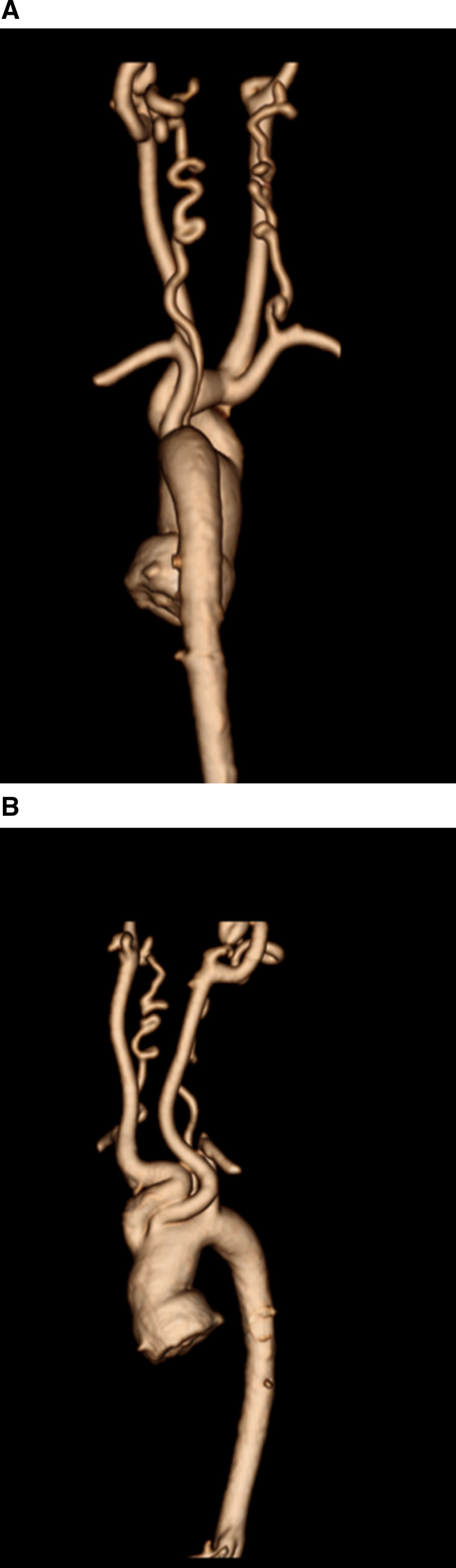
A 10 year old with Loeys-Dietz syndrome. **A** and **B** 3D gadolinium enhanced shaded surface displays showing a dilated ascending aorta, dilated bilateral carotid arteries and tortuous bilateral vertebral arteries. Vertebral tortuosity Index was 95 on right and 65 on left.

Increased arterial tortuosity of the head and neck vessels, particularly the vertebral arteries, has been described in LDS, MFS and Turner syndrome (Figure 33) and can be calculated easily by CMR. VTI is defined as [(actual vertebral artery length/straight vertebral artery length-1) × 100] measured from vertebral artery origin to C2 if included in the study. Higher VTIs (> 50) have been associated with major adverse clinical outcomes, including a more severely dilated aortic root, increased rate of cardiac surgery, younger age at dissection and death.^314^

#### 4D-Flow CMR in Aortopathies

**Figure 34.**
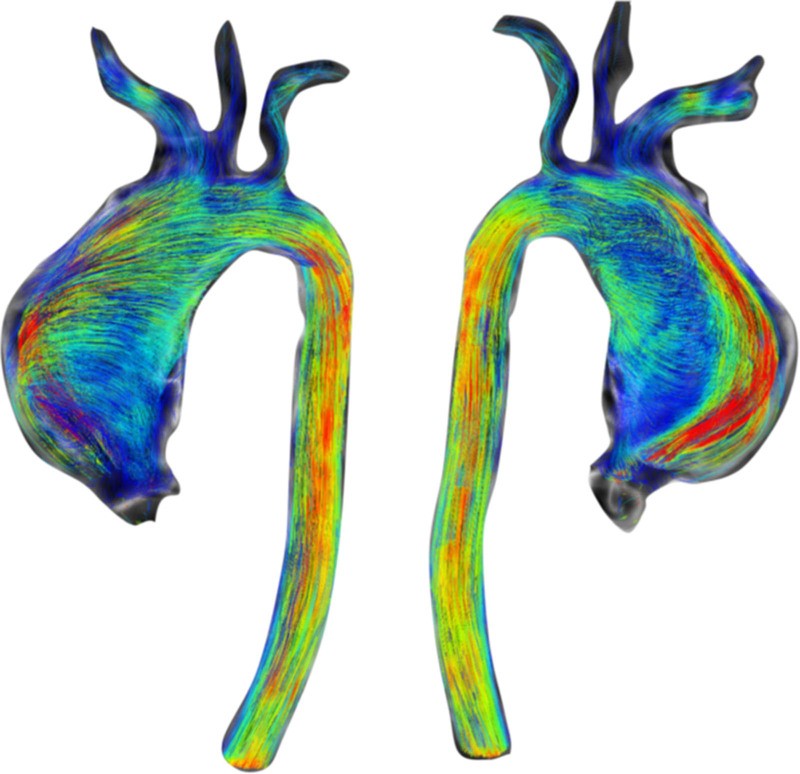
A 17 year old with severe aortic dilatation. Sagittal multiplanar reformatted images from 4D-flow CMR showing severe ascending aortic dilatation extending to involve the common origin of the innominate and left common carotid arteries.

(Figure 34) Hemodynamic quantification is a major advantage of CMR over other imaging modalities in genetic aortopathy. Multiple studies have demonstrated that BAV-related aortopathy patients, expressing abnormal wall shear stress, may develop significant alterations in elastin fiber and extracellular matrix protein compositions predisposing patients to further aortic dilatation and heightened risk for dissection.^315,316^ Less is known about genetic aortopathies, however, emerging 4D- flow CMR data suggests that similar findings are present in pediatric genetic aortopathy patients and become more pronounced over time.^317,318^

*Advantages of CMR* MR has been shown to be very accurate in the diagnosis of thoracic aortic disease with sensitivities and specificities that can exceed echocardiography.^319–321^ CMR provides a multiplanar evaluation of the thoracic aorta and its branch vessels and can conceivably cover the entire systemic arterial circulation from head to toe. CMR has the additional ability to evaluate the competence of the valves and assess LV function in addition to a detailed morphological assessment of the thoracic vessels and additional musculoskeletal abnormalities that are related to aortopathies. The modality is also very accurate in the diagnosis of aortic dissection but in cases of suspected acute dissection, CT may be preferred to speed of examination in a potentially unstable patient. CMR is also preferred for VTI calculation due to the beam hardening CT artifacts related to the intraforaminal courses of these vessels. There is a plethora of CMR literature in pediatric genetic aortopathies.^290,314,317,318,322–327^

#### Summary of Recommendations

- CMR is the recommended imaging modality for aortic size measurement and surveillance in pediatric patients with genetic aortopathy, as well as assessment of valve regurgitation and LV function. (Class I, Level of evidence B).
- CMR should be utilized for complete arterial screening and surveillance in pediatric patients with genetic aortopathy (Class I, Level of evidence C).
- Depending upon the risk of the mutation, serial CMRs should be performed every few years (Class I, Level of evidence C) and if stenosis, dilation or aneurysm are determined to be progressing, serial CMRs should be occur more frequently (as much as every 6 months) (Class I, Level of evidence C).
- CMR is indicated for assessment of aortic dissection risk: In patients with Turner syndrome with additional risk factors, including BAV, coarctation of the aorta, and/or hypertension, and in patients who attempt to become pregnant or who become pregnant, it may be reasonable to perform imaging of the heart and aorta to help determine the risk of aortic dissection. (Class I, Level of evidence C).
- CMR may be beneficial for VTI measurement for risk stratification in patients with genetic aortopathy risk: Limited populations examined have suggested that VTI > 50 is associated with adverse clinical outcomes. (Class I, Level of evidence B).

### Vascular Rings and Slings

#### Background

Vascular rings and slings are rare congenital anomalies involving the aortic arch and PAs, representing about 1–3% of CHD.^328,329^ The vessels encircle the trachea and esophagus and can lead to varying degrees of compression and clinical symptoms involving the respiratory and/or gastrointestinal system. Some patients are asymptomatic, diagnosed incidentally when imaging is needed for other unrelated reasons and do not require treatment.^329^ Visualization of the entire ring or sling can be challenging due to indirect imaging of structures with chest radiography or barium esophagram and suboptimal acoustic windows with echocardiography. Due to their significant respiratory symptoms, some patients may first undergo bronchoscopy, revealing a pulsatile stenosis and tracheomalacia, again indirect evidence of a vascular anomaly.^330–334^ In addition, the vascular structures completing the ring may be atretic, or unable to be opacified with any imaging modality.^333,335,336^

#### Indication for CMR in Vascular Rings and Slings

There is an extensive history of the utilization of CMR to diagnose aortic arch anomalies^330,335,337–347^ CMR provides many advantages for the assessment of vascular rings and slings including imaging of the airway and allowing a conclusive diagnosis to guide in therapeutic management.

##### Vascular Rings

**Figure 35.**
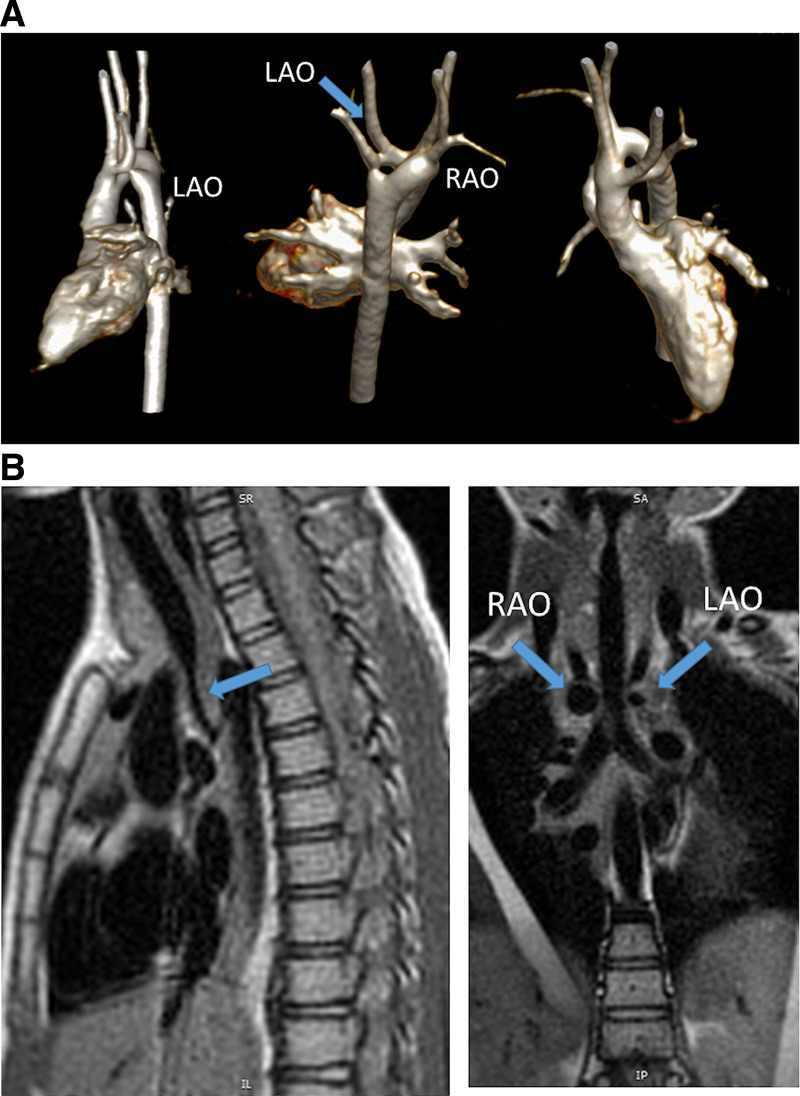
A right dominant double aortic arch. **A**, Three different views from a contrast enhanced 3D volume rendering from the lateral (left), posterior angled cephalad (middle) and anterior angled caudad (right). Note the ring in the center. **B**, Dark blood imaging of the trachea in the sagittal (left) and coronal views (right). Note the narrowing distally on the sagittal view and how both arches can be visualized in cross-section on the coronal (arrows). LAO indicates left aortic arch; and RAO, right aortic arch.

The most common type of vascular ring, accounting for about 50–60% of cases, is double aortic arch, with persistence of both fourth arches. The double aortic arch may be right-dominant, left-dominant, or codominant, though right dominance occurs in the large majority (Figure 35). Portions of the double arch may also be atretic.^333,334^ Neonates may present with life-threatening complications of the airway.^331^ With significant tracheomalacia, symptoms may persist after surgical intervention, with some cases requiring further tracheal surgery.^331^ CMR has been demonstrated to successfully diagnose patent double arches and those with atretic portions and is superior to cardiac catheterization angiography in its ability to demonstrate associated compression of the airway.^330,331,335^

**Figure 36.**
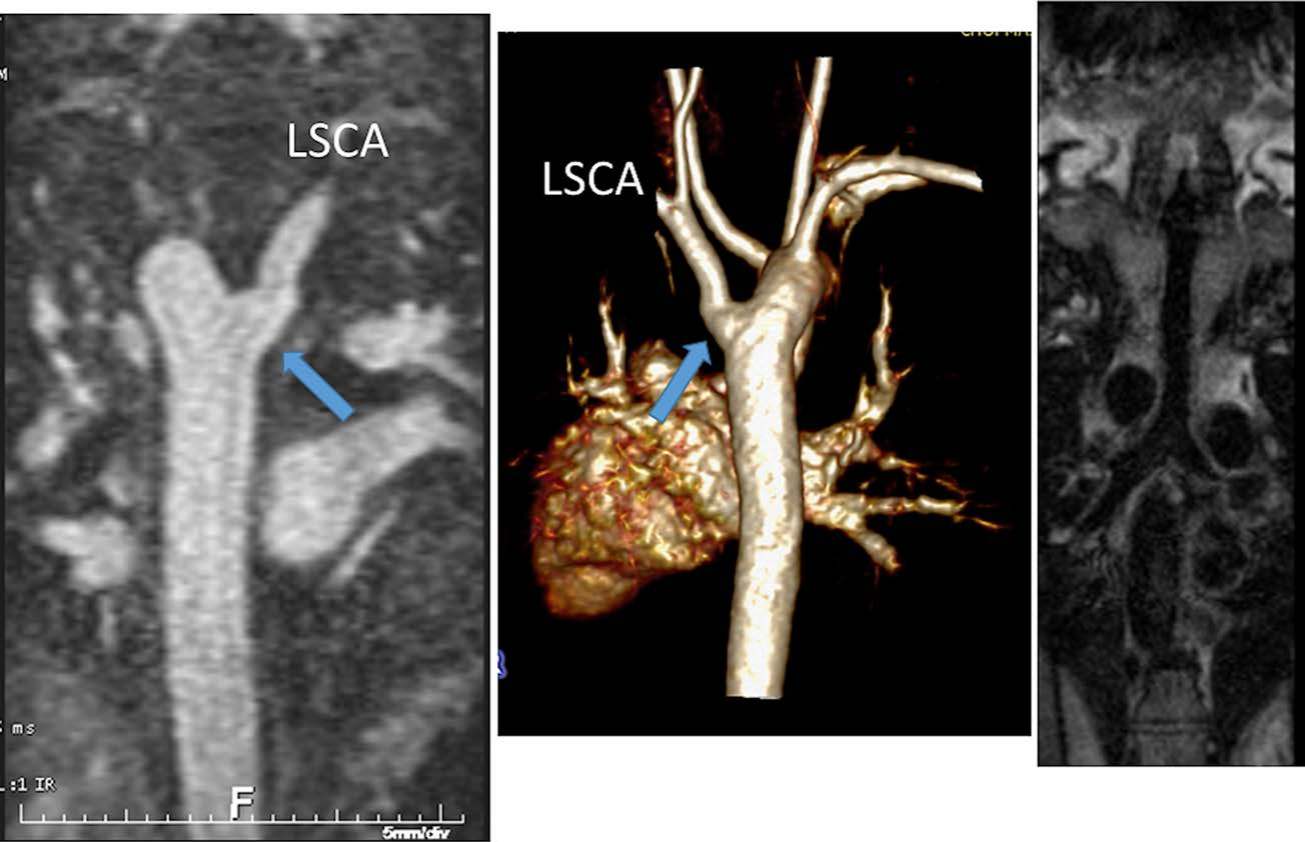
A right aortic arch with a diverticulum of Kommerell and an aberrant left subclavian artery (LSCA) in a 6 year old. The descending aorta is seen on the left with the diverticulum (arrow) and the LSCA originating as the last branch while the 3D shaded surface display is in the center of the image viewed posteriorly. The trachea from 3D dark blood imaging is visualized on the right.

The next most common vascular ring type involves a right aortic arch (30–35% of cases) with aberrant left subclavian artery, left ligamentum arteriosum and diverticulum of Kommerell (Figure 36). A small portion of patients with right aortic arch may have mirror image branching and a retroesophageal ductal ligament that can identified by an aortic “dimple” delineated on CMR imaging (diverticulum) in conjunction with tracheal and esophageal compression.^333,348^ Both double aortic arch and right aortic arches constituting vascular rings have been known to occur in conjunction with CHD such as TOF.^349^

**Figure 37.**
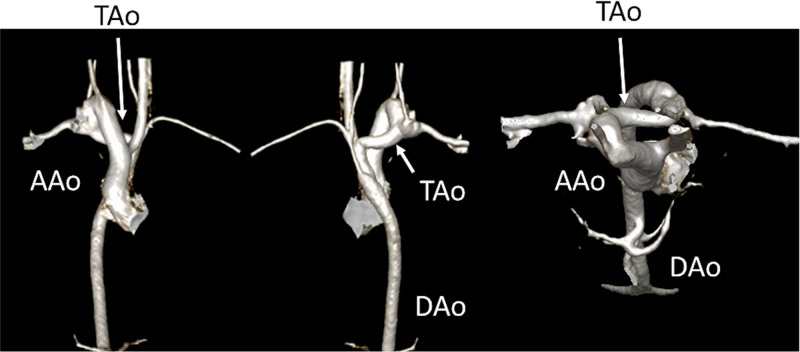
A cervical circumflex right aortic arch as demonstrated by a contrast enhanced volume rendered display as viewed from anterior (left), posterior (middle) and from superior (right). The ascending aorta (AAo) ascends on the right and the transverse aortic arch (TAo) crosses posteriorly to the left, posterior to the trachea and esophagus and the descending aorta (DAo) descends on the left.

Circumflex aortic arch is more rare type of vascular ring with contralateral locations of the transverse aortic arch over the bronchus and descending aorta where there is a “retroesophageal segment” of the aortic arch; that is to say a right aortic arch with a left descending aorta or a left aortic arch with a right descending aorta (Figure 37). Over half of patients have additional cardiac anomalies and some may also have a “cervical” aortic arch. CMR has much higher sensitivity for diagnosing the circumflex aortic arch when compared to chest radiography and echocardiography. Accurate diagnosis is essential for surgical planning.^342^

**Figure 38.**
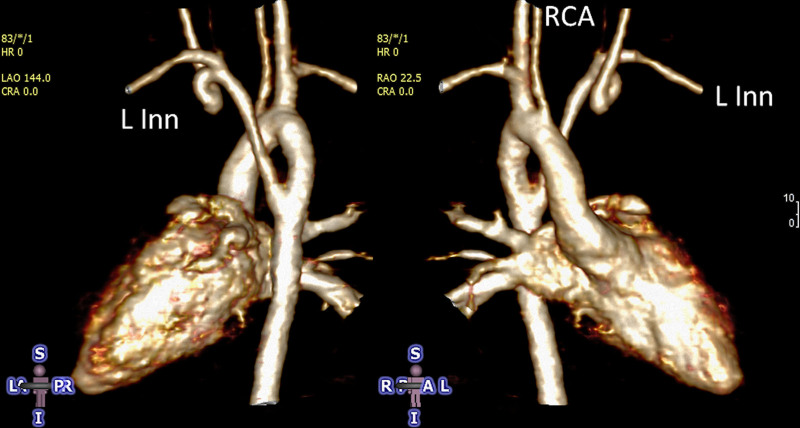
3D volume rending of a right aortic arch with an aberrant left innominate artery (L Inn) visualized from posterior (left) and anterior (right). RCA indicates right carotid artery.

Other rarer vascular rings with varying location of aberrant vessels and the ligamentum arteriosum (eg right aortic arch with an aberrant left innominate vein) have all been optimally imaged with CMR (Figure 38).

##### Pulmonary Artery Slings

**Figure 39.**
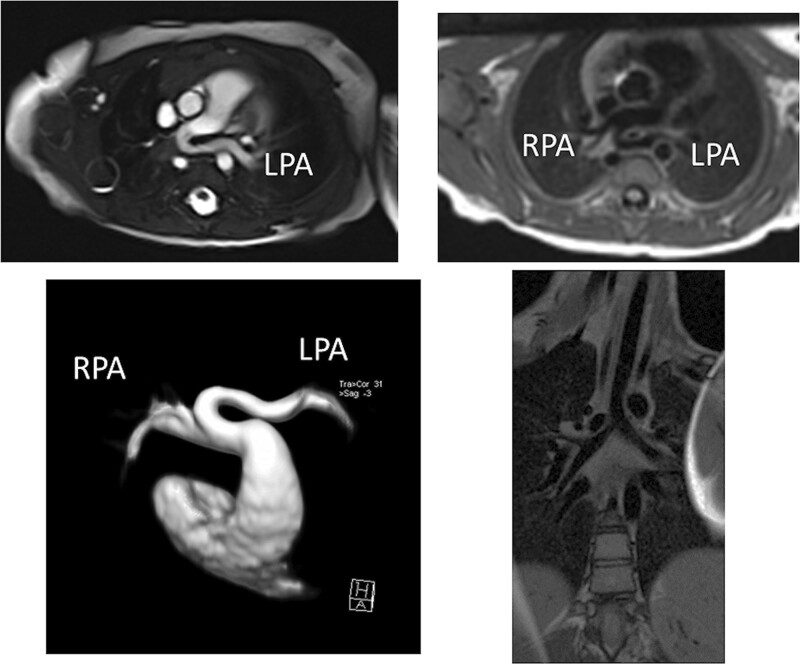
A pulmonary artery sling where the left pulmonary artery (LPA) arises from the right pulmonary artery (RPA) in a one year old. Off axis axial CMR cine (upper left), dark blood (upper right) and 3D reconstruction (lower left) demonstrate the anatomy. Lower right displays an off-axis coronal view of the trachea.

A PA sling is the anomalous origin of the left PA from the right PA which courses between the trachea and esophagus, creating a “sling “around the distal trachea or proximal right bronchus (Figure 39). Impingement of the airway leads to respiratory symptoms and compression of the esophagus commonly causes dysphagia and vomiting. The ability to diagnose PA sling is equivalent with CT and CMR (although to diagnose a complete tracheal ring, CT should be utilized), though CMR avoids ionizing radiation, iodinated contrast and flows to both lungs can be evaluated.^331,339,340^

#### Considerations

Vascular rings and slings may present with life-threatening complications of the airway in the newborn period. Upper airway obstruction and dysphagia are common presenting symptoms during this time and later in infancy or early childhood and beyond. Prolonged or recurrent respiratory symptoms or refractory asthma may necessitate workup for vascular anomalies. While chest radiography, barium esophagography, bronchoscopy and echocardiography are suggestive of a vascular ring or sling, full details of the vascular anomaly and impacted structures (esophagus, trachea, bronchi) must be delineated further for adequate surgical management, and these can be provided by CMR. As some surgeons prefer a 3D reconstruction of the great vessels prior to operating on arch anomalies, CMR can faithfully create a 3D model using both contrast enhanced and non-contrast techniques.^332^ CT can create 3D reconstructions of the great vessels as well as the trachea and can be utilized for this in centers that do not have the expertise in CMR. Invasive angiography is not indicated for the sole diagnosis of vascular rings and slings and have been replaced by CMR,^331,333,334^ but may be utilized in the setting of additional intracardiac defects needing intervention.

#### Summary of Recommendations

- After preliminary assessment and clinical suspicion for a vascular ring or pulmonary sling, CMR is indicated for definitive anatomic diagnosis in the management of these lesions prior to therapeutic intervention. (Class I, level of evidence B) Flows to both lungs may be assessed in pulmonary sling.
- CMR is indicated for the assessment of tracheal narrowing in patients with vascular rings and slings (Class I, level of evidence B)
- CMR is indicated for the assessment of associated lesions of vascular rings such as TOF (Class I, level of evidence B)

### TGA With a Systemic RV (Corrected TGA or TGA After Atrial Inversion)

#### Background

TGA, apart from those with a SV, may occur with a systemic RV in 2 different categories:

- Congenitally corrected transposition (ccTGA), also called L-looped TGA or TGA {S,L,L}, occurs when the morphologic LV is on the right side of the circulation and associated with the pulmonary valve and the morphologic RV is on the left side of the circulation and associated with the aorta.^350^ There is atrioventricular and ventriculoarterial discordance which results in normal hemodynamics where systemic venous returns is directed towards the lungs and pulmonary venous return is directed towards the body. These patients most commonly have no other CHD, but if CHD occurs, they most commonly have VSD, pulmonary stenosis or may have Ebstein’s anomaly of the left sided tricuspid valve.
- TGA after atrial inversion, also called TGA after atrial switch, occurs when patients with D-looped TGA (i.e. {S,D,D}; the morphologic RV is on the right side of the circulation and associated with the aorta and the morphologic LV is on the left side of the circulation and associated with the pulmonary valve) undergo an atrial switch operation, either a Senning or a Mustard procedure. In D-looped TGA, there is atrioventricular concordance and ventriculoarterial discordance resulting in the systemic and pulmonary venous systems in parallel circuits; the Senning and Mustard procedures baffle venous return to the correct ventricle physiologically. Most of these operations were performed in the era prior to ASO so the vast majority of these patients are adults although in some circumstances, this is performed in the "double switch" operation for TGA {S,L,L}.

Whether the RV can tolerate a lifetime of systemic vascular resistance and pressure, whether a morphologic D-loop or L-loop, has always been debated, making study of RV performance in this patient population crucial. In a multi-institutional retrospective analysis, by 45 years of age, 67% of those with ccTGA with intracardiac lesions and 25% without intracardiac lesions had heart failure and systemic ventricular dysfunction.^350^ Mechanics of the systemic RV are markedly different from the single RV and from the normal systemic LV.^30^ Both the RVs in ccTGA and TGA after atrial inversion can be dilated, spherical and poorly functioning.^351^ For those with TGA after atrial inversion, after a high short term mortality rate (up to 20%), there is a longer lower mortality rate which nevertheless remains significant.^352^ Multiple studies have demonstrated a significant decline in RV function in adulthood, with or without symptoms.^353,354^ Both RV and LV in the systemic RV circulation demonstrate abnormal response to exercise.^355^

#### Indications for CMR

**Figure 40.**
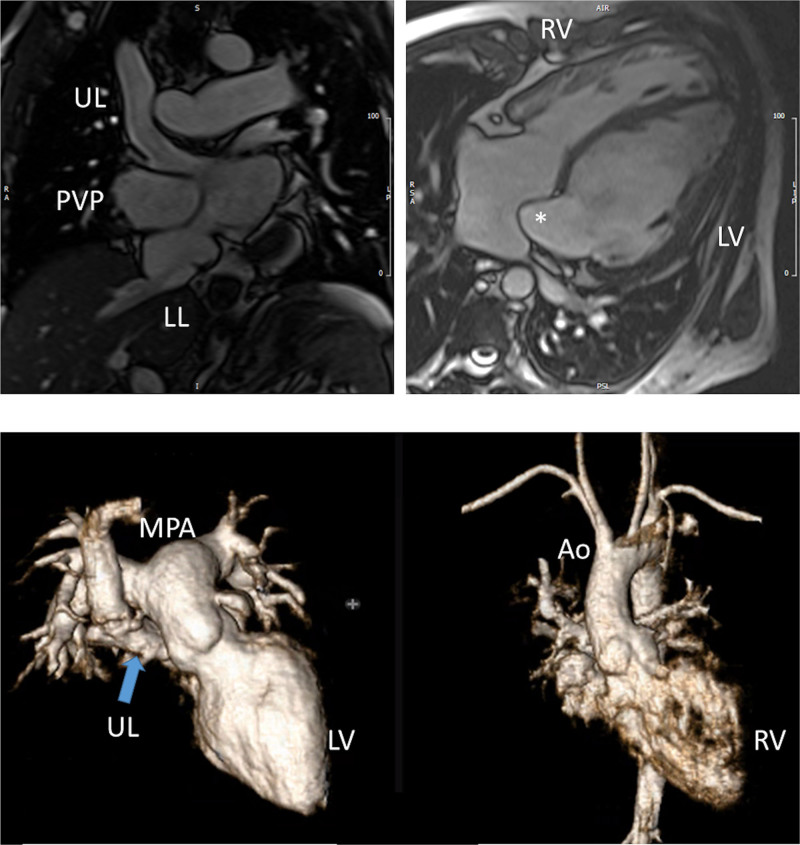
Transposition of the great arteries after atrial switch. **A**, Panel on the left is a cine of the upper limb (UL) and lower limb (LL) of the systemic venous pathway, in this case, a Mustard operation. On the right is a 4-chamber view with the asterisk denoting the distal end of the systemic venous pathway. **B**, 3D gadolinium volume rendering of the main pulmonary artery (MPA) arising from the left ventricle (LV) on the left and the aorta (Ao) arising from the right ventricle (RV). Note on the left that the UL of the systemic venous pathway can be seen (arrow).

**Figure 41.**
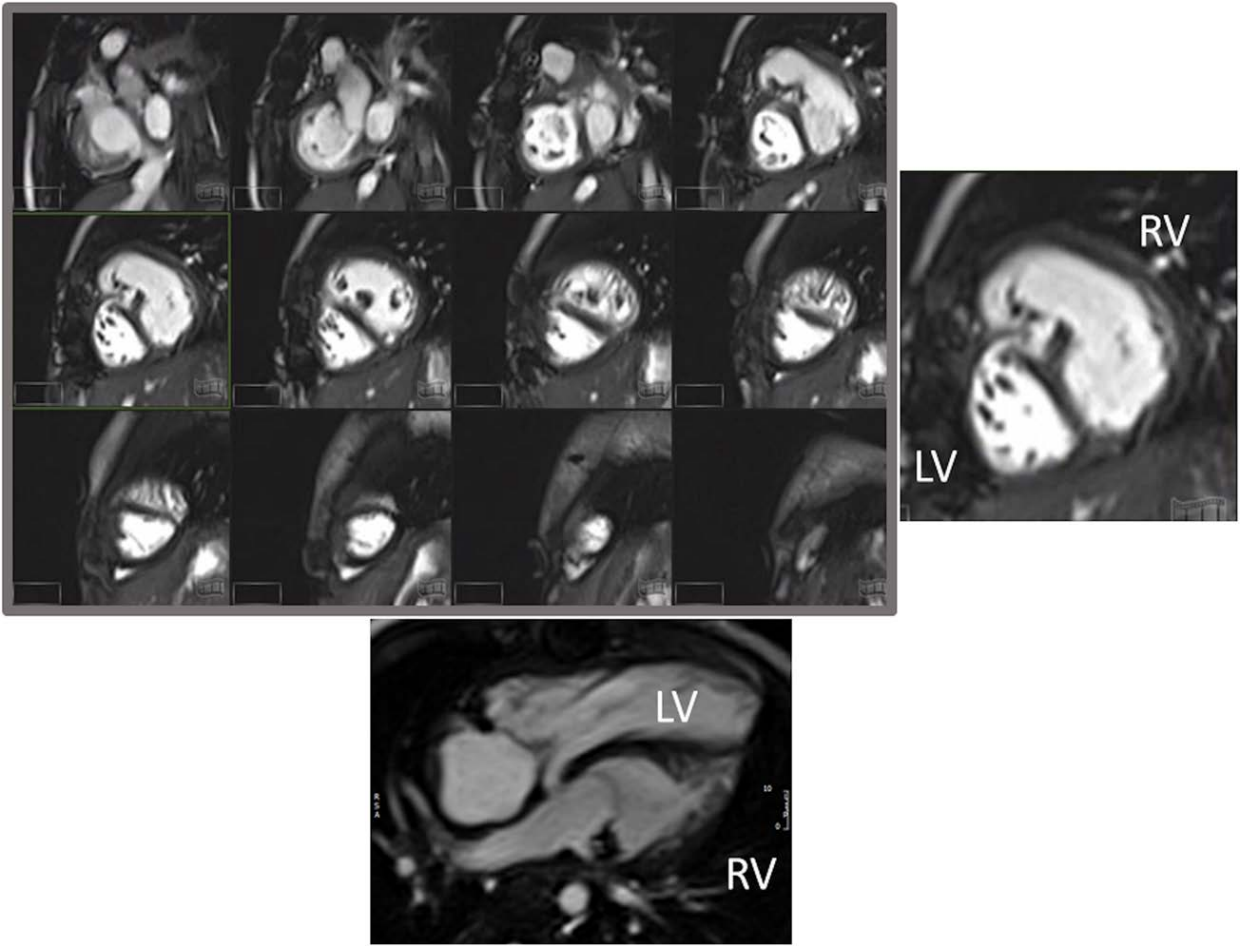
Corrected transposition of the great arteries (ccTGA). The upper left panel is a stack of bright blood cine images depicting the geometry of a 2 year old patient with ccTGA. The upper right panel is one slice showing the right ventricle (RV) on the left and the left ventricle (LV) on the right. The lower panel is a 4-chamber view.

CMR is ideally suited for the study of the systemic RV in the setting of TGA due to its ability to overcome the issues of RV geometry and retrosternal location that challenge echocardiography. The issue of RV geometry is very germane in these diseases as the RV remodels and the anatomy changes when it becomes a systemic ventricle. In addition, the geometry of a D-looped RV (Figure 40) as in TGA after atrial inversion is very different than that of an L-looped RV as in ccTGA (Figure 41). The use of CMR for quantification of RV function and ventricular volumes in these patients have been extensively published^351,355–362^ including the aforementioned abnormal response to exercise. Adverse cardiac events have been associated with RV volume, RV mass index, RVEF, RV wall stress and LV volume.^363–365^ There is evidence to demonstrate that as they age, patients with a systemic RV have functional decline and no resting parameters correlate with exercise function.^366,367^ Because of the ability to quantify ventricular performance independent of geometry, CMR has been utilized in several pharmacologic trials in this patient population.^368,369^ Systolic strain by CMR has also been quantified using feature tracking methods^370^ and dyssynchrony, as measured by feature tracking, correlates well with major cardiac events in those with a systemic RV.^363^

In patients with TGA after atrial inversion, CMR has been utilized for several decades to assess the size, patency and leaks of intra-atrial baffles^371–373^ (Figure 40) as well as ventricular outflow tract obstruction.^357,359,374^ It is not uncommon to visualize obstruction to the superior limb of the systemic venous pathway^375^ or right to left shunts due to baffle leaks by cine.^359^ PC-CMR is utilized to determine the size of the shunt (Qp/Qs) with internal checks along with in-plane velocity mapping to visualize the leak. Ventricular outflow tract obstruction is assessed by CMR on a routine basis.^376^

Similar to the debate regarding whether the RV can tolerate a lifetime of systemic vascular resistance and pressure, the ability of the morphologic tricuspid valve to maintain systemic atrioventricular valve work is also questioned. TR is generally due to annular dilation and systemic RV dysfunction in ccTGA,^377^ however, this may also be due to Ebstein’s anomaly of the left sided tricuspid valve^378^; either way, it presents a volume load on the ventricle. Indeed, in the presence of significant TR and if present, deteriorating RV function, a “double switch” may be performed, combining an ASO procedure with a Senning^379^ and CMR is utilized not only to evaluate the intra-atrial baffle but also the coronary arteries and ventricular function. CMR, as previously noted, can measure atrioventricular valve insufficiency via 2 methods utilizing PC-CMR alone or in combination with cine CMR. There is evidence to demonstrate that the systemic RVEF cannot increase in response to increasing atrioventricular valve insufficiency.^380^ Ultimately, though, accurate measurements of TR by CMR are important as this has been linked to ventricular function and clinical outcomes in this patient population.^381–383^

Other anatomic abnormalities can be assessed by CMR including VSD repair, pulmonary stenosis and PR after repair and the size of the branch PA in patients with ccTGA (Figure 41). PC-CMR is used to determine Qp/Qs similar to baffle leaks along with quantification of flows to both lungs and PR fraction similar to TOF.

**Figure 42.**
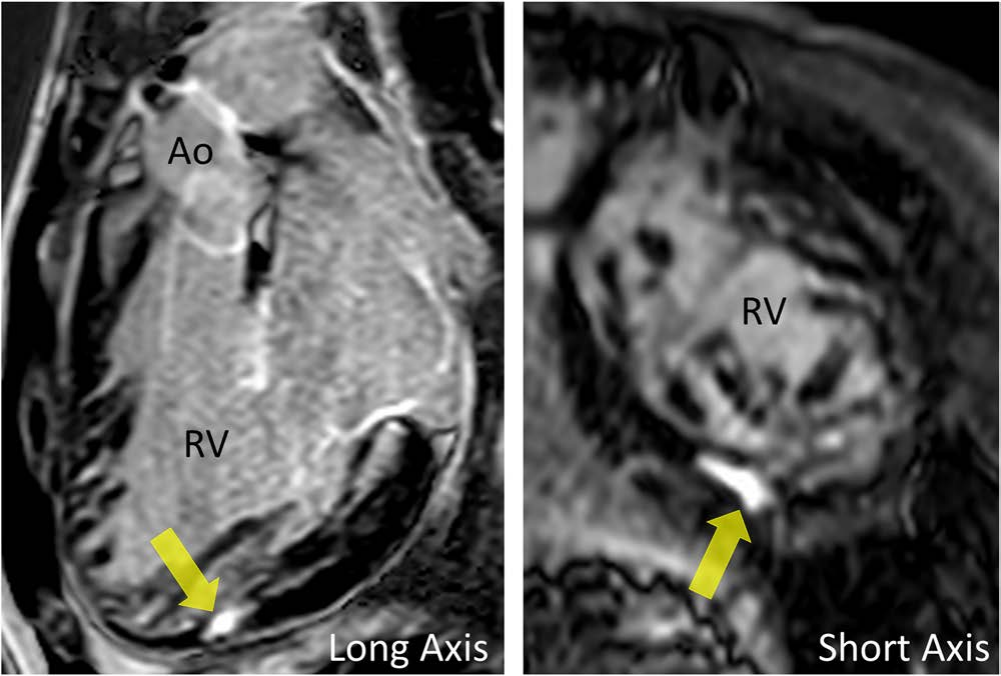
Myocardial scarring in the right ventricle (RV) in a patient with ccTGA from 2 views. Ao indicates aorta.

Characterization of the myocardium with CMR has demonstrated a role for the assessment of myocardial fibrosis and its link to the gradual decline of the systemic RV (Figure 42). The presence of LGE is associated with older age, RV volume and function, QRS duration, and prior arrhythmia or syncope.^384^ In a study of 34 patients with systemic RVs, LGE was linked not only to older age, lower RVEF and arrhythmia but also higher RV wall stress, reduced peak oxygen uptake during exercise and a worsening of clinical symptoms.^365^ LGE in the systemic RV is related to collagen content^385^ as well as ventricular dyssyncrhony.^386^ Diffuse fibrosis, using ECV, has also been assessed in the systemic RV in a dual chambered circulation.^387,388^ Importantly, the presence of myocardial fibrosis is prospectively related to a mix of adverse clinical outcomes in follow up.^389,390^ These types of studies have been important in understanding the role of fibrosis in the natural history of the systemic RV, and may indirectly imply a role of anti-fibrotic medical therapy.

#### Summary of Recommendations

- CMR is indicated for quantification of systemic RV volumes, mass, and ejection fraction (Class I, level of evidence B) and can be useful for quantification of pulmonary LV performance parameters in patients with a dual chambered circulation (Class IIA, level of evidence C)
- CMR is recommended for the assessment of the systemic atrioventricular valve (left sided tricuspid valve in TGA {S,L,L} or right sided tricuspid valve in TGA {S,D,D} after atrial inversion procedure) in patients with a systemic RV (Class I, level of evidence B)
- CMR is useful for detecting systemic RV myocardial fibrosis, which may have important implications for a given patient’s prognosis and therapy options (Class I, level of evidence B)
- CMR is beneficial for the detection of stenosis and leaks of the interatrial baffle in those patients with systemic RVs who have undergone an atrial inversion procedure (Class I, level of evidence B) as well as for detection of the presence and severity of outflow tract obstruction (Class I, level of evidence B).
- CMR is useful to assess associated lesions and the sequelae of repair such as VSD, pulmonary stenosis and PR, especially in patients with ccTGA (Class I, level of evidence B).

### Hypertrophic Cardiomyopathy

#### Background

Hypertrophic cardiomyopathy (HCM) is a disorder of increased LV mass and may be differentiated into syndromic (with other systemic involvement) and nonsyndromic (without other systemic involvement) types. This section describes nonsyndromic disease. This type of HCM is a common genetic cardiomyopathy resulting from autosomal dominant mutations in multiple genes encoding the cardiac sarcomere; including cardiac β-myosin heavy chain, cardiac myosin binding protein C and Troponin T. There have been over 1400 mutations described.^391^ On histopathology, HCM is characterized by myocyte disarray, myocardial hypertrophy and fibrosis along with abnormal small intramural arterioles with thickened walls and narrowed lumen resulting in ventricular dysfunction and ventricular arrhythmias. The presentation of HCM is heterogenous with varied symptomatology including fatigue, chest pain, palpitations and even sudden cardiac death (SCD). Treatment options include medical management, surgical myomectomy or alcohol septal ablation for relief of critical LV outflow obstruction (gradient ≥ 50 mmHg), and implantable cardioverter and defibrillators (ICD) for those patients considered to be at highest risk for SCD.

Guidelines for the diagnosis and treatment of HCM have been published by both the European society of Cardiology (ESC) and the ACC/AHA.^392,393^ Both sets of guidelines have similar requirements regarding the diagnosis of HCM, which in adults is defined as a left ventricular wall thickness (LVWT) of > 15 mm in one or more myocardial segments, not explained by loading conditions. In children, the diagnosis requires an LVWT > 2 standard deviations from the predicted mean (z score > 2).

Risk stratification for SCD in pediatric HCM has also been extrapolated from these adult based guidelines as suggested in the current ACC/AHA guidelines.^393^ The ESC guidelines differ from the ACC/AHA in that the ESC uses a risk prediction model to guide use of implanted cardiodefibrillator (ICD). In addition, the ESC guidelines are only intended for adult use while the ACC/AHA guidelines are intended for both adult and pediatric use. In the ACC/AHA guidelines, focus on the presence of at least one clinical risk factor for SCD (LVWT ≥ 30 mm, syncope, nonsustained ventricular tachycardia (NSVT), family SCD history or abnormal blood pressure response to exercise) as class IIa indications for the implantation of ICD as primary SCD prevention. Recently, there have been attempts at creating risk calculators solely for pediatric use.^394–396^

#### Indications for CMR

##### Quantification and Distribution of LV Hypertrophy

**Figure 43.**
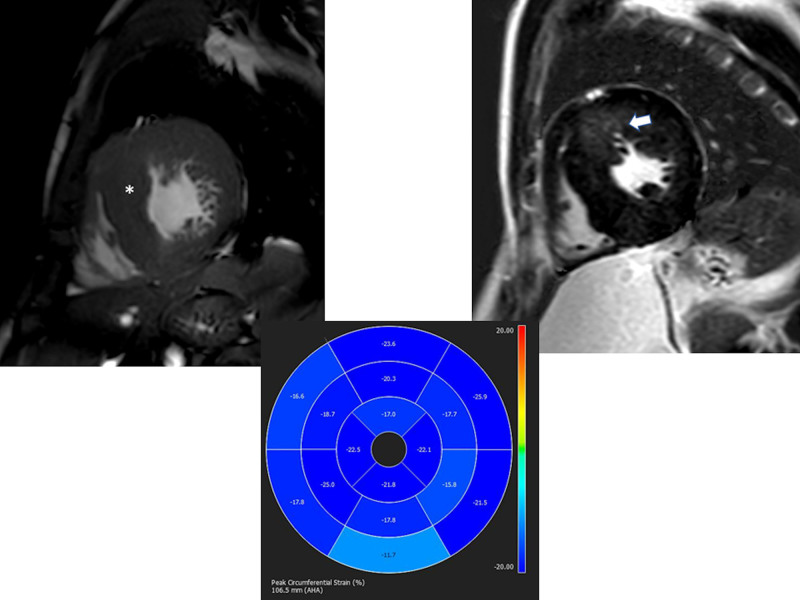
A 14 year old with hypertrophic cardiomyopathy (HCM). Upper left panel is a cine bSSFP sequence in short axis plane demonstrating diffuse LV myocardial hypertrophy affecting the basal septum the most (*) with phase sensitive inversion recovery image demonstrating corresponding late gadolinium enhancement (upper right, arrow). Myocardial Strain “bullseye” map demonstrating globally decreased LV myocardial circumferential strain (lower panel).

The diagnosis of HCM is characterized by an increased diastolic LVWT in at least one segment in the presence of a nondilated LV chamber (Figure 43) which is readily identified on CMR at any age. Increased LVWT may be present at birth or may develop during childhood and adolescence despite previously normal echocardiographic evaluations, necessitating serial evaluations in patients with gene positive or suspected HCM.^397^ One of the critical indications for pediatric CMR in this disease is the identification of increased LVWT not visualized by echocardiography but visualized by CMR.^398,399^ This is not a trivial occurrence particularly when the increased LVWT is focal and limited (as is not infrequently the case in pediatrics) which is present in up to 5% of children. This leads to an HCM diagnosis that would have otherwise been missed with CMR solely responsible for diagnosis.^400–402^

The most common pattern of HCM is increased LVWT in the basal anteroseptal region which is frequently associated with LVOT obstruction and mitral valve leaflet elongation. Other phenotypes include diffuse hypertrophy involving more than 50% of the myocardium, reverse septal contour, apical aneurysm^403^ and apical HCM which may manifest clinically with arrhythmias, diastolic dysfunction and small cavity size,^404,405^ all of which can be delineated comprehensively by CMR. RV hypertrophy is additionally observed in a third of adult HCM patients, most commonly at the RV septal hinge points.^406^ CMR derived LVWT measurements have a primary role in risk stratification with massive LV hypertrophy (LVWT ≥ 30 mm) associated with the highest risk of SCD.^407,408^ A meta-analysis of 25 pediatric HCM studies found a similar significant association between extreme LVWT and SCD in children and young adolescents. The hazard ratio for LVWT was 1.80 (95% CI 0.75–4.32, p = 0.19, I2 = 21%). The odds ratio estimate for extreme LV hypertrophy was 1.70 (95% CI 0.85–3.40, p = 0.13, I2 = 31%). For pediatric patients, new guidelines suggest that a maximal LVWT with a Z score ≥ 20 or > 10 in conjunction with other risk factors is reasonable as a risk factor for SCD.^393^

##### Biventricular Functional Assessment

As mentioned, CMR is the reference standard for evaluating biventricular function.^3,309^ bSSFP sequences performed in the standard planes provide accurate measurements of LVEF, chamber size and LV mass^409^ (Figure 43) which can be used serially for surveillance in suspected or confirmed HCM. Over time, a gradual transition from a hypertrophied, non-dilated LV with hyperdynamic systolic function to one of reduced systolic function can be seen in adults although this is uncommon in children. This is termed end-stage HCM and is generally regarded as having unfavorable outcomes with a mortality rate of 11% per year.^406,410^ In a large cohort of adult HCM patients, those with the lowest LVEF had the largest ventricular sizes and degrees of LGE by CMR, suggesting advanced remodeling in end-stage disease. Conversely, those with hyperdynamic systolic function had the lowest amount of LGE. Some pediatric HCM patients develop abnormal diastology with restriction which may manifest on CMR as left atrial dilatation, without LV dilation and is associated with poorer outcomes.^411^

##### Assessment of Dynamic Left Ventricular Outflow Tract (LVOT) Obstruction

Dynamic obstruction of the LVOT due to mitral valve systolic anterior motion (SAM) as well as due to septal hypertrophy is one of the leading causes of exercise intolerance in HCM patients and may necessitate invasive treatment measures such as myomectomy or septal ablation. LVOT gradients are conventionally measured using echocardiography, but CMR can identify the site of obstruction and assess whether there are contributing anomalies such as anomalous insertion of the anterior papillary muscle and elongated mitral valve leaflets (mitral leaflet length > 2 × the transverse dimension of the LVOT) at end systole.^412^ These findings are important in pre-operative planning in HCM patients who are considered surgical candidates by characterizing role of the mitral valve and sub-mitral apparatus and providing the surgeon an accurate estimate to the depth of the extended surgical resection of septal muscle necessary to achieve optimal relief of outflow obstruction.

##### Myocardial Fibrosis Assessment

**Figure 44.**
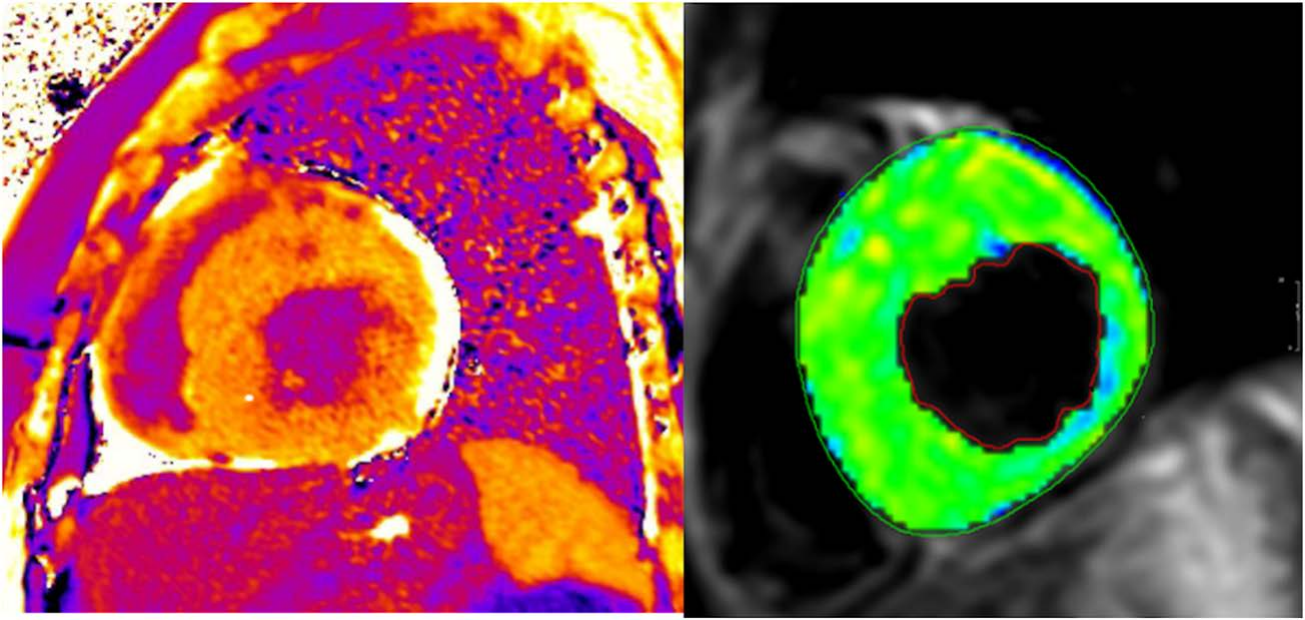
On the left is an extracellular volume map showing globally elevated elevated extracellular volume fraction (ECV) in a 14 year old patient with hypertrophic cardiomyopathy (HCM). On the right is a T2 map of left ventricular myocardium with T2 value of 69 ms ± 1.2 also in a 14 year old patient with HCM.

- LGE (Figure 43): The precise pathophysiologic mechanism responsible for LGE in HCM remains uncertain but is likely a combination of gadolinium deposition in areas of myocardial fibrosis and between areas of myocardial disarray. It is most prevalent in areas of hypertrophy and has been associated with increased incidence of ventricular tachyarrythmias and heart failure.^413,414^ LGE usually is patchy and midmyocardial in distribution. LGE has been described in 46–73% of children and adolescents with phenotypic HCM, despite preserved systolic function; it has been shown to increase annually with serial CMR surveillance, constituting on average 10.4% of LV mass.^415,416^ Those children with LGE were found to have greater LV mass and were at risk for adverse events including ventricular tachycardia,^417^ aborted SCD^418^ as well as having decreased ventricular strain (Figure 43).^416,419–422^ A 4-center study of 1,293 adult patients followed for 3.3 years showed that LGE of ≥ 15% was associated with a twofold increase in SCD event risk.^423^ Two subsequent meta-analyses that included that study and others confirmed that the incidence of SCD was increased in the presence of LGE, but differed in whether extent of LGE was important.^424,425^
- Diffuse fibrosis (Figure 44): Adult patients with HCM have abnormal T1 indices concordant with diffuse myocardial disease, even in the absence of LGE.^426–428^ Additionally, diffuse ventricular fibrosis by T1 mapping has been shown to be a predictor of non-sustained ventricular tachycardia and aborted SCD in adult HCM patients.^429^ In pediatric HCM patients, studies have demonstrated increased native T1 and ECV in hypertrophied areas of myocardium compared with non-hypertrophied areas and higher in LGE positive segments.^430,431^ T1 mapping can also be used to distinguish HCM from other potential causes of hypertrophy including hypertensive cardiomyopathy and the athletes’ heart.^432,433^ Nevertheless, more studies are needed in this area.

##### Exclusion of Other Diagnoses

Although HCM accounts for the majority of unexplained LV hypertrophy seen in adolescents, a number of other non-sarcomeric diseases can produce/mimic increased myocardial thickness including non-HCM causes of LVOT obstruction, hypertensive cardiomyopathy, LV noncompaction and metabolic/ infiltrative conditions including Anderson-Fabry’s disease and Friedrich’s ataxia. CMR can have a role in differentiating these conditions by identifying focal, limited hypertrophy not well visualized by echocardiography, distinguishing trabeculations from hypertrophy and identifying characteristic patterns of LGE. CMR may clarify and even alter the diagnosis in patients initially diagnosed with apical HCM who actually demonstrate LV non compaction on CMR.^434^

##### Myocardial Strain Assessment

The myocardial disarray, myocardial hypertrophy and abnormal papillary muscle orientation are all thought to contribute to abnormalities in myocardial strain by speckle tracking echocardiography in areas of hypertrophy and fibrosis. In addition, it has been associated with adverse cardiac events in pediatric and adult HCM.^435,436^ CMR feature tracking using standard cine bSSFP (Figure 43) can detect reduced LV myocardial mechanics (global and segmental longitudinal strain) in children and young adults with HCM and normal values in children have been published.^97^ This decrease in strain correlates with the degree of LVOT obstruction, LVWT and LGE^420–422^ as well as adverse events.^421^ Nevertheless, more studies are needed in this area.

##### Myocardial Perfusion

Adverse microvascular remodeling and coronary microvascular dysfunction (CMD) has been noted in HCM. Studies investigating myocardial perfusion in HCM patients have demonstrated abnormal perfusion metrics in both hypertrophied and non-hypertrophied segments of the LV, as well as correlation between the extent of perfusion abnormalities, LGE and degree of hypertrophy.^437,438^ A small pediatric study, demonstrated inducible myocardial ischemia in 7/13 pediatric HCM patients who were LGE negative suggesting that CMD may precede macroscopic fibrosis, however, it remains unknown whether CMD is an independent risk factor for SCD in HCM.^439^ Although promising, at present, it remains a research technique and more studies are needed.

##### T2 Weighted Imaging

Recent interest in T2 weighted imaging and its association with SCD has developed (Figure 44). Two studies investigating T2 signal in HCM patients are noteworthy. One demonstrated increased myocardial T2 signal intensity in patients classified as high risk for SCD and demonstrated positive correlation with the presence of > 15% LGE while the other demonstrated a positive correlation with life threatening arrhythmias.^440,441^ As with perfusion, although promising, at present, it remains a research technique and more studies are needed.

##### Coronary Artery Imaging

Myocardial bridging of the LAD is more common in HCM than in other causes of LV hypertrophy and may potentially be a mechanism for SCD, although there is no evidence to support this hypothesis. Myocardial bridging may be demonstrated by specific coronary CMR sequences (see Coronary section).

#### Advantages of CMR Over Other Modalities

Although TTE assessment of patients with HCM has been traditionally performed, limited acoustic windows, an inability to reliably capture focal hypertrophy and imaging plane obliquity may result in both under and overestimation of LVWT. CMR provides high contrast between bright blood and dark myocardium, excellent spatial resolution, and full biventricular coverage. In addition, short-axis images, derived perpendicular to the true LV long axis, allows LVWT measurements by CMR to be more precise and reproducible,^442^ enabling a diagnosis of HCM which may be missed by TTE.^400–402^ This holds true even in children, who have better acoustic windows than adults; a small study of pediatric HCM patients demonstrated that echocardiography derived measurements had poorer inter-observer and intra-observer reproducibility compared with CMR measurements.^443^ This may be due to superior visualization of the LV epicardial/endocardial borders with CMR, and visualization of all hypertrophied segments without any risk of obliquity^272,400,444^^.^

CMR is the only non-invasive modality that can assess tissue characteristics including the presence of macroscopic and global fibrosis, the former of which can be clinically utilized to risk stratify patients regarding potential ICD implantation. The emerging roles of parametric T1 mapping, myocardial perfusion imaging, and T2 imaging will likely increase our understanding of tissue characterization and may further stratify SCD risks.

#### Summary of Recommendations

- CMR is the recommended for confirmation of HCM diagnosis, evaluation of possible apical HCM or aneurysm and surveillance of LVWT in HCM^400,409,445^ (Class I, Level of evidence A).This includes patients with LV hypertrophy in whom there is a suspicion of alternative diagnoses including the athlete’s heart. In children and adolescents with a diagnosis of HCM, contrast enhanced CMR surveillance should occur every 3–5 years for risk stratification, evaluation of LGE, wall thickness and ventricular performance (Class I, Level of evidence B).
- CMR is the recommended to screen for HCM in patients with a family history of HCM when echocardiography is inconclusive (Class I, Level of evidence A)
- CMR is beneficial to monitor ventricular function when a more accurate measure than echocardiography is needed, when there is a concern for ventricular performance by echocardiography, or in selected patients for risk stratification for ICD placement^393^ (Class I, Level of evidence A). CMR is also useful to monitor LVOT obstruction in pediatric HCM patients when echocardiography is inconclusive (Class I, Level of evidence B)
- CMR is reasonable for the evaluation of myocardial fibrosis for risk stratification in pediatric HCM, possible ICD placement and to monitor the patient more closely than those without LGE. (Class I, Level of evidence B)
- CMR can be beneficial in pediatric patients with LV hypertrophy in whom alternative diagnoses in addition to HCM are suspected. (Class I, Level of evidence B)
- CMR strain measurements may be considered in pediatric HCM patients to monitor ventricular function as well as for risk stratification; patients should be monitored more closely if strain is below the lower limits of normal^97^ (Class IIb, Level of evidence B)

### Duchenne Muscular Dystrophy

#### Background

Duchenne muscular dystrophy (DMD) is an X-linked genetic neuromuscular disorder resulting in dystrophin protein mutation. DMD is one of the dystrophinopathies that also include Becker muscular dystrophy (BMD), and X-linked dilated cardiomyopathy. DMD results in the more severe phenotype presenting as skeletal muscle weakness early in life and often progressing to loss of ambulation early in the second decade of life. BMD is milder with a considerably more variable phenotype. The incidence of DMD is estimated to be ~ 1 / 5000 live male births.^446^ The United States prevalence is estimated to be 1.4 per 10,000 males.^447^ DMD patients also develop respiratory insufficiency. The restrictive lung disease is a result of diaphragm and secondary respiratory muscle weakness. Historically, the most common cause of death was respiratory failure. However, improved respiratory therapies have resulted in cardiomyopathy and resultant heart failure and arrhythmias becoming the most common cause of death.^448^

The cardiomyopathy of DMD and BMD is characterized by progressive loss of functional myocytes leading to regional dysfunction followed by global dysfunction. Although, the cardiomyopathy has been commonly described as having a dilatated phenotype, chamber dilation occurs only in the late stages of the disease. The cardiomyopathy is variable both in age of onset and severity. Histologically, the dystrophinopathies lead to alternating areas of myocyte hypertrophy, atrophy and necrosis, and finally fibrosis with replacement of cardiomyocytes by connective tissue and fat. It is estimated that ~ 30% are symptomatic, but elucidating symptoms from patients who are non-ambulatory secondary to skeletal muscle disease is very difficult.^449^ Although myocardial damage is present on a cellular or histological level starting very early in life, echocardiographic abnormalities are usually delayed until the second decade of life. For DMD, risk of LV dysfunction increases significantly with age, from < 5% for boys < 10 years of age to > 75% for men > 20 years of age.^450^ There are patients who will have reduced ejection fraction as early as 8 years of age.^451^ Subtle abnormalities of deformation using strain analysis can be seen in patients as young as 5 years.^452^

#### Indications for CMR

There are several guidelines available addressing the cardiovascular care of DMD/BMD patients.^453^ All recommend that cardiac care begin shortly after initial diagnosis is made. Since the risk of cardiovascular involvement is low for very young children, the initial evaluation consists of clinical evaluation, baseline ECG, and TTE. Yearly TTEs are recommended until the child is old enough to undergo CMR without sedation.

TTE has limitations in neuromuscular diseases.^454,455^ As patients age, the image quality is degraded due to several factors including spinal and thoracic bony deformities, increased thoracic and abdominal adiposity that decreases ultrasound penetration and alteration of the inter-rib spaces decreasing the size of acoustic windows. A study evaluating TTE image quality for 31 DMD patients aged 11–34 years showed that none of the apical four-chamber image acquisitions were of diagnostic quality.^456^ In a study designed specifically to assess the ability of TTE to assess DMD patients,^457^ they found that by 13 years of age, 50% of the studies were classified as suboptimal with ≥ 30% of segments inadequately visualized, and by 15 years of age, 78% of studies were suboptimal. Consequently, in this population, TTE data may not always accurately reflect cardiac function. Some of these limitations can be overcome by administration of ultrasound contrast,^458^ however, contrast echocardiography has not been included in the standard clinical care guidelines and is used only in patients who have suboptimal acoustic windows and contraindications to CMR. Secondary to the known difficulty with accurate TTE assessment, the first CMR studies for DMD cardiomyopathy patients were performed to accurately measure the LVEF where echocardiographic windows were poor.

CMR is a proven modality for accurate and reproducible assessment of both RV and LV volumes and masses that is not affected by body habitus or lung artifacts,^459,460^ so it is not surprising that it has been utilized for accurate measures of ventricular function in children with this diagnosis^454,455,461–464^ and to assess the efficacy of treatment.^465^ Although the use of CMR is very common in this patient population, there are few direct head-to-head comparisons of CMR with echocardiography. Brunklaus et al.^462^ studied 35 DMD boys (12–18 years; median 15 years) who underwent both TTE and CMR, and although echocardiographic shortening fraction correlated with CMR LVEF (rs = 0.67; p < 0.001), 75% of the TTE studies had deficient ultrasound scanning windows and in 26% measurements, either significantly over- or underestimated LV systolic function compared to CMR. Buddhe et al.^464^ studied 35 subjects with a mean age of 13.6 years old who also had both TTE and CMR and found, similar to the previous study, weak correlation of TTE parameters with CMR EF.

CMR strain imaging has been studied in patients with DMD and has been found useful to detect occult contractile dysfunction and dyssynchrony.^463,464,466–468^ Hagenbuch et al. found that serial monitoring of cardiac dysfunction by serial strain measurements was able to predict progression of the disease in the absence of deterioration of LVEF.^469^

The first large scale clinical report of CMR in DMD with both ventricular function and myocardial characterization using LGE came from Brazil in 2007,^462^ although there have been a number of other studies of LGE with ventricular function in pediatrics.^464,467^ Silva and colleagues showed the feasibility of using CMR for routine assessment of both ambulatory and non-ambulatory DMD patients and demonstrated for the first time that DMD patients have LGE in a pattern that appears to represent the known myocardial fibrofatty replacement. They showed that even patients with normal LVEF were positive for LGE down to age 8 years and that LVEF was markedly different between the groups with and without LGE, linking LGE with decreased LV performance. They noted the LGE was predominantly seen in the inferolateral and anterolateral segments, a finding that has long been known from early pathology studies.^470^

The first report of CMR in a small multi-institutional retrospective study in children with DMD showed LGE was prevalent,^471^ finding 32% with LGE primarily involving the basal inferolateral segment of the LV in a sub-epicardial distribution. Patients with LGE were older than those without (mean age 16.4 vs 12.9 years), but most importantly, they noted a similar finding to that seen by Silva et al.^461^ where LGE was inversely correlated with LVEF. A larger study of 314 DMD children by Hor et al. showed the overall prevalence of LGE was ~ 36%,^451^ increasing from 17% of patients < 10 years to 34% of those aged 10–15 years and 59% of those > 15 years-old. Ten percent (11/113) of patients who had LGE died an average of 10.8 months after CMR. Conversely, only one patient from the LGE negative group died. Patients who died had larger LV volume and greater number of positive LGE segments compared to those who remained alive.

CMR has been associated with outcomes for the DMD/BMD population. In young adults, Florian et al. prospectively studied 88 male DMD/BMD patients^472^ and during a mean follow-up time of 47 ± 18 months, the primary endpoint (death or transplantation) was observed in 3% and the secondary endpoint (hospitalization and/or ventricular tachycardia) in 24%. They found that LVEF and the presence of “transmural” LGE were independent predictors for secondary endpoints. Interestingly, in the group of patients with preserved function (LVEF > 45%), patients with “transmural” LGE had a significantly lower event-free-survival compared to those without. Similarly, in children and adolescents, Menon et al. found LGE present in 78% of the boys.^473^ Compared with patients without LGE, those with LGE were older with lower LVEF (46 ± 12 vs 56 ± 9% respectively) and a higher incidence of ventricular tachycardia (40 vs 0%, respectively). Interestingly, during the study period, six of the subjects (19%) died. The factors associated with mortality were increased age, advanced grade of LGE, higher LV end-systolic volume, lower LVEF, and ventricular tachycardia.

There are now longitudinal studies of CMR in DMD populations that help to inform the appropriate longitudinal timing for these exams. Tandon et al. reviewed 465 serial CMR DMD studies, all of whom had ≥ 4 assessments.^474^ They determined that LVEF declined ~ 0.58% per year independent of LGE status. More interestingly, LVEF did not decline over time if LGE was absent but declined at a rate of ~ 2.2% per year when LGE was present. The number of LGE-positive LV segments increased with age as well.

*Limitations of CMR as it Relates to DMD Patients* Although CMR imaging does not suffer from the limitation of poor acoustic windows attributable to body habitus, thoracic abnormalities, or lung disease, there are factors that do limit its utility for imaging every patient with DMD. Some DMD patients cannot be comfortably positioned on the CMR table because of significant contractures, severe back pain, or immobility which is particularly true for older, non-ambulatory patients. CMR image quality is limited in patients with atrial or ventricular arrhythmias, irregular respiratory rates or motion, or inability to remain motionless in the scanner. Although some of these limitations can be overcome with sedation or anesthesia, such studies come with increased risk in this patient population secondary to compromises in respiratory or cardiac function. A thorough risk–benefit analysis is required before undertaking a sedated CMR study for a DMD patient. Finally, there is potential for artifact leading to CMR image degradation from implanted devices in DMD patients including both cardiac electrophysiologic therapeutic devices such as pacemakers and ICDs as well as skeletal devices including spinal fixation rods.

#### Summary of Recommendations

- CMR should be used to evaluate biventricular size and systolic function of DMD and BMD patients after the age of 8 years (or when they do not need sedation) (Class I, Level of Evidence B) and may be performed every year if needed.
- CMR myocardial fibrosis evaluation of DMD and BMD patients is recommended for prognostication and risk stratification (Class I, Level of Evidence B).
- CMR strain analysis of DMD and BMD patients may be considered for prognostication and risk stratification (Class IIb, Level of Evidence B).

### Cardiac Tumors

#### Background

Cardiac tumors are rare in the pediatric population, with an incidence between 0.027 and 0.3%.^475,476^ Management requires differentiation of specific tumor types, an objective for which imaging plays a significant role. Echocardiography remains the primary modality for initial detection and screening of cardiac tumors though has a limited ability to further characterize the mass other than its presence with the exception of echogenic and echolucent (presumably cystic) regions. In addition, echocardiography can be limited by acoustic windows and reliable differentiation of tumor from vegetation or thrombus can be difficult. Many masses are benign but still need to be distinguished from malignancies, characterized for prognosis, assessed for impact on heart/valve function and determined whether an intervention is needed. Thus, additional imaging is generally warranted.

#### Indications for CMR

**Figure 45.**
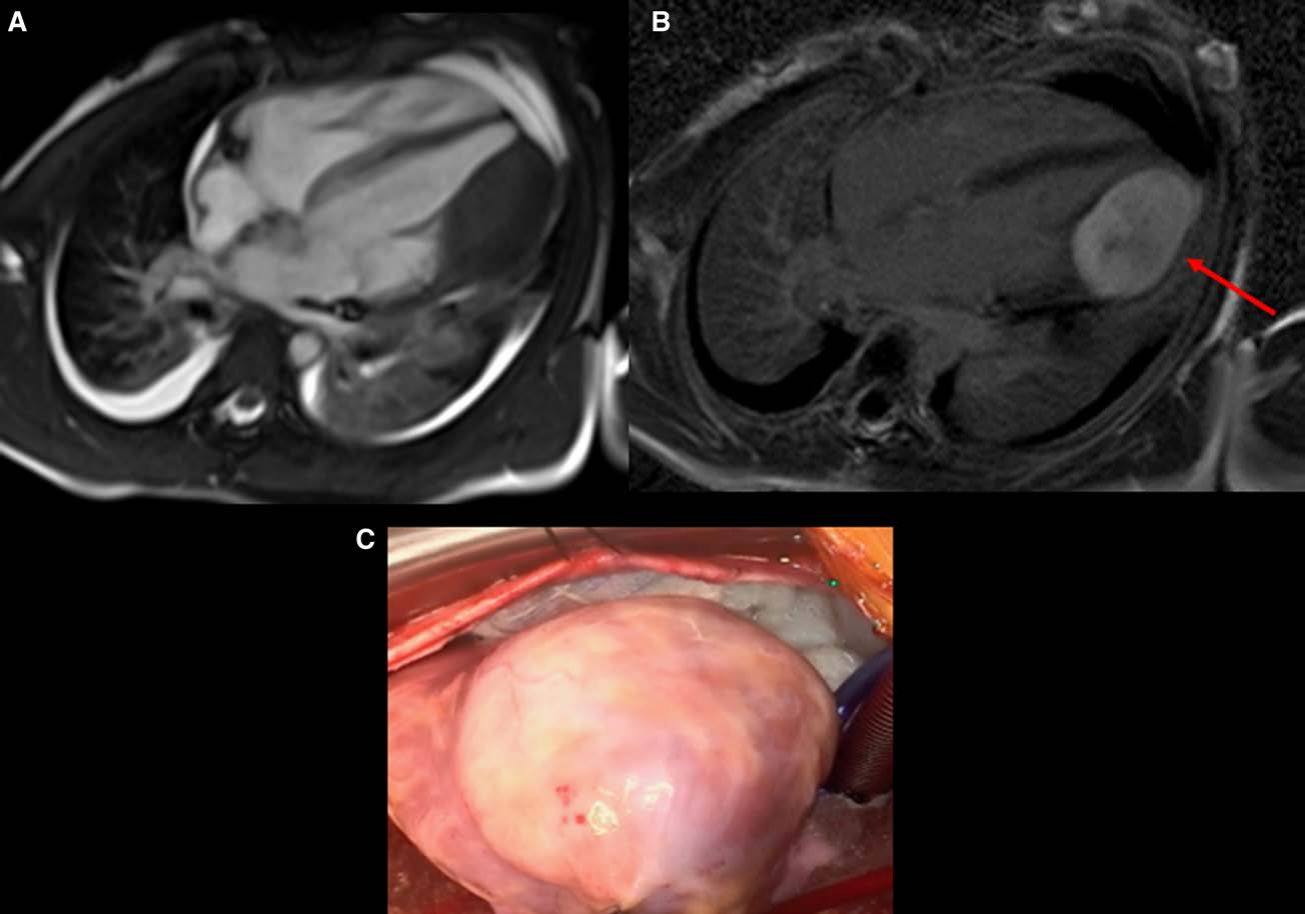
Cardiac fibroma in an 8-year-old male characterized by cardiovascular magnetic resonance. **A**, Still frame of bSSFP cine shows a large mass in LV free wall. **B**, Phase sensitive inversion recovery (PSIR) sequence positive for late gadolinium enhancement, characteristic of a cardiac fibroma. **C**, Mass surgically resected, pathology confirmed fibroma.

After identification of masses by echocardiography, CMR is utilized in a number of different ways (Figure 45) and has demonstrated utility for^477–482^:

- Precise definition of anatomic location of the tumor including size, sites of myocardial attachment, and tissue layers involved.
- Tissue characterization and differentiation between benign and malignant tumors as well as to differentiate the mass with thrombus.
- Differentiation between tumor and thrombus^483^
- Determining the extent of the tumor and relationship with surrounding structures to determine impact on heat and valve function and guide surgical resection (eg outflow tract obstruction).
- Imaging of fetal tumors

As certain genetic syndromes are also associated with cardiac tumors, CMR is especially useful in establishing the diagnosis when cardiac masses are noted on echocardiography in that patient population.^482^

Given the overall low incidence of cardiac tumors and the varied types of tumors in pediatrics, relevant published CMR experience includes case reports as well as relatively small case series.^484–491^ Other publications^492–498^ have provided reviews and experiences of various centers of the utility and approach to CMR imaging for pediatric masses and how the findings may direct care.

As noted in a prior consensus statement, the utility of CMR is its ability to describe location, size, hemodynamic effects, and tissue type of cardiac masses (thrombus, benign or malignant tumor)^2^ upon which clinical decisions are often based. Various publications have described tissue characterization techniques for differentiating benign tumors such as rhabdomyomas^499^ most commonly or myxomas^486,487^ from others such as osteoscarcoma,^490^ highly vascularized tumors such as hemanginomas,^488,491^ or adipose tissue such as lipoma.^489^

In the one published multicenter study comparing pediatric CMR to histologic diagnoses in a blinded fashion to demonstrate the ability of CMR ability to accurately differentiate cardiac masses,^477^ 97% of tumors were correctly identified compared to histologic diagnosis. Of these, a single correct diagnosis was made in 55% and a limited differential diagnosis which included the correct diagnosis was made in 42% (21% with 2 diagnoses and 23% with 3 or more diagnoses). There were only 2 cases of incorrect diagnoses, both having an atypical appearance on CMR. Generally, incorrect or incomplete diagnoses related either to atypical features of the mass, such as unusual location, or to technical imaging issues such as incomplete image acquisition or poor quality. This multicenter experience demonstrated the clinical utility of CMR in differentiating masses non-invasively, and thus its role in clinical management of affected patients (Table 7).

**Table 7.**
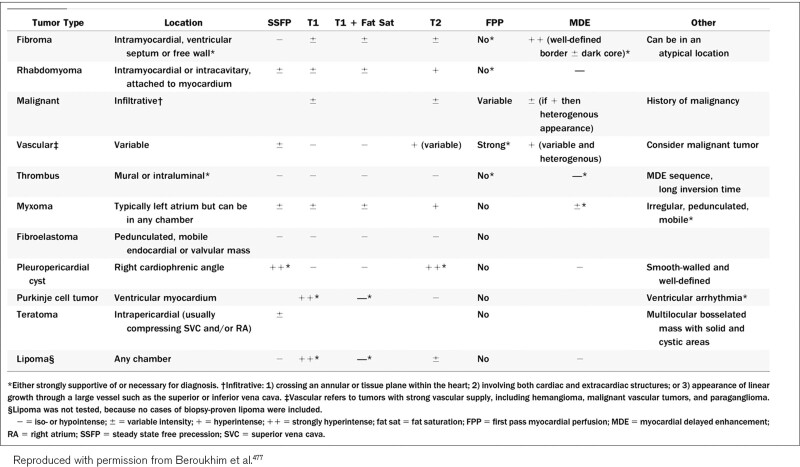
Tumor Characterization by Cardiac Magnetic Resonance

#### Summary of Recommendations

- CMR with gadolinium based contrast is indicated for the evaluation of cardiac masses for tumor type, characterization, accurate identification of location, size or hemodynamic effects of the mass or visualization of the mass for surgical planning (Class I, Level of Evidence B)
- CMR is indicated to distinguish between a benign and malignant cardiac tumor. (Class I, Level of Evidence B)
- CMR is indicated to distinguish cardiac tumor from thrombus. (Class I, Level of Evidence B)
- Non-contrast CMR is reasonable in patients with compromised renal function (eGFR < 30 ml/min/1.73 m^2^) for description of location, size, and any hemodynamic effect, but may be less useful for tissue characterization. (Class IIA, Level of Evidence C)

### Myocarditis

#### Background

Myocarditis is a potentially life-threatening disease and a significant cause of pediatric morbidity and mortality.^500,501^ At the same time, it remains a difficult disease to diagnose^501^ with limited literature in pediatrics. Definitive diagnosis of myocarditis can be made from endomyocardial biopsy (EMB) which remains the diagnostic gold standard and can help guide therapy.^501^ EMB, however, is prone to low sensitivity related to sampling error,^501,502^ practice variation^503^ or adverse events due to its invasive nature.^504,505^ Thus, the diagnosis of myocarditis in pediatrics is often made from a combination of clinical factors including the patient’s history, symptoms, ECG changes and serologic findings.^506^

Non-invasive imaging plays a key role in diagnosis, almost always starting with echocardiography, with the addition CMR imaging to vastly increase the diagnostic sensitivity and specificity.^506^ In adults, CMR has become a well-established modality for diagnosing myocarditis^507^ with recommendations by the International Consensus Group on CMR (ie the “Lake Louise” criteria) published in 2009^508^ and updated in 2018.^509^ Evidence suggests CMR is indicated in the diagnosis of pediatric myocarditis; as a matter of fact, one of the first publications of CMR findings and tissue characterization in myocarditis was in children in 1991 which also compared these findings to EMB.^510^

#### CMR Indication

In the clinical setting of acute chest pain and concerning diagnostic testing (ECG, elevated cardiac enzymes or other serology, etc.), a diagnosis of myocardial inflammation can significantly affect prognosis and management.^500,511^ CMR is useful in the setting of suspected myocarditis for:

- Confirming the presence of myocardial inflammation and edema
- Differentiating ischemic (i.e. coronary) from non-ischemic causes of myocardial inflammation.
- Risk stratification and prognostication
- Guiding subsequent investigations

#### Confirming the Presence of Myocardial Inflammation

TTE is usually the 1st non-invasive imaging test in the evaluation of suspected myocarditis and allows assessment of ventricular function, size, regional wall motion abnormalities, valvular abnormalities and pericardial effusion. The common TTE findings in myocarditis are usually non-specific though TTE can help distinguish between the different myocarditis phenotypes of fulminant myocarditis (severe dysfunction), acute (non-fulminant) myocarditis^512^ or dilated cardiomyopathy. Beyond these findings, however, echocardiography lacks the sensitivity and specificity to further characterize myocardial inflammation and a test with higher diagnostic accuracy is required for further clinical management.

**Figure 46.**
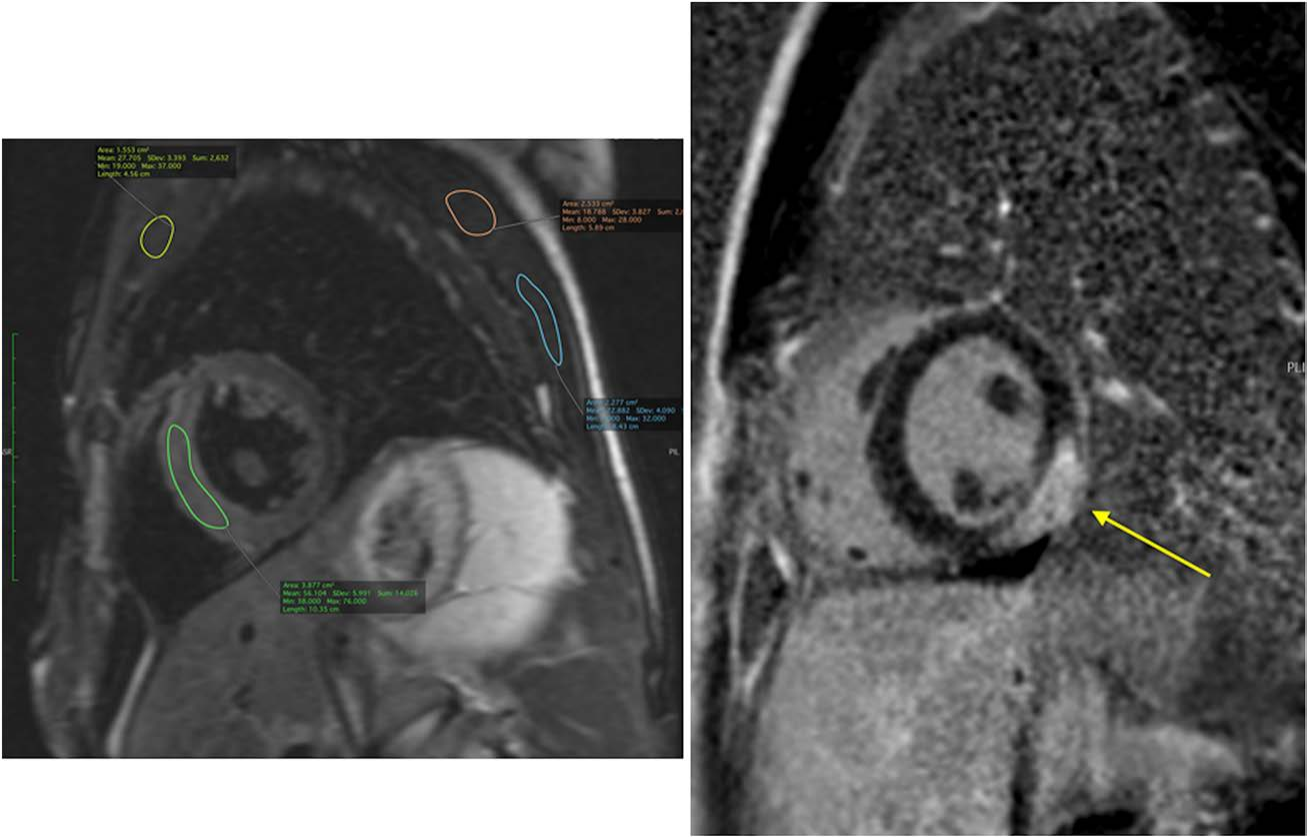
On the left is a CMR T2 weighted sequence showing > 2X enhancement of myocardium (green region of interest, ROI) compared to skeletal muscle (yellow, pink or blue ROI), meeting criteria for myocardial edema per 2009 Lake Louise criteria. On the right is a post-contrast phase sensitive inversion recovery (PSIR) sequence showing sub-epicardial late gadolinium enhancement indicating necrosis or scar (yellow arrow).

The value of CMR in myocarditis is to evaluate the tissue characteristics of myocardial inflammation; edema, hyperemia and capillary leak (using T1 and T2 weighted sequences) and necrosis or scarring of ventricular myocardium (Figure 46).^500,508^ Additional supportive criteria such as pericarditis, pericardia effusion or LV systolic dysfunction is also evaluated by CMR.^509^ The 2009 (aka “original”) Lake Louise criteria consists of:

- T2 weighted imaging showing the presence of myocardial edema.^513,514^
- T1-weighted images obtained before and early after administration of gadolinium-contrast revealing early gadolinium enhancement (EGE),^508^ consistent with myocardial hyperemia or inflammation.
- T1-weighted segmented inversion-recovery gradient-echo sequence^515^ showing LGE, characteristic of myocardial necrosis or scarring.

It also specified two positive findings (either T2 or T1 based) are necessary to diagnose myocarditis (Figure 46).

The Lake Louise criteria have been validated in pediatrics mainly from case reports^516^ or single-center series with relatively small populations.^510,517–520^ There has been one retrospective multicenter study that has evaluated CMR techniques in children with myocarditis which included 13 centers and 143 subjects^521^ and although there was variability in tissue characterization protocols among centers, overall sensitivity for CMR positive myocarditis was high (82%).

The 2009 Lake Louise criteria were updated in 2018 to include parametric mapping techniques that have significantly advanced in the diagnosis of myocardial inflammation in adult patients.^507,522,523^The updated recommendations included one T2 relaxation-based marker for myocardial edema (T2-mapping or T2 weighted images) and one T1 relaxation-based marker (abnormal T1, ECV or LGE) for associated myocardial injury.^510^ While the inclusion of multiparametric mapping is supported by a robust evidence base in adults, the mutiparametric mapping literature in pediatrics has been limited to small single-center studies.^430,524–527^ There is, however, emerging data about the potential added value of multiparametric mapping in the evaluation of pediatric myocarditis.^528^ The authors of the 2018 Lake Louise criteria currently state that “the original Lake Louise Criteria provide a good overall diagnostic performance, and thus they should remain in use in centers that have good experience with their application.” The MYKKE consortium is one effort currently underway to establish a multicenter registry with prospective data collection in pediatric myocarditis.^529^

#### Differentiating Ischemic from Non-ischemic Causes of Myocardial Inflammation

In the setting of myocarditis resembling an acute coronary syndrome with a young patient presenting acutely with chest pain, elevated cardiac enzymes and ECG abnormalities, CMR can be used to differentiate between myocarditis and myocardial infarction due to coronary artery disease:

- In myocarditis, the pattern of LGE enhancement is characteristically subepicardal, may be transmural (but usually in a non-coronary distribution), and usually in a patchy distribution.^530^
- The LGE pattern from coronary artery disease (myocardial infarction), is subendocardial or transmural and in a distribution of coronary perfusion territory.

Establishing the diagnosis of myocarditis in a patient with low risk for coronary artery disease (i.e. most pediatric patients) is helpful and avoids unnecessary cardiac catheterization for coronary angiography.

#### Risk Stratification and Prognostication

CMR can be useful for risk stratification with adult studies showing robust outcomes data on adverse cardiac events and arrhythmias.^531–536^ Similarly, CMR has been shown to be useful in evaluating outcomes in pediatrics^537,538^ including a large, multi-center study.^539^ Lastly, myocarditis has a known association with dilated cardiomyopathy (DCM), either as a precursor to DCM or as an acute illness that “unmasks” previously undetected DCM.^540^ Long-term studies in patients with acute myocarditis report the development of DCM in 21%, showing the utility of CMR for chronic management.^541^

#### Guiding Subsequent Investigations

Following the diagnosis of myocarditis by CMR, more detailed information may be needed from EMB to direct therapy. In this situation, there is diagnostic synergy between non-invasive CMR and invasive EMB to identify and treat particular etiologies of myocarditis. Examples may include giant cell or eosinophilic myocarditis that are typically treated with immunomodulator therapy.

#### Summary of Recommendations

- The original Lake Louise Criteria (2009) is recommended for diagnosing myocardial inflammation for centers that have a good experience with these criteria in the pediatric age range (Class I, Level of Evidence B).
- CMR with gadolinium-based contrast is indicated for evaluation of myocarditis to confirm the presence of myocardial inflammation in pediatric patients (Class I, Level of Evidence B).
- CMR is beneficial in children and adolescents for differentiating ischemic from non-ischemic causes of myocardial inflammation, risk stratification/prognostication, and guiding subsequent investigations (Class I, Level of Evidence B).
- CMR with multiparametric mapping can potentially add value in the evaluation of pediatric myocarditis, and more studies are needed in this population (Class IIa, Level of Evidence C).

## Function and hemodynamics

### Ventricular Function

#### Background

The end result of many congenital and pediatric heart lesions is ventricular dysfunction which may result in much of the morbidity and mortality associated with these diseases. Therefore, quantifying ventricular function is a vital aspect of continuing assessment.

At the simplest level, cardiac failure is defined as an inability to produce adequate cardiac output to supply the bodies metabolic needs. Thus, measurement of cardiac output can provide important information regarding cardiac function. The traditional reference standard method of measuring cardiac output is at cardiac catheterization using either thermodilution or the Fick method. Unfortunately, invasive assessment is associated with not insignificant risk as well as cost and is therefore not performed routinely. More importantly, most CHD patients have normal baseline cardiac output due to cardiac compensation (e.g. ventricular dilation, hypertrophy and increased filling pressures). Many symptoms are actually related to these compensatory mechanisms as well as an inability to augment cardiac output during exercise. Assessment of many of these compensatory mechanisms requires visualization of the ventricles and therefore, cardiac imaging is vital.

The most ubiquitous cardiac imaging modality used in CHD and acquired pediatric heart disease is echocardiography which has many advantages over other imaging techniques and is considered the first line method of assessing cardiac size and function. Nevertheless, echocardiography still has some significant problems such as operator dependence, poor acoustic windows and poor blood pool to myocardial contrast. In the last 20 years, as mentioned, CMR has become the reference standard method of assessing biventricular function.^3,32,102–106,272,309^

### Indications for CMR

#### Ventricular Volumes and Ejection Fraction (Figures 4 and 40)

Many CHD lesions are associated with volume loading due to shunts or valvular regurgitation. This is true in not only such common shunt lesions such as ASDs, VSDs and patent ductus arteriosus but in more complex and less common lesions such as anomalous pulmonary venous connections or left SVC connected to the left atrium. Similarly, valvular regurgitation in patients with BAV, cleft mitral valve or TR associated with HLHS can cause significant volume loads. The cardiac response to volume loading is dilation which can result in increased risk of arrhythmia, increased wall stress and predisposition to dysfunction. Thus, measuring ventricular volumes is an important component of evaluating CHD.

Although less common, small ventricular volumes also need to be evaluated. Varying degrees of hypoplasia are seen in diseases such as HLHS, critical aortic stenosis, double outlet RV and malaligned atrioventricular canals to name a few. There has been a number of studies which attempt to aid in management of these patients, however, many questions remain.^542–544^

There are other lesions which occur in pediatric and CHD where the assessment of biventricular performance is important. Patients with anomalous coronary artery origins and courses, patients with surgically manipulated coronary arteries (see section on Coronary arteries), and patients with myocarditis (see Myocarditis section) are all examples where ventricular performance are useful.

Although ventricular volumes can be estimated by 2D echocardiography, most methods rely on significant geometric assumptions. These may hold true for the normal LV but are inadequate for the crescentic RV^545^ or the malformed LV in CHD. Newer 3D echocardiographic techniques do hold promise for true volumetric assessment, however, they still suffer from operator dependence and potentially inadequate acoustic windows. CMR has some distinct advantages over echocardiography. Firstly, it is not limited by body habitus and can be acquired in any patient irrespective of size. Secondly, unrestricted 2D imaging can be performed, allowing imaging of contiguous slices covering both ventricles. The result is true 3D measurement of ventricular volumes.^546^ Further, although there are CMR techniques that can be real-time (ie instantaneous acquisition), cine imaging for ventricular function is built over multiple heart beats, averaging the data in the image; whereas in echocardiography or catheterization, the imager is required to average the ventricular function in their minds, CMR averages it in the image itself and the quantification is a more true reflection of typical ventricular performance in the patient. Finally, modern bSSFP CMR techniques provide high blood pool myocardial contrast, aiding segmentation and further post-processing.^547^

An important benefit of accurate volume assessment is that it also enables precise measurement of ejection fraction which is a well-recognized measure of cardiac function and ventricular arterial coupling and an important prognostic marker in many forms of CHD and pediatric cardiovascular disease.^109,548,549^

Another metric that can be accurately evaluated from this sort of data is ventricular mass. Increased ventricular mass is common in patients with obstructive lesions (e.g. aortic stenosis or PA stenosis) or in for example, the RV of HLHS or TOF. As CMR allows visualization of the whole ventricular mass, especially the RV and its free wall, it enables very accurate measurement which is key to understanding the physiology and managing patients.

Several studies have shown that CMR provides highly accurate and reproducible measurement of ventricular volumes, mass and ejection fraction data in CHD.^159,550,551^ For these reasons CMR is now be considered the reference standard method of measuring LV and RV volumetric data.

Several studies have demonstrated that CMR measurements of ventricular volumes, mass and ejection fraction provide important clinical information in conditions such as repaired TOF,^548,549^SV at different stages of the Fontan palliation (Figure 4)^36^ and pediatric pulmonary hypertension^549^ to name a few. This includes prediction of outcome (e.g. mortality or major cardiac event) and exercise tolerance, as well as response to interventions (e.g. valve replacement).^552^

There are some limitations in using CMR to measures ventricular volumes and ejection fraction. In some patients, particularly children, breath holding is difficult and free breathing approaches are required including signal averaged and real-time acquisitions. Real-time sequences are becoming increasingly used in pediatric imaging and with the advent of new accelerated techniques, are now reaching the quality of conventional breath hold cines.^553^ Studies have validated real-time CMR for measurement of ventricular volumes and they should be considered a possible alternative to conventional imaging. Real-time imaging can also be useful in patients with irregular cardiac rhythm, although no large-scale studies demonstrating utility have been performed. Motion correction techniques, now in common use, can be used to increase image quality and negate respiratory artifacts.

#### Ventricular Filling and Diastolic Dysfunction

Diastolic dysfunction is often overlooked as a cause of symptoms in CHD. Evaluation of diastolic dysfunction requires measurement of the dynamic aspects of ventricular filling. Thus, a limitation of conventional ventricular volume and ejection fraction assessment is that it provides little information regarding diastolic function. The traditional method of assessing diastolic function is echocardiography assessment of atrioventricular valve inflow. Specifically, the ratio of peak early to late inflow velocities (E/A ratio) is an important indicator of ventricular diastolic dysfunction. The E/A ratio can also be assess using PC-CMR and some studies have demonstrated clinical utility.^554^ However, this should be considered a subsidiary measurement rather than the main reason to perform CMR. A better method of assessing systolic and diastolic function may be to directly measure myocardial motion in diastole and myocardial velocities have been measured by CMR and validated against echocardiography.^555^

#### Myocardial Motion

Volumetric measurement enables evaluation of global changes to ventricular volume and function. However, assessment of volumes alone does provide the full picture of cardiac dysfunction. There is evidence that both systolic and diastolic early ventricular dysfunction is better identified by evaluating the specific aspects of local myocardial motion. Although the cardiac myocyte can only shorten, the heart has complex local motion due to the specific arrangement of myocytes. This includes longitudinal and radial contraction, twisting and wall thickening. Some or all of these metrics can be measured with echocardiography using tissue Doppler or strain imaging. However, like all echocardiographic techniques, they may suffer from inadequate windows and are always operator dependence. There are also several CMR techniques that can be used to evaluate myocardial motion such as myocardial tagging,^556,557^ tissue phase mapping and strain/displacement encoding. These techniques have been used to better understand pathophysiology in CHD. However, significant clinical utility has not been demonstrated and a number of sequences are still at the research stage.

More recently, strain (and strain rate) data has been derived from conventional CMR cines using feature and tissue tracking,^558,559^ processed using the same tools used for echocardiography strain imaging. The main benefit of this approach is that imaging acquired for conventional volumetric analysis can also provide local motion data. This has allowed retrospective analysis of historical cine data and demonstration of clinical utility. Several studies have now shown that strain metrics can independently predict outcome (e.g. death or major cardiac event) and exercise tolerance in patients with CHD.^560–562^ However, no large scale studies have demonstrated that CMR strain measures outperform conventional ventricular volumes or ejection fraction data. Thus, currently, strain assessment should be considered as an addition to rather than a replacement of conventional ventricular volumes.

Regional ventricular wall motion is important in pediatric heart disease patients such as AAOCA, surgically manipulated coronary arteries, myocarditis, arrhythmogenic RV cardiomyopathy and other lesions which occur in pediatric and CHD. This may be performed qualitatively on cine imaging or using quantitative measures such as myocardial strain.

Finally, tissue characterization such as LGE and diffuse fibrosis can add another dimension to the assessment of ventricular function, elucidating the etiology of regional or global myocardial dysfunction (see previous sections).

#### Summary of Recommendations

- CMR should be performed for the evaluation of biventricular volumes, mass and ejection fraction in patients with volume or pressure overload lesions as well as with varying degrees of RV or LV hypoplasia (Class I, Level of Evidence B).
- CMR is useful in assessing biventricular volumes and ejection fraction in patients with non-structural pediatric heart disease (e.g. DMD, myocarditis or pulmonary hypertension) or in those patients with congenital or surgically manipulated coronary artery lesions (Class I, Level of Evidence B).
- CMR is indicated in assessing ventricular volumes and ejection fraction in patients with SV (Class I, Level of Evidence B).
- CMR strain analysis may be considered in assessing ventricular function in CHD and prognostication of outcome (Class IIB, Level of Evidence B).
- CMR tissue characterization is reasonable in assessing ventricular function in CHD and prognostication of outcome (Class IIA, Level of Evidence B)

### Systemic to Pulmonary Blood Flow Ratio and Collateral Flow

#### Background

Cardiovascular shunts between the systemic and pulmonary circulations are the commonest result of CHD, accounting for approximately 50% of all cases.^563^ Shunts are characterized in 3 main ways: (i) location, (ii) magnitude and direction, and (iii) effect on ventricular volume and function. This section addresses evaluation of shunt magnitude and direction by calculation of the systemic and pulmonary blood flow ratio (Qp/Qs). A left-to-right shunt will result in greater blood flow to the pulmonary vasculature compared to the systemic vasculature and a Qp/Qs ratio > 1 (higher Qp/Qs indicates a larger shunt). Conversely, a right-to-left shunt will result in greater flow to the systemic vasculature and thus a Qp/Qs ratio < 1 (the closer the Qp/QS is to zero, the larger the shunt). Quantifying Qp/Qs is vital in deciding appropriate management of patients with shunt lesions and is included in most current international guidelines.^564^

The reference standard method of measuring Qp/Qs is invasive oximetry with measurement of oxygen saturations (or content) in the pulmonary and systemic arterial and venous systems. This method should be used if measurement of PA pressure and pulmonary vascular resistance are also required (e.g., Eisenmenger physiology). However, there are some disadvantages to catheter based evaluation of Qp/Qs. The most obvious are its invasive nature which can associated be morbidity and high cost. Furthermore, there, some technical problems with invasive oximetry that should be understood. Firstly, the requirement for measurement of oxygen saturation from multiple sampling sites during steady state can result in significant error propagation and inaccurate Qp/Qs quantification.^565^ Secondly, quantification of Qp/QS is only possible if sampling can be performed distal to the shunt which is not possible in some extracardiac lesions (e.g., systemic-pulmonary arterial collaterals). Finally, in some lesions such a SV after BDG, it is impossible to obtain adequate mixed systemic venous saturation as it is separated into SVC and IVC circuits.^43^ For all these reasons, non-invasive assessment of Qp/QS is becoming increasingly important in the assessment of shunt lesions.

The simplest non-invasive method is Doppler echocardiography with several techniques based on vessel area and blood flow velocity being described.^566^ The main benefit of this approach is that echocardiography is already the first line method of anatomically evaluating shunt lesions. Unfortunately, echocardiographic evaluation of shunt magnitude is prone to inaccuracy due to inadequate data acquisition and invalid assumption in the calculation.^567^

Collateral flow in CHD comes in 4 broad categories: (1) systemic to pulmonary (Figure 3), (2) veno-venous collaterals as found, for example, in patients with SV, (3) aortic collaterals which develop, for example, in patients with coarctation (Figure 29) and (4) coronary collaterals; these guidelines will not address coronary collaterals. Systemic-to-pulmonary collaterals have been known for many years to develop in SV patients,^22^ however, precise measurement of flow in these vessels remained a challenge up until the past 10 years. Their clinical impact has been debated in the past^568^ in part due to the challenge of quantification, although it is clear now that there is an effect on patient care and management^49,569–571^; it has also been demonstrated by pilot data that these collaterals can be mostly obliterated in the catheterization lab with a measured decrease in the collateral flow.^44^ Similarly, veno-venous collaterals are found not uncommonly in SV patients, from 20 to 33%,^572,573^ and interventions have some time been needed. Finally, known since the 1930s and 1940s,^574^ aortic collaterals develop from existing vessels such as intercostal and mammary arteries to bypass aortic obstructions such as coarctation of the aorta which clearly has clinical implications for the diagnosis and treatment of the disease.

#### Indications for CMR

Recently, CMR has become the non-invasive reference standard method of measuring Qp/Qs using PC-CMR which has been shown to allow highly accurate and reproducible quantification of Qp/Qs. In PC-CMR, through plane flow is directly measured without assumptions—which has been borne out by the significant number of studies that have demonstrated good agreement between PC-CMR and both direct measurement of flow in phantom studies^575,576^ and invasive oximetry in patient studies.^577–579^ A further benefit of PC-CMR over invasive oximetry is that measurement Qp and Qs is not limited to assessment of flow in the pulmonary trunk and aorta respectively. For instance, Qp and Qs can be quantified by measuring flow in the PV and vena cavae respectively. These alternative calculations are particularly useful in situations where: (i) it is not possible to measure blood flow in one of the great arteries (i.e. artifact obscuring the pulmonary trunk in a patient with prosthetic valve containing metal) and (ii) invasive oximetry fails, such as patients with systemic-to-pulmonary arterial collaterals. Finally, and maybe more importantly for the accuracy of the technique, these multiple methods of evaluating Qp and Qs can be performed in the same patient, providing key internal quality assurances.

An important additional benefit of CMR is the ability to accurately measure ventricular volumes (see Ventricular function section). It is possible to measure Qp/Qs directly using ventricular stroke volumes. However, shunt location must be taken into consideration. An ASD and VSD may both have a Qp/Qs of 2:1, but RV stroke volume will be twice the LV stroke volume in the ASD, while LV stroke volume will be twice the RV stroke volume in the VSD. Thus, without knowledge of shunt location, ventricular stroke volume should be used with caution. Furthermore, accurate assessment of Qp/Qs using ventricular stroke volume relies on competence of the atrioventricular and semilunar valves. Consequently, measurement of ventricular volumes is not the first line method of calculating Qp/QS. Nevertheless, ventricular stroke volumes do provide a way of performing internal quality assurance of the PC-CMR calculation. More importantly, evaluation of ventricular size and function allows accurate evaluation of the physiological sequelae of a shunt lesion (e.g., RV volume loading in an ASD).

Another important advantage of CMR is the ability to anatomically delineate shunts. Studies have shown that CMR can provide definitive diagnosis and evaluation, for example, of sinus venosus defects and anomalous PV connections (see PV section).^580,581^ This is relevant as these lesions are difficult to image with echocardiography, particularly in older patients. Several studies have also shown that CMR provides comprehensive evaluation of secundum ASDs and can determine candidacy for transcatheter or surgical closure.^582–584^ Patients with VSDs are generally diagnosed and managed using echocardiography, however, CMR with 3D imaging may useful for delineation of complex or multiple defects.^585^ Finally, CMR can be useful in delineating the anatomy of patent ductus arteriosus and aorto-pulmonary windows in older patients, where echocardiography can be insufficient.

With all the possible shunt lesions in CHD other than the ones listed above such a truncus arteriosus, double outlet RV, TOF, it is not feasible to list them all in this guidance. Suffice it to say that when clinically important shunt lesions are present, imaging with CMR is useful.

Systemic-to-pulmonary collateral vessels can be visualized by CMR most notably by administering contrast (Figure 3) and flow in these vessels was first quantified by CMR in 2009.^23,45^ CMR is the only methodology that can accurately quantify this flow.^43^ The use of CMR to quantify this flow has demonstrated that these collaterals do have a measurable effect on outcome^46,49,569–571^ and act to steal blood from key organs such as the brain.^47^ CMR has been able to quantify the short term decrease in systemic-to-pulmonary collateral flow after embolization in the catheterization lab.^44^

CMR has also been extensively used to quantify collateral flow around a coarctation site (Figure 29) which is calculated by measuring flow volumes in the aorta near the coarctation and at the diaphragm.^255,256,258–260^ An increased flow volume at the diaphragm indicates significant collateral flow into the aorta, bypassing the narrowing (see Coarctation of the aorta section).^258^ Venovenous collaterals are also imaged by CMR^586–588^ and their presence needs to be taken under consideration prior to intervention.

#### Summary of Recommendations

- CMR should be used for the evaluation of the magnitude and direction of intracardiac shunts in children and adults such as with ASDs and VSDs as examples (Class I, Level of Evidence B).
- CMR is indicated for the evaluation of the magnitude and direction of extracardiac shunts in children and adults such as with patent ductus arteriosus and systemic-to-pulmonary collaterals as in Fontan patients (Class I, Level of Evidence B).
- CMR is beneficial for the anatomic and quantitative flow assessment of systemic-to-pulmonary collaterals, aortic and venovenous collaterals (Class I, Level of Evidence B).

### Late Gadolinium Enhancement (LGE)

#### Background

CMR is an integral part of the non-invasive imaging of both acquired and CHD lesions in children for the evaluation of complex cardiac anatomy, quantification of ventricular and valvular function, and myocardial characterization. A primary mode of myocardial characterization is with post-contrast LGE imaging.^115^ The principle of this technique is that gadolinium-based contrast agents have increased volume of distribution in abnormal myocardium. The difference between normal and abnormal tissue distribution volume (Vd) results in a CMR signal difference. The mechanism of increased Vd is variable. In inflammatory disease such as acute myocarditis or acute ischemia, the increased volume of distribution results from myocardial cell injury or death. In chronic myocardial infarction or fibrosis in cardiomyopathies, the extracellular space is expanded.

The standard LGE imaging is performed ~10 min following intravenous administration of 0.1–0.2 mmol/kg of gadolinium-based contrast agent (GBCA). Because of increased cardiac output, LGE imaging in pediatrics can start as early as 6 min after infusion. It should be noted that due to the risk of nephrogenic systemic fibrosis, the use of GBCA should be used with caution in acute or chronic renal disease. Multiple versions of the sequence have been developed since it was first introduced.^589^

#### Indications for CMR

*Masses (see Tumor section*) CMR with LGE imaging is very useful in the evaluation of pediatric cardiac masses because of its tissue characterization capabilities, allowing a narrowing of differential diagnoses. Cardiac mass evaluation includes standard cine, T1-weighted, and T2-weighted, perfusion and LGE sequences. Using LGE in a comprehensive study of cardiac masses adds significantly to the differentiation of common tumor types.^477^ This is particularly true for fibroma that shows marked enhancement. In addition, CMR using LGE and a TI scout following gadolinium administration is highly accurate in discerning tumor versus chronic thrombus.^483^

*Myocarditis and Other Inflammatory Diseases (See Myocarditis Section*) LGE is a primary component of the CMR myocarditis evaluation..^508,509^ The presence of LGE in patients with suspected myocarditis has a high specificity with the signal thought to represent areas of necrosis or fibrosis. The LGE lesions are typically patchy, subepicardial, located in the basal or mid-ventricular segments, and are not in a coronary distribution. The median area under the ROC curve for LGE alone in myocarditis is 83% but that is much improved when other criteria are taken into account. It is sensitive for acute myocarditis with large severely affected areas. It is not sensitive for mild cases and does not yield information regarding acute edema or active inflammation.

*CHD* In a variety of both unrepaired and repaired CHD, LGE imaging has been investigated (Figures 5, 23, 42, 43) and has played a significant role.^590,591^ When extensive LGE is seen following surgeries for CHD, it is generally associated with adverse prognosis such as in systemic RVs (see below and ccTGA section). Scarring can be an iatrogenic complication when a coronary is injured or there is a problem with cardiopulmonary bypass or myocardial protection. The following lists some of the applications in CHD but is certainly not comprehensive.

In TOF (see TOF section), LGE is normally detected in the RVOT (and occasionally at the VSD patch), particularly following transannular patch repair, but occasionally extends beyond the site.^117^ A greater degree of RV LGE has been shown to be associated with arrhythmias and lower exercise capacity, all of which imply poor prognosis.^118^

LGE is seen in patients following both the ASO and atrial switch procedure for TGA (see TGA section).^172,365^ LGE can be seen because of myocardial fibrosis from either preoperative hypoxemia or from demand–supply mismatch caused by increased myocardial mass or decreased myocardial flow reserve. The extent of LGE correlates with age, RV dysfunction, QRS duration, QT dispersion and clinical events (arrhythmia and sudden cardiac death).

LGE has been seen in 28% of patients who had the Fontan operation^50^ and can be seen in the LV of patients with HLHS as endocardial fibroelastosis (see SV section). Presence and extent of LGE correlate with lower ejection fraction, dilated and hypertrophied systemic ventricle, regional wall motion abnormalities and NSVT; however, positive LGE is shown not to be associated with clinical endpoints (i.e. death or listing for cardiac transplant).

Congenital aortic valvular stenosis in children is caused by developmentally abnormal dysplastic aortic valve. Severe aortic stenosis can result in myocardial fibrosis manifested as LGE in a diffuse subendocardial pattern.^592^ Subendocardial LGE in adolescents who underwent balloon valvuloplasty in infancy corresponds to fibroelastosis on pathology and is associated with diastolic dysfunction.

*Ischemic Lesions (See Coronary Section*) Although rare in children and adolescents, myocardial infarctions occur as a result of cardiac surgery, thrombotic events, inflammatory heart disease (including Kawasaki disease) and hypercoagulable states.^593,594^ This becomes even more important with patients who have congenital coronary abnormalities (eg anomalous coronary arteries) or who have had coronary manipulation due to CHD (e.g., TGA after ASO). CMR has an important role in the evaluation of myocardial infarction and LGE is useful in establishing the diagnosis and extent of ischemic myocardial injury. LGE in a myocardial infarct is always subendocardial extending toward the epicardium and becoming transmural in severe infarctions. LGE can be used to quantify the scar either in a qualitative or quantitative manner. There is an inverse correlation between the extent of scar and recovery of contractile function.

*Pericardial Disease* CMR is used in the diagnosis and management of both acute and chronic pericarditis. Acute pericarditis manifests with pericardial thickening (> 4 mm) and pericardial effusion. Most standard acute pericarditis does not require CMR. LGE imaging shows focal or diffuse pericardial enhancement.^517^ More complicated cases of recurrent or chronic pericarditis often require advanced imaging and studies have shown the correlation between pericardial enhancement and systemic inflammation. Chronic pericarditis results in restriction from non-compliant and thickened pericardium. In patients with constrictive pericarditis who have LGE as evidence of pericardial inflammation, aggressive anti-inflammatory therapy may result in resolution without the need for surgery.

#### Cardiomyopathies

*Hypertrophic Cardiomyopathy (See Hypertrophic Cardiomyopathy Section*) LGE is seen in 40–80% of adults with HCM. Recent studies in children have shown LGE in 28–73% of patients in a similar pattern as in adults. LGE has been shown to have diagnostic importance for both diagnosis and management. It is correlated with adverse clinical outcomes including arrhythmia and SCD in both children and adults.^416,417,419^ There is a significant relationship between the extent of LGE and the presence of NSVT.

*Dilated Cardiomyopathy* CMR demonstrates linear mid-myocardial LGE in a non-vascular distribution in patients with idiopathic DCM. The presence of LGE, regardless of its extent or distribution, is associated with adverse prognosis such as SCD. In children, a study performed on a group with DCM showed the presence of LGE in only 16%.^595^

*Neuromuscular Disease* CMR is the standard for longitudinal evaluation of neuromuscular disease patients as they age and echocardiography becomes more difficult. LGE is an important part of these exams as increase in signal likely represents fibrofatty replacement of myocardium and portends onset of declining function.^474^

#### Summary of Recommendations

- CMR with LGE is recommended for evaluation of cardiac tumors or masses. (Class I, Level of evidence B)
- CMR with LGE is indicated for assessment of suspected myocarditis (Class I, Level of evidence B)
- CMR with LGE is reasonable for assessment of post-operative CHD (Class I, Level of evidence B)
- CMR with LGE is indicated for assessment of pediatric HCM, dilated cardiomyopathy and neuromuscular disease patients (Class I, Level of evidence B)
- CMR with LGE should be used for myocardial viability assessment in pediatric patients with decreased ventricular function, suspicion of a CHD, acquired, or iatrogenic coronary lesions (Class I, Level of evidence B).
- CMR with LGE can be beneficial for assessment of chronic pericarditis when the diagnosis or therapeutic strategy is unclear (Class IIa, Level of evidence B)

## Article Information

### Acknowledgements

Not applicable.

### Authors’ Contributions

All authors contributed substantially to the research, writing and editing of these guidelines. All recommendations were voted on by the writing committee. All authors read and approved the final manuscript.

### Sources of Funding

There was no funding for this guidelines manuscript.

### Availability of Data and Materials

Data sharing is not applicable to this article as no datasets were generated or analyzed during the current study.

### Ethics Approval and Consent to Participate

Not applicable.

### Consent for Publication

Not applicable.

### Disclosures

The authors declare that they have no competing interests.
